# *Candidatus* Frankia Datiscae Dg1, the Actinobacterial Microsymbiont of *Datisca glomerata*, Expresses the Canonical *nod* Genes *nodABC* in Symbiosis with Its Host Plant

**DOI:** 10.1371/journal.pone.0127630

**Published:** 2015-05-28

**Authors:** Tomas Persson, Kai Battenberg, Irina V. Demina, Theoden Vigil-Stenman, Brian Vanden Heuvel, Petar Pujic, Marc T. Facciotti, Elizabeth G. Wilbanks, Anna O'Brien, Pascale Fournier, Maria Antonia Cruz Hernandez, Alberto Mendoza Herrera, Claudine Médigue, Philippe Normand, Katharina Pawlowski, Alison M. Berry

**Affiliations:** 1 Department of Ecology, Environment and Plant Sciences, Lilla Frescati, Stockholm University, 106 91, Stockholm, Sweden; 2 Department of Plant Sciences, University of California Davis, Davis, California, 95616, United States of America; 3 Department of Biology, Colorado State University, Pueblo, Colorado, 81001, United States of America; 4 Université Lyon 1, Université Lyon, CNRS, Ecologie Microbienne UMR5557, 69622, Villeurbanne Cedex, France; 5 Department of Biomedical Engineering, University of California Davis, Davis, California, 95616, United States of America; 6 UC Davis Genome Center, University of California Davis, Davis, California, 95616, United States of America; 7 Centro de Biotecnología Genómica, Instituto Politécnico Nacional, 88710, Reynosa, Tamaulipas, Mexico; 8 Genoscope, Évry, France; Leibniz-Institute for Vegetable and Ornamental Crops, GERMANY

## Abstract

*Frankia* strains are nitrogen-fixing soil actinobacteria that can form root symbioses with actinorhizal plants. Phylogenetically, symbiotic frankiae can be divided into three clusters, and this division also corresponds to host specificity groups. The strains of cluster II which form symbioses with actinorhizal Rosales and Cucurbitales, thus displaying a broad host range, show suprisingly low genetic diversity and to date can not be cultured. The genome of the first representative of this cluster, *Candidatus* Frankia datiscae Dg1 (Dg1), a microsymbiont of *Datisca glomerata*, was recently sequenced. A phylogenetic analysis of 50 different housekeeping genes of Dg1 and three published *Frankia* genomes showed that cluster II is basal among the symbiotic *Frankia* clusters. Detailed analysis showed that nodules of *D*. *glomerata*, independent of the origin of the inoculum, contain several closely related cluster II *Frankia* operational taxonomic units. Actinorhizal plants and legumes both belong to the nitrogen-fixing plant clade, and bacterial signaling in both groups involves the common symbiotic pathway also used by arbuscular mycorrhizal fungi. However, so far, no molecules resembling rhizobial Nod factors could be isolated from *Frankia* cultures. Alone among Frankia genomes available to date, the genome of Dg1 contains the canonical *nod* genes *nodA*, *nodB* and *nodC* known from rhizobia, and these genes are arranged in two operons which are expressed in *D*. *glomerata* nodules. Furthermore, *Frankia* Dg1 *nodC* was able to partially complement a *Rhizobium leguminosarum* A34 *nodC*::Tn5 mutant. Phylogenetic analysis showed that Dg1 Nod proteins are positioned at the root of both α- and β-rhizobial NodABC proteins. NodA-like acyl transferases were found across the phylum Actinobacteria, but among Proteobacteria only in nodulators. Taken together, our evidence indicates an Actinobacterial origin of rhizobial Nod factors.

## Introduction

Actinorhizal root nodule symbioses are formed between nitrogen-fixing actinobacteria from the genus *Frankia* and a diverse group of mostly woody dicotyledonous plants from 23 genera and eight different families [1]. These families group in three orders, Fagales (Betulaceae, Casuarinaceae and Myricaceae), Rosales (Elaeagnaceae, Rhamnaceae and Rosaceae) and Cucurbitales (Coriariaceae and Datiscaceae). These plants, together with the legumes and *Parasponia* (Cannabaceae) which establish root nodule symbioses with rhizobia, belong to a clade of angiosperms known as the nitrogen-fixing clade (NFC; [2]). The scattered distribution of nitrogen-fixing plants within this relatively small clade supports a common origin of the predisposition to evolve root nodule symbioses assumed to have arisen ca. 100 million years ago (mya; [3,4]). This presumed gain-of-function evolved only once; however, among the plants with the predisposition, subsequently nitrogen-fixing root nodule symbioses evolved several times independently, with common themes but differences in infection process, nodule structure, nodule physiology and microsymbiont specificity. Using a database of nearly 3500 species within the NFC and the angiosperm phylogeny of Zanne et al. [5], Werner et al. [4] tested a series of models and came to the conclusion that the earliest root-nodule symbiosis arose in the common ancestors of actinorhizal Cucurbitales, followed by those of legumes, followed by actinorhizal Casuarinaceae and Rosaceae, and then by the other groups of actinorhizal plants.

Based on 16S rDNA phylogeny, the genus *Frankia* has been divided into four clusters. The basal group consists of so-called ‘atypical’ or ‘*Frankia*-like’ strains that have been isolated from nodules but cannot induce nodule formation, also called Cluster IV. This group seems to be closely related to rhizosphere strains previously detected only by direct amplification of 16S rDNA [6], and forms a heterologous group of *Frankia* strains with the highest diversity [7]. Symbiotic *Frankia* strains make up the other three clusters, which also correspond in general to host specificity groups. While cross inoculation may be possible within a cluster, generally not all strains of a cluster can colonize all of the cluster’s host plants [7]. Cluster I is the largest and most divergent one; its members are capable of colonizing species of the three actinorhizal families of the order Fagales, Betulaceae, Casuarinaceae and Myricaceae. Cluster III strains form nitrogen-fixing root nodule symbioses with the host-plant genus *Gymnostoma* (Casuarinaceae, in Fagales), Elaeagnaceae (Rosales), and all actinorhizal genera of the Rhamnaceae (Rosales) except *Ceanothus*. Cluster II strains nodulate members of the families Coriariaceae and Datiscaceae (Cucurbitales); as well as all the actinorhizal members of the Rosaceae and *Ceanothus*. In contrast with strains of Clusters I and III, Cluster II strains could so far not be cultured despite numerous attempts, leading to the suggestion that they might be obligate symbionts or at least have lower saprotrophic capabilities than the strains from the other clusters [8]. The relative phylogenetic positions of the three *Frankia* clusters within the genus has fluctuated, in that different genes used for phylogenetic analysis led to different topologies [7,9]. However a recent study using a concatenation of 54 proteins and other measures shows that Cluster II is basal to the other two clusters [10].

The genomes of three strains of Cluster I (*Frankia alni* ACN14a, QA3 and *Frankia* sp. CcI3) and of four strain of Cluster III (*Frankia* sp. EAN1pec, EUN1f, BCU110501 and BMG5.12) have been published [11,12,13,14,15]). The large genome size variation between 9.3 Mb (EUN1f) and 5.43 Mb (CcI3) has been suggested to reflect differences in saprotrophic potential [11].

In spite of their large host range, Cluster II strains display a remarkably low level of genetic diversity [16] which in an ancient lineage is evocative of a recent evolutionary bottleneck. Such a bottleneck could be associated with previous abundance of certain host plants, followed by a catastrophic event leading to loss or reduction of autonomy of the microsymbiont. Interestingly, one of the host plant genera nodulated by Cluster II strains is *Dryas* (Rosaceae, Rosales), shown to initiate colonization of gravel till following glacial retreat, due to its capacity for biological nitrogen fixation [17], and which once was so abundant that a stadial during an interglacial age was named after it. This genus appears to be losing its symbiotic potential since one of the three known species, the Eurasian *D*. *octopetala* is systematically found devoid of nodules [18]. Two other Cluster II host genera, *Datisca* and *Coriaria* (Cucurbitales), also have a disjunct distribution evocative of taxa threatened by extinction [19,20]. In the case of *Datisca*, one species (*Datisca glomerata*) is found in California and northern Baja California, Mexico, while the other species (*Datisca cannabina*) is found in the east Mediterranean and in the foothills of the Himalayas in Pakistan and northwestern India [21].

The genome of the first representative of Cluster II, *Candidatus* Frankia datiscae Dg1 was sequenced using bacteria isolated from the infected cells of nodules (Dg1; NC_015656.1 (chromosome) and NC_015664.1 (plasmid); [21]). Consistent with the hypothesis that genome size reflects saprotrophic, not symbiotic potential, the genome of Dg1 has, at 5.32 Mb, one of the smallest *Frankia* genome known thus far, suggesting a genomic reduction that could correspond to a reduced saprotrophic lifestyle. At the same time, the difference in genome size between Dg1 and the Cluster I strain CcI3, which can be cultured, is not large. In this study, the *Frankia* genomes that were first published—ACN14a, CcI3 and EAN1pec [11] will be used for comparisons.

The capability to form root nodule symbioses evolved in part by recruiting mechanisms adapted from the evolutionarily older arbuscular mycorrhizal (AM) symbioses [22]. In legume/rhizobia symbioses, flavonoids exuded by plant roots induce expression of bacterial nodulation (*nod*) genes leading to the synthesis of lipo-chito-oligosaccharide (LCO) signal molecules called Nod factors. These LCOs are perceived by plant kinases of the LysM-RLK family and activate a signaling pathway, the common symbiotic pathway, that controls both legume/rhizobia and AM symbioses. Also AM fungi produce LCO signal factors [23], although in their case, there is no evidence to link these factors to host specificity. Results obtained using the only non-legume nodulated by rhizobia, *Parasponia* sp., show that the establishment of an AM symbiosis requires a LysM receptor kinase [24]. The common symbiotic pathway, the known components of which include the receptor kinase SymRK and the calcium- and calmodulin dependent kinase CCaMK [25] is also used for microsymbiont signaling in actinorhizal symbioses, as shown for SymRK in *D*. *glomerata* (Cucurbitales; [26]), for SymRK and CCaMK in *Casuarina glauca* (Fagales; [27,28]) and supported by the presence of homologs of the whole symbiotic cascade in *Alnus glutinosa* and *C*. *glauca* [29] as well as *D*. *glomerata* [30]. Since LCOs or, alternatively, short-chain chitin oligomers (COs) are thought to be involved in signaling *via* the common symbiotic pathway in AM symbioses [23,31], the ancestral symbiosis from which features were recruited for both legume and actinorhizal symbioses, it seems likely that the symbiotic signals produced by *Frankia* strains were also LCO-like or CO-like compounds that are perceived by LysM receptor kinases.

**Fig 1:**
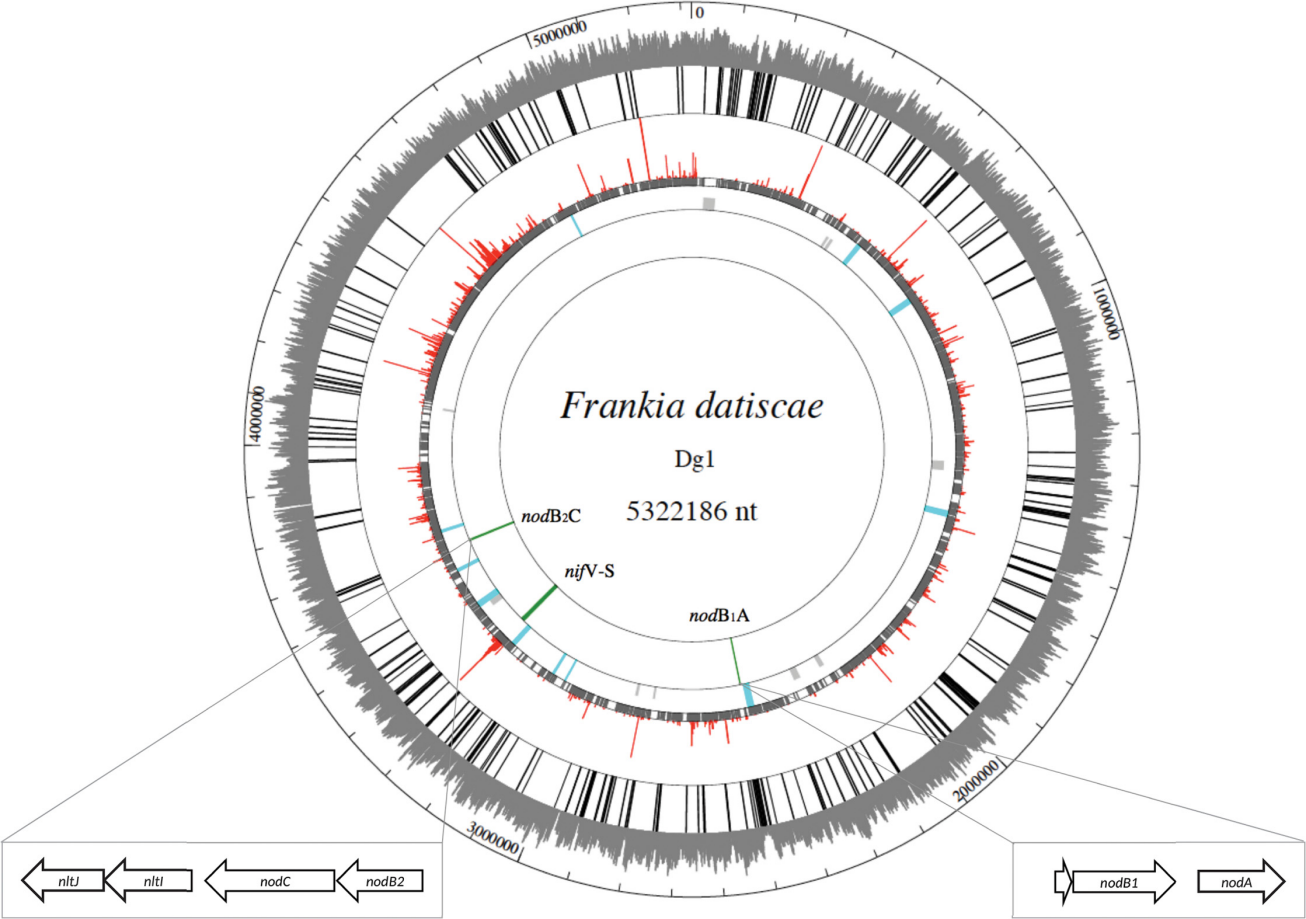
Circular map of the Dg1 genome. The outer circle shows the GC content (above 50%), the next circle shows the positions of IS elements (for detailed positions, see S2 Table). The next circle shows the results of mapping the transcriptome of a closely related cluster II strain on the Dg1 genome: the red peaks represent the genes expressed in the top 5% of expression levels, the grey peaks represent genes represented by at least one read per kb. The next circle shows the operons of genes encoding enzymes involved in the biosynthesis of secondary metabolites (S4 Table): blue color denotes genes which are represented in the symbiotic transcriptome, grey color operons that are not. The last circle shows the positions of the two operons of canonical *nod* genes (*nodA’B1A* and *nodB2C*) and the *nif* gene operon. The *nod* operons of Dg1 are depicted in detail. The *nodA’B1A* operon is at position 2,490,158–2,489,200 of the genome, the *nodB2C* operon at position 3,662,361–3,664,562. The latter is followed by the *nltIJ* genes at position 3,664,716–3,666,622.

However, no signaling substances with the chemical properties of LCOs have been detected to date in *Frankia* culture supernatants [32]. At the genome level, synthesis of the acylated chitin oligomer backbone of Nod factors requires the activity of three specific enzymes, encoded by the so-called “canonical *nod* genes” *nodABC* (NodA—acyl transferase, NodB—chitin deacetylase, NodC—chitin synthase), which form a cluster present in all rhizobia characterized thus far except for one subgroup that nodulates stems of plants of the genus *Aeschynomene* [33]. No homologs of these canonical *nod* genes were found in the genomes of the *Frankia* strains from Clusters I and III [11], aside from genes encoding polysaccharide deacetylases (but with low similarity to the NodB subfamily) and chitin synthases (but not of the NodC subfamily). As a result of whole-genome sequencing, finishing, and annotation [21], we identified homologs of the canonical *nod* genes in the genome of the Cluster II *Frankia* strain, Dg1 (Fig 1) and thus set out to assess their phylogeny and function.

## Materials and Methods

### *Frankia* sp. phylogeny based on 50 housekeeping genes

A set of 50 housekeeping genes was identified in *Candidatus* Frankia datiscae Dg1 and then used in a BLASTP search as the query. The corresponding BLAST searches were restricted to *Frankia alni* ACN14a, *Frankia* sp. Ccl3, *Frankia* sp. EAN1pec, *Acidothermus cellulolyticus* 11B, *Geodermatophilus obscurus* DSM 43160, *Nakamurella multipartita* DSM 44233, *Stackebrandtia nassauensis* DSM 44728 and *Thermobifida fusca* YX. All alignments were created using MUSCLE (**mu**ltiple **s**equence **c**omparison by **l**og- **e**xpectation; [34]) at the EMBL-EBI website. Maximum parsimony analyses were performed using the software package PAUP* version 4.0b10 [35]. All characters were weighted equally and gaps in the alignment were treated as missing. A heuristic search strategy with 10 random replicates, TBR branch-swapping and the MULTREES optimization was used. MAXTREES parameter was set to 10,000 per replicate. Support for branches was evaluated using bootstrap analysis [36] and random sequence addition for 100 replicates, using the same parameters.

### Nod protein sequence phylogenies

NodA, NodB, NodC, NodI and NodJ protein sequences were obtained from GenBank (NCBI) [37] by a multistep BLAST procedure using the protein sequence from Dg1 NodA, NodB, NodC, NodI and NodJ (GenBank accession numbers AEH09514, AEH10396, AEH10398 and AEH10399, respectively) as query. A BLASTP search of the non-redundant protein sequence (nr) database using default parameters (word size = 3, Expect threshold = 10, limiting to 100 target sequences, BLOSUM matrix, and an 11:1 gap cost/extension) generated 100 protein sequences of high similarity. To identify more distantly related potential homologs within the Actinobacteria and Proteobacteria, five iterations of PSI-BLAST were conducted using default parameters (word size = 3, Expect threshold = 10, limiting to 500 target sequences, BLOSUM matrix, and an 11:1 gap cost/extension and a threshold for inclusion of 0.0001). Between 30 and 40 sequences were evaluated for analyses that maximized both sequence and taxonomic diversity. Some NodA sequences from diverse taxa in GenBank were eliminated due to short sequence length and/or origin in a nodule metagenome. For Dg1 NodA (AEH09513) homologs in actinobacteria, a BLASTP search for Actinobacterial sequences with similarity of 1e^-10^ or better in the NCBI and additionally in the JGI databases. Nineteen sequences were found in each database (not including Dg1). From these 38, sequences with query coverage of less than 75% were removed. Also, some of the remaining sequences were identical or a partial sequence of another. In such cases, only the longest sequence was kept. This way, altogether 18 unique actinobacterial sequences of NodA homologs were identified.

All alignments were created using MUSCLE [34] at the EMBL-EBI website, followed by manual adjustment. Selection of the appropriate amino acid substitution model was determined using PROTTEST 2.4 [38] through the server housed at the University of Vigo (http://darwin.uvigo.es/software/prottest2_server.html). Maximum Likelihood analyses for all alignments (NodA, NodB, NodC, NodI, and NodJ) were performed using PhyML 3.0 [39] on the ATGC bioinformatics server. The settings for the NodA run included the JTT model and rate variation sampled from a gamma distribution (JTT+G). The settings for the NodB run included the WAG model, estimated invariant sites, and rate variation sampled from a gamma distribution (WAG+I+G). NodC settings included the LG model, estimated invariant sites, and rate variation sampled from a gamma distribution (LG+I+G). NodI and NodJ settings were LG+G. Support for branches was evaluated using bootstrap analysis [40] and random sequence addition for 100 replicates, using the same parameters.

### Transcriptome analysis

Seedlings of *Datisca glomerata* (Presl.) Baill were grown in a greenhouse (UC Davis) and inoculated with rhizosphere soil from previously-nodulated *Ceanothus* spp. Nodules from two batches of four plants each were snap-frozen in liquid nitrogen at 4 or 6 weeks after inoculation, and pooled by weight. Total nodule RNA was extracted after grinding tissue in liquid nitrogen, using the Plant RNeasy kit (Qiagen, Valencia, CA, USA). Ribosomal RNA was twice subtracted from total RNA with the RiboMinus Plant Kit for RNAseq (Invitrogen, Carlsbad, CA, USA). A cDNA library template was constructed, fragmented, and gel-purified, and the resulting 250 bp average fragments were amplified, according to Illumina (San Diego, CA, USA) protocols. RNA quality and length were checked at each step with a Bioanalyzer (Agilent, Santa Clara, CA, USA). Total RNA was sequenced in a single lane on an Illumina GAIIx system producing ca. 27 million reads (40 bp single reads). A total of 3,712,391 reads mapped to the complete genome of the *Frankia* strain Dg1 (GenBank: CP002801- CP002803) using Bowtie [41]. Ribosomal RNA (16S, 23S and 5S) accounted for 96% of the total mapped reads (3,566,390 reads). The remaining 146,001 reads were filtered for exact duplicate sequences (likely PCR artifacts), sorted and converted to BAM files using the samtools package [42], leaving a final 66,962 reads which were analyzed.

Data visualization and browsing were conducted with the Integrative Genomics Viewer software [43]. Gene expression was calculated as the number of mapped reads per kilobase of gene sequence (rpkb) using custom R scripts [44]. Standard database fields in NCBI were used for gene annotation.

The rpkb data were fitted with a negative binomial distribution (S1 Fig) using the fitdistrplus package in R [45]. Expression quantiles (99%, 95%, 90%, 80%, 70%, and 60%) were computed. Expression of complete or nearly complete metabolic pathways was determined by importing the genome and transcriptome into BioCyc [46], and using Pathway Tools [47]. Pathways, gene and protein annotations, and functional gene clusters were cross-checked in the KEGG Pathway Database supplemented with other sequence databases. A subset of total pathways was selected for further analysis based on high relative expression values, and a minimum of three genes per pathway. Statistically significant overrepresentation of key biosynthetic pathways in the active transcriptome was assessed for genes in each of the quantile bins using a hypergeometric test.

### Quantitative reverse transcription-PCR (qPCR) analysis

For analysis of *nod* gene expression in symbiosis, total RNA was isolated from *D*. *glomerata* nodules after grinding in liquid nitrogen and five passages of 1 min each in a Tissuelyzer (Qiagen), using the RNeasy Plant Mini Kit with on-column DNase digestion (Qiagen). Reverse transcription was performed on 1 μg total RNA from three biological replicates in a final volume of 20 μl, using the TATAA GrandScript cDNA Synthesis Kit (TATAA Biocenter, Gothenburg, Sweden) following the protocol provided by the manufacturer. All qPCR assays contained 1x Maxima SYBR Green qPCR Master Mix (Fermentas, Vilnius, Lithuania), 300 nM of each primer, 2 μl of 10x diluted cDNA in a total reaction volume of 10 μl. qPCR was conducted under the following conditions: 10 min of initial denaturation at 95°C, 40 cycles of 15 s at 95°C, 30 s at 60°C and 30 s at 72°C, followed by steps for dissociation curve generation (15 s at 95°C, 15 s at 60°C and 15 s at 95°C). Primer sequences are given in S1 Table. Assay performance was evaluated with a standard curve. The Eco Real-Time PCR system (Illumina, San Diego, CA, USA) was used for data collection and standard curve generation. PCR products were validated by dissociation curve analysis and agarose gel electrophoresis. Assays were analyzed in triplicate with the standard curve method [48]. PCR efficiency was calculated with the Eco software with data obtained from the exponential phase of each amplification plot. Primers were designed using Beacon Designer software (PREMIER Biosoft, Palo Alto, USA; S1 Table). Gene expression data was normalized against expression of the translation initiation factor gene *IF-3*. Data analysis including data pre-processing and normalization was performed with GenEx (version 5.4.1, MultiD Analyses, Gothenburg, Sweden).

For qPCR analysis of nitrogen assimilation genes, reverse transcription was performed on 1 μg total RNA from three biological replicates in a final volume of 25 μl, using the MMLV-RT Promega M170A kit following the protocol provided by the manufacturer [49]. All qPCR assays contained 1x Maxima SYBR Green qPCR Master Mix (Applied Biosystems), 5 μM of each primer, 1 μl of 10x diluted cDNA in a total reaction volume of 20 μl. qPCR was conducted under the following conditions: 10 min of initial denaturation at 95°C, 40 cycles of 15 s at 95°C, 30 s at 58°C in a Termociclador ABI Prism 7500. Primer sequences for: *asl*, 5’-GAACACCGGCTCCTTGTCCTC-3’ and 5’- CGTCGAGCTGGGTTTCGACTC-3’; for *gogatFD*, 5’ GATGGTGGCGGTGTACTTCT-3’ and 5’- TGCTGAAGGTCATGTCCAAG-5’; for *glnI*, 5’-TTCGCTTCTGTGACCTTCCT-3’ and 5’- GTCGTAGCGTACGTCGTCAA-3’; for *glnII*, 5’-CAGGCCTACGAGAAGTACGC-3’ and 5’- CCTGGTGGAGAAGTTGGTGT-3’; for *argJ*, 5’-GTTCGTCCAGACCGTCAGTT-3’ and 5’- GGTGCAACCTGCTCAAGTG-3’. Primers for the reference gene, 16S rRNA, were 5’-GGGGTCCGTAAGGGTC-3´ and 5´-CCGGGTTTCCCCATTCGG-3´.

### Genome analyses

The Dg1 genome consisted of a chromosome (CP002801) and two plasmids, pFSYMDG01 (CP002802) and pFSYMDG02 (CP002803). Since pFSYMDG01 is represented in the transcriptome while pFSYMDG02 is not, and pFSYMDG02 shows 100% homology with yeast transposons (see e.g., AP012213.1), we can conclude that pFSYMDG02 represents a contamination.

The analysis of the genome sequences with regard to biochemical pathways in Dg1 was performed using Pathway tools [40], MAGE, and IMG/ER. Secondary metabolism (of ACN14a, CcI3 EAN1pec, CcI3, and Dg1) was analyzed using antiSMASH (http://antismash.secondarymetabolites.org; [50]). The core genome between four sequenced *Frankia* strains (ACN14a, EAN1pec, CcI3, and Dg1) was determined using EDGAR (http://edgar.cebitec.uni-bielefeld.de; [51]). The Phyloprofile function of the MAGE platform [52] was used to extract those genes present in the ACN14a, CcI3 and EAN1pec but absent in Dg1 at a sequence similarity of 30% over a length of 80% of the shortest sequence. Palindromic Repeats were analyzed with the palindrome tool from EMBOSS (http://bips.u-strasbg.fr/EMBOSS/) with no mismatches and the following parameters: 1. Repeat units between 8 and 11 bases with up to a 3 base gap. 2. Repeat units between 12 and 19 bases with up to a 7 base gap. 3. Repeat units between 20 and 90 bases with up to a 20 base gap. 4. Repeat units of less than 12 bases must occur at least 10 times in the genome. 5. Repeat units of less than 20 bases must occur twice in the genome. Tandem repeats was analyzed with the MUMmer 3.13 package (http://www.tigr.org/software/mummer/) with the following parameters: Minimum match length = 20 bases. 2. It is assumed that one copy of a tandem repeat in a genome is not very significant unless it is long. Therefore, a genome-wide screen for the repeat used was added. The total number of bases incorporated into repeats for a particular repeat unit must total 50 or more bases.

Pseudogene counts were based on analysis by the IMG (Integrated Microbial Genomes) platform of JGI.

### Finding and identifying repeats

To identify ISs in the investigated organisms, a database of potential ISs was assembled from two sources: First, the program RepeatScout [53] was used to identify all repeated nucleotide sequences with >500 nt length in the investigated organisms. Repeats associated to non-mobile repeated genes, e.g. rRNA, photosynthesis genes and other regular genes present in multiple copies in the genome, were removed. This yielded 173 putative IS elements.

Second, 4512 likely ISs identified in a previous investigation [54] were added to the database. This database included 3377 repeats from the ISfinder site [55], a web repository of known ISs, as well as 1135 repeats from cyanobacteria, *Frankia* genomes available in 2012 and other known symbiotic bacteria. The repeats found by RepeatScout were named with an abbreviation of the name of the originating organism, the letter ‘R’ and a number, e.*g*. ‘Npun_R_21’ is a repeat from the organism *N*. *punctiforme*, the twenty-first found by RepeatScout. The ISs from ISfinder retained their original names, all starting with ‘IS’. ISfinder and RepeatScout sequences were collected into a single Fasta file. Redundancies, generated when ISfinder sequences were also detected by RepeatScout, were removed, with the ISfinder name taking precedence. Each of the collected nucleotide sequences was used as queries in a BlastN search against each of the genomes, one at a time. All areas of the genome that received hits with a BlastN expect value <1e^-6^ were collected. Often several queries scored hits on the same region of a genome, together making up a “footprint” in the genome.

In the next step, each footprint was analysed to determine what repeats it consisted of. This was performed by using the footprints as queries against the database of repeats. The repeat sequence that received the highest score with the footprint as query was chosen as the most likely IS to occupy the footprint. In some cases, the best scoring repeat didn’t cover the entire footprint. The search was then repeated with the remaining footprint as query. The process was repeated until all parts of the footprints had been identified. The whole process yielded a GenBank file for each of the investigated genomes, where the verified and putative ISs are described with position, kind and quality of identification (S2 Table).

### Analysis of nodule occupancy

Seedlings of *Datisca glomerata* were grown and nodulated under controlled conditions in two locations: at the Department of Plant Ecology, Stockholm University (SU) and the Department of Plant Sciences, University of California, Davis (UCD). The inoculum source for the SU plants was a ground nodule suspension containing *Frankia* originally from Pakistan [56]; the inoculum source for the UCD plants was soil from the rhizosphere of nodulated *Ceanothus griseus* in California. DNA was extracted from several nodules from a combination of at least four plants, using the QIAGEN DNeasy plant mini kit following the manufacturer’s instructions. Two replicates from each source were amplified by PCR using universal primers 27F (5'-AGAGTTTGATCCTGGCTCAG-3') and 338R (5'-TGCTGCCTCCCGTAGGAGT-3') and sequenced on a GS FLX + Sequencing System (454 Life Science/Roche), Kansas State University.

92,880 sequencing reads from the 454 were initially generated from the four DNA samples, and after filtering for barcodes, adapters, length, and quality, 54,361 remained. Sequences were trimmed and denoised [57] to avoid artificial inflation of rare diversity from homopolymer read errors, and processed in QIIME 1.5.0 [58] to assign operational taxonomic units (OTUs) using a 97% identity threshold, assign taxonomy to OTUs (identifications of >80% confidence), and calculate abundances of OTUs in samples. Finally, using BLAST [59] and QIIME, reads were curated to remove sequences from mitochondria or chloroplast, and to merge OTUs not reaching 0.2% abundance in any sample into a single category. Finally, 2,833 and 4,209 bacterial reads from the two SU nodule samples (with mean 3521 and standard deviation 973), and 1,994 and 4,822 bacterial reads from the two UCD nodule samples (with a mean of 3408 and a standard deviation of 2000) were included in analysis.

Phylogenetic analysis was conducted on five v1-2 region of 16S rDNA sequences (S3 Table) of *Frankia* OTUs together with seven v1-2 region sequences and two v2 region sequences (Cn endophyte; Dc endophyte) of Cluster II *Frankia* strains and three v1-2 region sequences of Cluster I or III *Frankia* strains available in GenBank [37]; S3 Table). Among the added Cluster II *Frankia* sequences, three originated in Pakistan (Dg1, Cn endophyte, Dc endophyte), one originated in New Zealand (FE37), and all others originated in North America. Sequence alignment was done using MAFFT [60]; the length of the aligned sequences was 321bp. A neighbor-joining tree was constructed based on a pairwise distance matrix of percent nucleotide difference with 1000 boot strap replicates using PAUP* version 4.0b10 [61]. All positions were weighted equally and all gaps were treated as missing data.

### *Rhizobium leguminosarum nodC* complementation

As a positive control, a cluster comprising *nodD*, *nodA*, *nodB* and *nodC* genes was amplified using DNA from *Rhizobium leguminosarum* strain A34 [62], Pfu (Promega, Charbonnière-Les-Bains, France) polymerase and primers F7800 5’-GTGCTGCATGCGTGCCGCTTACGACGTACAACTT-3’ and F7799 5’-TATAGGAATTCCTGCAGTGACGCGTTCATCACT-3’. PCR conditions were: 2 min at 95°C followed by 35 cycles at 95°C 1 min; 63°C 30 s; 72°C 7 min, followed by 72°C for 5 min. The PCR fragment was cloned into a pGEM-T easy vector (Promega) and the DNA insert verified by sequencing. The plasmid containing a PCR insert was then digested using restrictions enzymes *Sph*I and *Eco*R1 and ligated into a *Sph*I /*Eco*RI digested pBBR1 MCS5 vector [63]. It was then introduced first into *E*. *coli* DH10B (Invitrogen) and then into *R*. *leguminosarum* A56 (*nodC128*::Tn*5*) [62] by electroporation [64].

The *nodC* gene from Dg1 (*nodC_Dg1*) was amplified using primers F7308 5’-ACCAGGATCCTCACGATGACAGCGGGG-3’ and F7350 5’-AAACCCATATGTCGACCGCGGTGAG-3’ and Taq (Invitrogen, Villebon sur Yvette, France) in 1 x Taq buffer containing 5% DMSO (vol/vol). PCR conditions were: 5 min at 95°C followed by 40 cycles at 94°C, 45 s; 59°C, 45 s; 72°C, 45 s, followed by 72°C for 10 min. The PCR fragment was cloned into the pGEM vector, which was transformed into *E coli* DH10B and confirmed by sequencing. Plasmids containing the *nodC_Dg1* gene were used as DNA template for further amplification and cloning.

The *R*. *leguminosarum nodC* gene replacement by *Candidatus* Frankia datiscae Dg1 *nodC_Dg1* gene was done in three PCR steps. *nodDnodAB* genes from *R*. *leguminosarum* A56 were amplified using primers F8029 5’-CATGGTTTCTCGTTTGTCCAGTGTTTC-3’ and F7800 5’-GTGCTGCATGC GTGCCGCTTACGACGTACAACTT-3’ using Pfu polymerase. PCR conditions were: 2 min at 95°C followed by 35 cycles at 95°C, 1 min; 60°C, 30 s; 72°C, 5 min, followed by 72°C for 10 min. The *nodC_Dg1* gene from *Frankia Dg1* was amplified using primers F8030 3’-GAAACACTGGACAAACGAGAAACCATGTCGACCGCGGTGAG-3’ and F8210 5’-ACCAGGAATTCTCACGATGACAGCGGGG-3’ and Taq polymerase (Invitrogen). One μL of each PCR product were mixed and amplified using primers F7800, F8210 and Taq polymerase (Invitrogen). PCR conditions were: 4 min at 95°C followed by 35 cycles at 95°C, 1 min; 51°C, 30 s; 72°C, 7 min, followed by 72°C for 10 min. A 3.7 kb DNA fragment containing fusion *nodDABC*_Dg1 was eluted from the agarose gel using a micro elute column (Qiagen, Courtaboeuf, France), ligated into the pGEM-T easy vector (Promega, Madison, WI, USA) and transformed into *E*. *coli* DH10B by electroporation. Plasmid DNA isolation were done from several colonies. The correct gene fusion was confirmed by sequencing. The DNA insert was then cloned in the *Eco*RI and *Sph*I sites of the pBBR1 MCS5 vector and transformed into *E*. *coli* DH5α. Transfer to the *R*. *leguminosarum* A56 *nodC* mutant strain was performed by tripartite conjugation using *E*. *coli* containing the pRK2013 plasmid. The construct in *R*. *leguminosarum* A56 with the *Frankia* Dg1 *nodC_Dg1* gene was verified after conjugation by amplification of *nodC-Dg1* gene and of the 16S rDNA and confirmed by sequencing, plasmid DNA preparation and enzyme restriction analysis.

Seeds of *Pisum sativum* (Wisconsin perfection) were obtained from INRA Dijon (France), surface sterilized with ethanol 70% (vol/vol) for 1 minute, rinsed and treated for 12 minutes in NaClO 10% (w/vol), rinsed and then germinated on agar. The seedlings were then transferred into 500 ml glass flasks containing 250 ml of nitrogen-free FP medium solution [65] gelified with 0.5% agar and capped with cotton wool. When the seedlings were 2 weeks old, they were inoculated with wild-type *R*. *leguminosarum* A34 (WT), *R*. *leguminosarum* A56 (*nodC128*::Tn*5*) with recombinant *nodDnodABC*, or with *R*. *leguminosarum* A56 with recombinant *nodDnodABC_Dg1* with *nodC* from Dg1, or not inoculated, grown for 72 h at 30°C on TY agar with 0.6 mM Calcium Chloride [66] and resuspended in distilled water. The plants were grown in the greenhouse at a 21°C/16°C day/night cycle. The effects of the rhizobia on the plant roots was followed under a stereomicroscope. After infection, the roots were observed twice per week over a period of two months, looking for root hair deformation and nodule formation, on two separate sets of sextuplicates for all genetic constructs. After 31 days, photographs were taken of deformed root hairs. First, plants were observed under a Leica MZ8 stereomicroscope using lateral illumination to avoid light reflections. For photography, a Zeiss Axioskop was used with an Axiocam MRC5 camera, and the root systems with agar were removed from the glass flasks.

## Results

### Does the genome of Dg1 show auxotrophies or symptoms of reduction typical of obligate symbionts?

The genome of Dg1 has a coding density of 78% which is slightly lower than those of the other sequenced *Frankia* strains. The core genome (at a threshold level of 30% amino acid identity over 80% of the length of the shortest sequence) of the four studied genomes of *Frankia* strains consisted of 1616 coding sequences (CDSs; S2 Fig). The CcI3 genome displayed the highest similarity to the core genome, with only 30% (1351 CDSs) of its CDSs not shared with the other *Frankia* strains. In contrast, 39% (1598 CDSs) of the CDSs of Dg1, 45% (3051 CDSs) of ACN14a and 50% (3606 CDSs) of EAN1pec were not shared with any other *Frankia* strain.

A comparison of the metabolic pathways encoded in the genomes of the sequenced *Frankia* strains using MicroCyc (http://www.genoscope.cns.fr/agc/microscope/metabolism/microcyc.php) and Phyloprofile (https://www.genoscope.cns.fr/agc/website/spip.php?article600) showed several putative auxotrophies in Dg1 that could impede saprotrophic growth. However, since similar auxotrophies were found in the genomes of cultured *Frankia* strains, the only safe conclusion to be drawn is that not all actinobacterial versions of common metabolic enzymes are characterized yet. For this reason, it is also difficult to compare the saprotrophic potential—i.e., capability of growing on different nutrient sources—of different *Frankia* strains based on their genome sequences.

In comparison with CcI3, EAN1pec and ACN14a which have a high capacity for the production of secondary metabolites [67], Dg1 had a lower potential for the biosynthesis of polyketides, non-ribosomal peptide synthases and terpenoids (S4 Table). Several operons for lantibiotic biosynthesis that are present in CcI3, EAN1pec and ACN14a are absent in Dg1, and genes for bacteriocins present in ACN14a and EAN1pec are not present in Dg1 (S4 Table). The *gvp* operon, responsible for the synthesis of gas vesicle proteins that are supposed to maintain bacteria at the top of the water table in the soil [68], which is present partly in CcI3, and completely in ACN14a and EAN1pec, is missing in Dg1. These features indicate that Dg1 possesses a comparatively low potential for the biosynthesis of siderophores for iron scavenging, and of compounds used against competitors in the soil habitat.

In bacterial symbionts, proliferation of the mobilome is a key feature of early stages of genome reduction [69,70,71]. The mobilome encompasses mobile genetic elements—genetic units that can move within a genome or from cell to cell. Intracellular mobile genetic elements include transposons and insertion sequenes (ISs). The latter are defined as genomic sequences of mobile DNA, typically 800–1300 bp in length, that encode the enzyme transposase. E.g., clusters of ISs have been shown to correlate with areas of gene loss and genomic recombination in *Frankia* strains [72]. In this context, it is interesting that a larger number of transposases was observed in Dg1 compared with the other studied *Frankia* genomes (S5 Table). To further our understanding on genome reduction in *Frankia* strains, we examined the identity and distribution, as well as length distribution, of IS elements in the *Frankia* genomes published to date. We defined a particular DNA sequence encoding a transposase as an IS when it was present in multiple copies in a genome, taking into account that IS elements may have accumulated mutations leading to differences in sequence and length. The results showed that Dg1 clearly contains the highest relative amount of IS elements, followed by the *Elaeagnus*-infective strain EAN1pec and the *Casuarina*-infective strain CcI3 (S3A Fig). The latter, like Dg1, has a small genome which has been attributed to IS-mediated reductive evolution [11].

A study of the length distribution of the IS elements in each strain showed that only in Dg1, full length IS elements dominated, while in all other strains examined, full length IS elements represented a minority among the IS elements (in CcI3 and EAN1pec, they made up less than 30%; S3B Fig). The distribution of IS elements in the genome is depicted in Fig 1 and S2 Table.

Many degrading genomes display an elevated level of pseudogenes [73]. The genome of Dg1 contains 325 pseudogenes, 7.01% of the total number of genes. In comparison, CcI3 has 50 pseudogenes (1%), ACN14a 12 (0.18%), and EAN1pec has 128 (1.78%; S5 Table). As an extreme, 31.2% of the CDSs in the obligate symbiont *Nostoc azollae* are pseudogenes [74].

### Evolution of symbiotic *Frankia* strains: which Cluster is basal?

In the framework of a re-examination of the phylogeny of actinobacteria using 54 house-keeping genes from 100 sequenced genomes, Sen et al. [10] determined *Frankia* Cluster II (represented by Dg1) as basal *Frankia* clade, followed by the non-symbiotic Cluster IV, and then the symbiotic Cluster III and finally Cluster I as the most derived. This is consistent with the evolution of nitrogen-fixing symbioses as examined by Werner et al. [4] in that the oldest symbioses—of Cucurbitales, since the evolution of the symbiotic capabilities must have preceded speciation of *Coriaria* sp. [20]—are those involving Cluster II strains. However, given the wide range of the analysis, a new, more focussed analysis seemed in order to confirm the basal position of Cluster II among the symbiotic *Frankia* strains.

The phylogenies of 50 different housekeeping genes were analyzed for the first four published *Frankia* genomes, using *Geodermatophilus obscurus* G20 (NC_013757 [75]), *Acidothermus cellulolyticus* 11B (NC_008578 [76]), *Stackebrandtia nassauensis* (NC_013947 [77]) and *Nakamurella multipartita* Y-104 (NC_013235 [78]) as outgroups. The results are depicted in S4 Fig and summarized in Table 1. For 42 out of 50 genes, Dg1 was basal to the other *Frankia* strains, while EAN1pec (Cluster III) and ACN14a/CcI3 (Cluster I) formed derived sister groups. Eight genes (*dapB*, dihydrodipicolinate reductase; *dnaA*, chromosomal replication initiation protein; *folC*, dihydrofolate synthase; *glpX*, fructose 1,6-bisphosphatase II; *idi*, isopentenyldiphosphate isomerase; *ispA*, intracellular septation protein; *murC*, UDP-N-acetylmuramate-L-alanine ligase; *rplA*, ribosomal protein L1) showed different phylogenies; for four of them (*dnaA*, *folC*, *glpX*, *idi*) Dg1/EAN1pec (Clusters II/III) and ACN14a/CcI3 (Cluster I) appeared as sister groups, and for two of them (*murC*, *rplA*), EAN1pec (Cluster III) was basal. In summary, the results predominantly show that Cluster II is the basal group of symbiotic *Frankia* strains. Those genes that yielded a topology with Dg1 at a derived position were shorter (with a mean length of 464 aa, 1392 bp) than those (with a mean length of 521 aa, 1563 bp) that yielded a topology with Dg1 at the base of the *Frankia* radiation. The smaller length is correlated with an *a priori* smaller phylogenetic weight.

**Table 1:** *Frankia* phylogeny was analysed using 50 housekeeping genes and the four published genomes of strains ACN14a and CcI3 (Cluster I), Dg1 (Cluster II) and EAN1pec (Cluster III).

Gene	DNA or Protein	Length of Alignment	Score of best tree found	# of most pars. Trees	*Frankia* Dg1 first branching?	*Frankia* mono-phyletic?
*16S 23S*	DNA	4944	4505	1	Y	Y
*aceE*	Protein	500	988	1	Y	Y
*aroK*	Protein	256	513	5	Y	Y
*atpA*	Protein	556	705	1	Y	Y
*bioA*	Protein	486	1138	1	Y	Y
*bioB*	Protein	458	966	1	Y	Y
*clpP*	Protein	237	366	1	Y	Y
*dapA*	Protein	356	627	1	Y	Y
*dapB*	Protein	278	449	1	N	Y
*dnaA*	Protein	466	574	1	n	Y
*dnaB*	Protein	547	629	1	Y	Y
*dxr*	Protein	460	686	1	Y	Y
*dxs*	Protein	660	965	1	Y	Y
*eno*	Protein	465	491	3	Y	Y
*folC*	Protein	525	1153	1	n	Y
*ftsZ*	Protein	590	784	1	Y	Y
*fusA*	Protein	705	735	1	Y	Y
*glmU*	Protein	540	1126	1	Y	Y
*glpX*	Protein	375	517	4	n	Y
*gltA*	Protein	489	1009	1	Y	Y
*gyrA*	Protein	850	970	1	Y	Y
*htpX*	Protein	338	604	1	Y	Y
*idI*	Protein	236	572	1	n	n
*ispA*	Protein	359	678	1	Y	Y
*ksgA*	Protein	339	591	2	Y	Y
*lipA*	Protein	360	447	2	Y	n
*lytB*	Protein	355	472	2	Y	Y
*metK*	Protein	418	380	1	Y	Y
*mfd*	Protein	780	1014	1	Y	Y
*mraY*	Protein	368	611	2	Y	Y
*murA*	Protein	516	1004	1	Y	Y
*murC*	Protein	668	1320	1	n	Y
*murG*	Protein	396	767	1	n	Y
*nth*	Protein	289	476	2	Y	Y
*pgk*	Protein	420	639	1	Y	Y
*recA*	Protein	355	261	3	Y	Y
*ribA*	Protein	480	627	1	Y	Y
*ribE*	Protein	175	288	1	Y	Y
*ribF*	Protein	355	758	1	Y	Y
*ribH*	Protein	175	289	1	Y	Y
*rplA*	Protein	260	262	1	n	Y
*rplB*	Protein	279	184	3	Y	Y
*rplC*	Protein	245	291	1	Y	Y
*rplD*	Protein	306	458	1	Y	Y
*rplE*	Protein	206	205	3	Y	Y
*rplF*	Protein	179	227	6	Y	n
*rpoB*	Protein	1209	948	1	Y	Y
*shc*	Protein	751	923	1	Y	n
*tpiA*	Protein	288	469	1	Y	Y
*trpE*	Protein	450	1367	1	Y	Y
*uppS*	Protein	293	585	2	Y	Y

*A*. *cellulolyticus* 11B, *S*. *nassauensis* DSM 44728, *G*. *obscurus* DSM 43160, *N*. *multipartita* DSM 44233, and *T*. *fusca* YX were used as outgroups. The statistical evaluation is given in S4 Fig.

### Dg1 contains two operons of canonical *nod* genes

**Fig 2:**
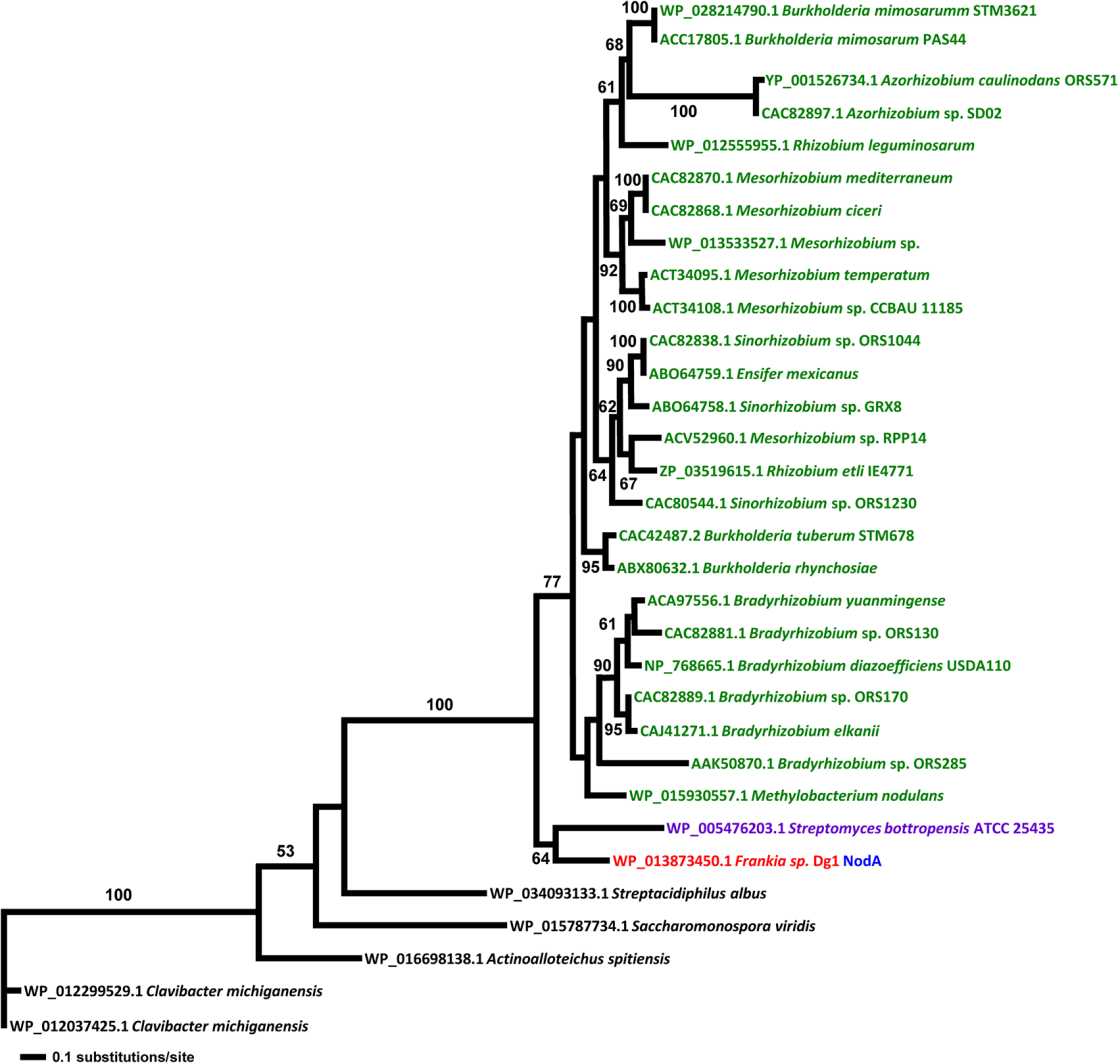
Maximum Likelihood trees of NodA proteins. All rhizobial sequences are given in green, all sequences from Dg1 are given in red, sequences from *Streptomyces bottropensis* are given in purple. The Dg1 NodA sequence is indicated in blue. All sequences used for the phylogenetic analysis are given in S6 Table.

**Fig 3:**
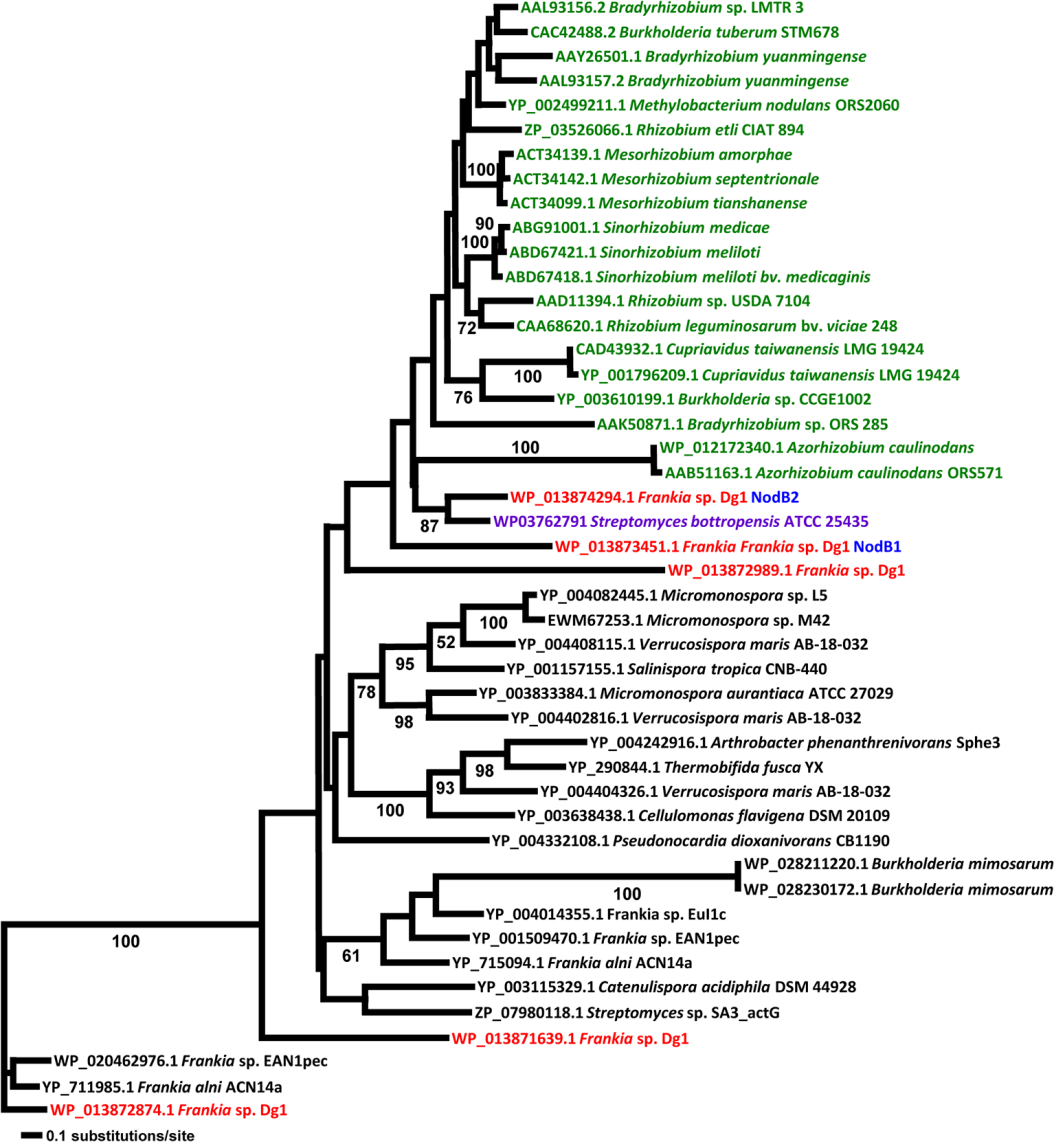
Maximum Likelihood trees NodB proteins. All rhizobial sequences are given in green, all sequences from Dg1 are given in red, sequences from *Streptomyces bottropensis* are given in purple. Dg1 NodB1 and NodB2 sequences are indicated in blue. All sequences used for the phylogenetic analysis are given in S6 Table.

**Fig 4:**
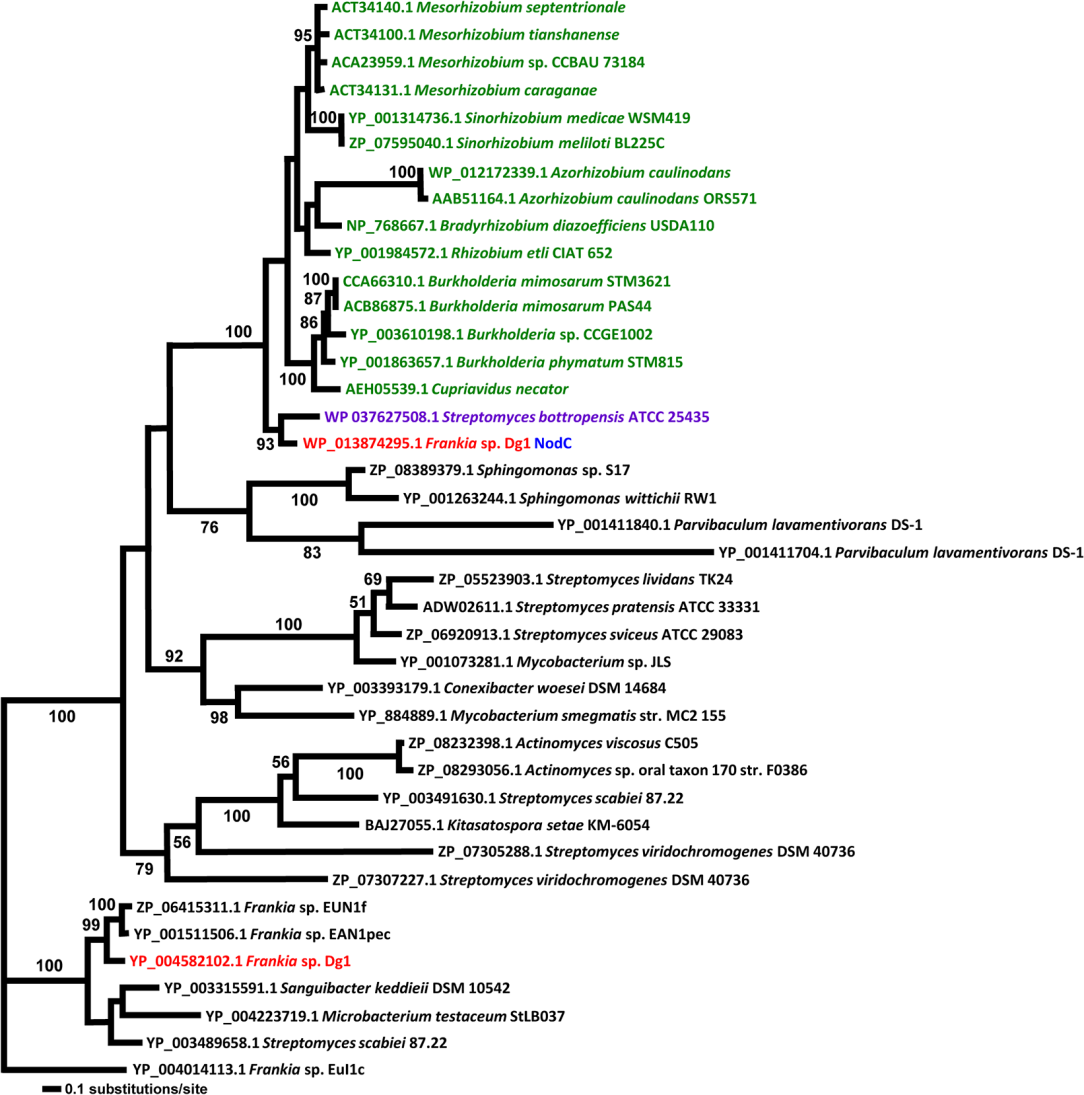
Maximum Likelihood trees of NodC proteins. All rhizobial sequences are given in green, all sequences from Dg1 are given in red, sequences from *Streptomyces bottropensis* are given in purple. The Dg1 NodC sequence is indicated in blue. All sequences used for the phylogenetic analysis are given in S6 Table.

Two operons of homologs of the canonical *nod* genes were found in the Dg1 genome: 1) an operon containing *nodA’B1A*, coding for: NodA’, GenBank accession number AEH09515 (a truncated NodA, only 36 amino acids); NodB1, AEH09514; and a full-length NodA, AEH09513; and 2) a *nodB2CIJ* operon, coding for NodB2, AEH10396; and NodC, AEH10397 (Fig 1). The proteins encoded by these genes display very high amino acid sequence similarity to the canonical *nodABC* genes (Figs 2, 3 and 4; S5 Fig; S6 Table), and their arrangement in the Dg1 chromosome suggests that they are expressed as operons, similar to their counterparts in rhizobia. Based on these findings of amino acid sequence similarity and synteny, they are referred to as *nod* genes throughout this manuscript. The structure of the *nodA’B1A* operon indicates that it is the result of at least two transposition events, though it is not clear whether *nodA* inserted into a functional *nodAB1C* operon from which *nodC* and the largest part of the original *nodA* gene were subsequently lost, or whether the operon was truncated before the insertion.

The NodA proteins belong to a family of acyl transferases previously only described in rhizobia; however, several NodA homologs are also present in a diverse group of Actinobacteria that includes Dg1 (Fig 2; S6 Table), phylogenetically distant from the rhizobia. While these actinobacterial genera with NodA homologs span across the whole phylum [10], only the nodulating taxa within the Proteobacteria have NodA homologs. Furthermore, as shown by the longer branch lengths (Fig 2), the NodA sequences in actinobacteria are more diverse than those of α- or β-Proteobacteria.

In order to ensure that all potential gene familes were represented in the NodA tree, a set of more dissimilar potential NodA sequences with lower e-values in BLAST (two sequences per genus within all proteobacteria) was analyzed by maximum likelihood. In this analysis, even the sequences with low similarities still were nested within the rhizobial clade (data not shown). Thus, no NodA paralogs were found in rhizobia.

In the NodB protein phylogeny (Fig 3), the Dg1 NodB1 is at the base of the rhizobial NodB clade with Dg1 NodB2 at a slightly more derived position, following the insertion of a pair of sequences from *Azorhizobium caulinodans* ORS571 [79]. In the NodC protein phylogeny, NodC is basal to all the rhizobial NodC proteins with strong support. However, sister to this clade is the clade of *Sphingomonas* and *Parvibaculum*, non-nodulating Proteobacteria (Fig 4).

Particularly notable across all the trees are the positions of the NodABC homologs from *Streptomyces bottropensis*. Sequences of *S*. *bottropensis* were found as sister to Dg1 sequences in the NodA, NodB, and NodC trees (Figs 2, 3 and 4). Moreover, although *S*. *bottropensis* so far has not been known to induce nodules, it is the only actinobacterial taxon beside Dg1 that has homologs of all three canonical *nod* genes *nodABC*. Intriguingly, the *nodA-nodB-nodC* genes of *S*. *bottropensis* are present in a single operon. This positioning could suggest that the common ancestor of Dg1 and *S*. *bottropensis* specifically among the actinobacteria was the source of the rhizobial canonical *nod* genes.

Directly downstream from the *nodB2C* operon an ABC transporter operon with significant amino acid sequence similarity (>45%) to rhizobial *nodIJ* is found (encoding AEH10398 and AEH10399). NodJ represents an ABC transporter and NodI its ATPase subunit. It is interesting to note that the position of this operon with respect to *nodB2C* is identical to the position of the *nodIJ* genes in the canonical *nod* gene operon of rhizobia (*nodABCIJ*; [80]). However, the Dg1 genes with homology to *nodIJ* located near *nodB2C* are expressed in symbiosis at very low levels, if at all (see below, transcriptome); therefore, we have termed the genes *nltIJ* for “*nod*-linked transporters I and J”. Intriguingly, in *S*. *bottropensis* the homologs of the rhizobial canonical *nod* genes are present in a *nodBCnltIJnodA* operon, and the NltI/NltJ protein encoded in this operon show high amino acid similarity with Dg1 NltI/NltJ. Our phylogenetic analysis showed that the actinobacterial NltI and NltJ proteins encoded by *nodBC*-linked genes—as opposed to other ABC transporter/ATPase pairs in the same genomes—have very low amino acid similarity with rhizobial NodI/NodJ proteins, obviously coming from a different ABC transporter/ATPase lineage (S6 Fig).

The Dg1 genome has an overall GC content of 70.04%, and the GC contents of the *nod* genes, most strikingly *nodA*, are clearly below that (*nodA*, 58.4%; *nodB1*, 65.2%; *nodB2*, 66.8%; *nodC;* 64.5%). This pattern is also true for the ABC transporter operon linked to *nodB2C* (*nltI*, 65.4%; *nltJ*, 62.4%). Rapid gene evolution is known to be accompanied by AT enrichment, so this may be one explanation for the lower %GC in this gene region [81]. A similar pattern of low %GC of the *nod* genes relative to the genome is found in rhizobia [82], and in the *nodA* homolog of *S*. *bottropensis* (S6 Table). By contrast, the %GC of the more distant *nodA* homologs within the actinobacteria is higher than the %GC of the respective genomes (S6 Table), implying a relatively slow rate of gene evolution [83]. Alternately, another explanation for the lower GC% of the *nod* operons could be their position at the replication terminus, as has been shown to occur in several bacterial genomes [83].

### Both Dg1 *nod* operons are expressed in symbiosis

The expression of the two *nod* operons in Dg1 in nodules of *D*. *glomerata* was examined by quantitative RT-PCR, using the Dg1 translation initiation factor gene *IF-3* (GenBank accession number AEH10595) as constitutive control (see [84]). Transcription of *nodA*, *nodB1*, *nodB2* and *nodC* in nodules (Table 2) was confirmed through transcriptome analysis (see below).

**Table 2:** Expression of the Dg1 genes *nodA*, *nodB1*, *nodB2*, and *nodC* in *D*. *glomerata* nodules given in relative units.

Gene	Expression
***nodA***	64.72 ± 23.29
***nodB1***	2.23 ± 1.55
***nodB2***	1.69 ± 0.45
***nodC***	5.62 ± 2.20

The expression values represent means ± SD (n = 3). The expression of *nodA* is significantly higher than those of *nodB1*, *nodB2* or *nodC*. (One-Way ANOVA with Tukey's multiple comparison test, p ≤ 0.05)

### Dg1 *nodC* can partially complement a *Rhizobium leguminosarum nodC* mutant strain

**Fig 5:**
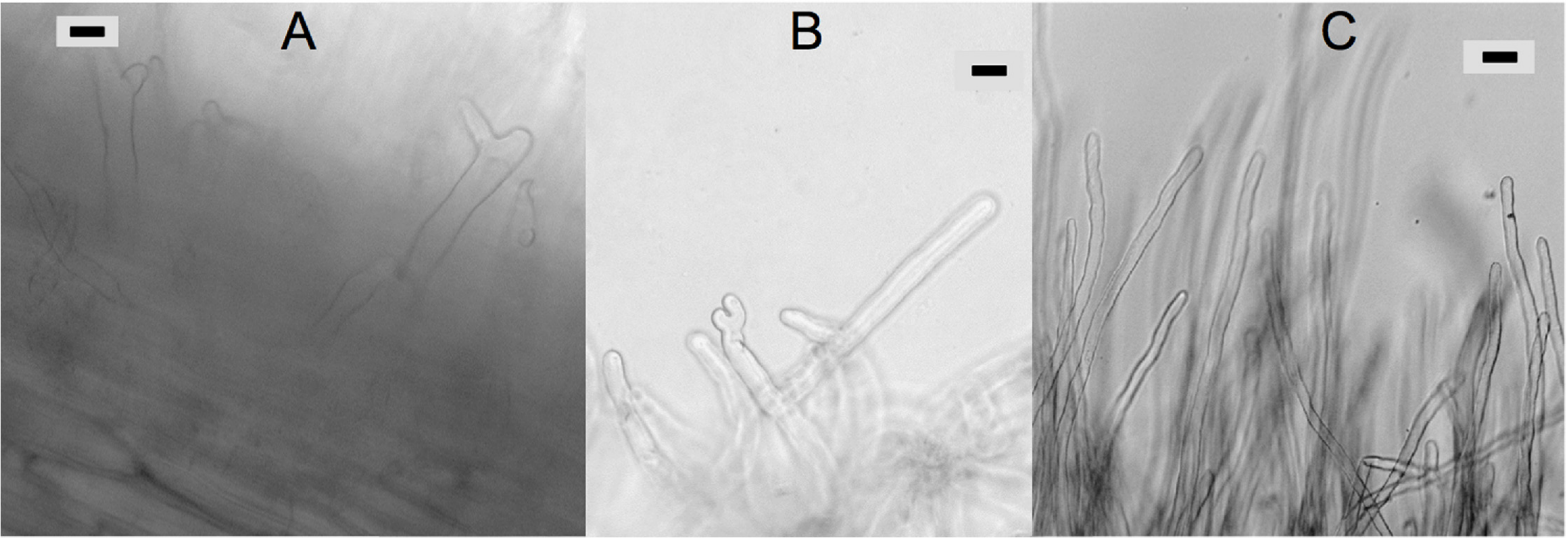
Analysis of the ability of *Frankia* Dg1 *nodC* (*nodC_Dg1*) to complement a rhizobial *nodC* mutant. Roots of *P*. *sativum* show root hair deformation 31 days after inoculation with **(A)** *R*. *leguminosarum* A56 *nodC*::Tn*5* mutant complemented by *Frankia* Dg1 *nodC_Dg1* and after inoculation with **(B)** wild type *R*. *leguminosarum* A34. **(C)** Roots inoculated with the original *R*. *leguminosarum* A56 *nodC*::Tn*5* mutant do not show root hair deformation. Root hair deformation was more pronounced after the infection with wild type rhizobium (B) or with the *nodC*::Tn*5* mutant complemented by the homologous *nodDnodABC* operon (data not shown), than with the strain complemented by *Frankia* Dg1 *nodC_Dg1* (A). Size bars indicate 10 μm.

The *R*. *leguminosarum* A34 (wt), the A56 *nodC* mutant (*nodC128*::Tn*5*; [62]) can be complemented by the homologous *nodC_Dg1* gene. When the rhizobial *nodC* gene in the *nodABC* operon was replaced by *Frankia* Dg1 (*nodC_*Dg1), the ability of the strain to induce root hair deformation on pea was restored, but the ability to nodulate was not (Fig 5). While the restoration of root hair deformation shows that Dg1 NodC_Dg1 does complement *R*. *leguminosarum* A56 *nodC* mutant, the lack of restoration of nodulation indicates that the either activity of NodC_Dg1 in *R*. *leguminosarum* A56 is too low to provide enough Nod factors for this process or that there is incompatibility with the other Nod proteins, formation of an oligomer of a length different from the optimal, etc. Nevertheless, this result shows that Dg1 NodC can fulfill the function of a chitin synthase in Nod factor biosynthesis.

### Transcriptome analysis

The total transcriptome of *Frankia* in young nodules of *D*. *glomerata* encompasses genes expressed in an indeterminate developmental pattern that would correspond to and encompass the infection and nitrogen fixation zones described by Pawlowski and Demchenko [85]. Thus, the calibration of relative transcript abundance for specialized processes such as Nod factor biosynthesis or nitrogen assimilation depends on a spatio-temporally heterogeneous source, and intact biosynthetic pathways are a stronger metric for interpreting metabolic expression patterns than single-gene scores.

All the biosynthetic pathways listed in Table 3 were statistically overrepresented (p < 0.05) in the 80th quantile (top 20% most abundant) of rpkb-ranked transcriptome data. Genes coding for the pathway for nitrogenase biosynthesis (*nifHDK*, *nifV*, *nifENXWZBS*) were the most highly expressed, with significant overrepresentation in the top 1% (99^th^ quantile (p = 1.2e^-5^). The TCA pathway, iron-sulfur cluster biosynthesis (SUF pathway), and NADH quinone oxidoreductase synthesis were significantly enriched in the top 5% (p < 0.02). In the top 10% (90^th^ quantile), gene clusters coding for cytochrome c oxidase biosynthesis, isoprene biosynthesis, and branched-chain amino acids pathway (ILV pathway) were enriched significantly (p< 0.05). Nod factor biosynthesis (*nodA´B1A and nodB2C* operons) and the arginine biosynthesis complete pathway were significant in the 80^th^ quantile (p<0.03). The ABC transporter operon downstream to the *nodB2C* operon (*nltIJ*) was expressed at low confidence, in the 70th and 60th quantile, respectively (not shown in table).The expression of the genes for enzymes involved in nitrogen assimilation and amino acid biosynthesis was confirmed by qPCR (Table 4).

**Table 3:** Statistically significant overrepresentation of key biosynthetic pathways in the active nodule transcriptome.

Pathway/product	# genes in pathway	# genes expressed >99th quantile (/69 genes)	p-hyper	# genes expressed >95th quantile (/196 genes)	p-hyper	# genes expressed >90th quantile (/334 genes)	p-hyper	# genes expressed >80th quantile (/680 genes)	p-hyper	# genes expressed >70th quantile (/1146 genes)	p-hyper	# genes expressed >60th quantile (/1805 genes)	p-hyper
Nitrogenase	10	4	1.2E-05	7	4.0E-08	8	4.9E-08	10	9.6E-09	10	1.8E-06	10	1.7E-04
TCA pathway	12	1	1.5E-01	5	1.2E-04	10	4.2E-10	12	2.4E-10	12	1.3E-07	12	3.1E-05
FeS cluster assembly (SUF)	6	0	9.3E-02	3	1.7E-03	5	1.6E-05	6	1.6E-05	6	3.6E-04	6	5.6E-03
NADH quinone reductase	13	0	1.9E-01	3	1.9E-02	8	1.1E-06	11	8.6E-08	12	1.3E-06	13	1.3E-05
Isoprene pathway (MEP)	9	1	1.4E-01	1	3.4E-01	4	3.3E-03	6	8.5E-04	7	2.1E-03	8	5.5E-03
Nod factor synthesis (*nodABC*)	5	0	7.8E-02	0	2.1E-01	1	3.3E-01	3	3.1E-02	5	1.4E-03	5	1.3E-02
Arginine pathway	7	0	1.1E-01	0	2.8E-01	2	9.8E-02	6	9.5E-05	7	9.7E-05	7	2.3E-03
ILV pathway	11	0	1.6E-01	1	4.0E-01	3	4.8E-02	9	2.5E-06	10	1.5E-05	11	7.3E-05

TCA is the tricarboxylic acid cycle (respiration), FeS clusters are iron-sulfer clusters (used in nitrogenase, hydrogenase etc.), MEP is the non-mevalonate pathway or 2-C-methyl-D-erythritol 4-phosphate pathway (hopanoids), ILV pathway is the isoleucine, leucine and valine pathway (biosynthesis of branched chain amino acids).

**Table 4:** Expression of *asl*, *gogat*FD, *glnII*, *glnI* and *argJ* in *D*. *glomerata* nodules using relative quantitation of the Comparative CT Method (ABI Prism user bulletin #2).

	*asl*	*gogatFD*	*glnII*	*glnI*	*argJ*
**average**	1.26	4.41	2.4	1.33	0.5
**SD**	0.17	0.11	0.15	0.18	0.19

The expression level of *gogatFD* is significantly higher than those of *asl*, *glnI*, *glnII* and *argJ*, but all the genes encoding N-assimilatory enzymes are expressed. The reference gene used was 16S rRNA.

### Nodule occupancy

Since the microsymbionts of *D*. *glomerata* cannot be cultured, it cannot easily be ascertained how many strains occupy an individual nodule, whether nodules house *Frankia* exclusively or in combination with other bacteria. In addition, it was important to detect any major contribution to nodule occupancy by a non-*Frankia* taxon that could carry accessory nodulation genes (e.g. *nodABC*). To shed light on these questions, *D*. *glomerata* nodules induced by *Frankia* inoculant originating in Pakistan [21] and grown at Stockholm University (SU), and *D*. *glomerata* nodules induced by a California source of *Frankia*, and grown at the University of California in Davis (UCD), respectively, were used for DNA isolation and analysis of operational taxonomic units (OTUs) *via* PCR amplification with universal 16S rDNA primers 27F and 388R and 454 sequencing (SRA BioProject PRJNA258479).

**Fig 6:**
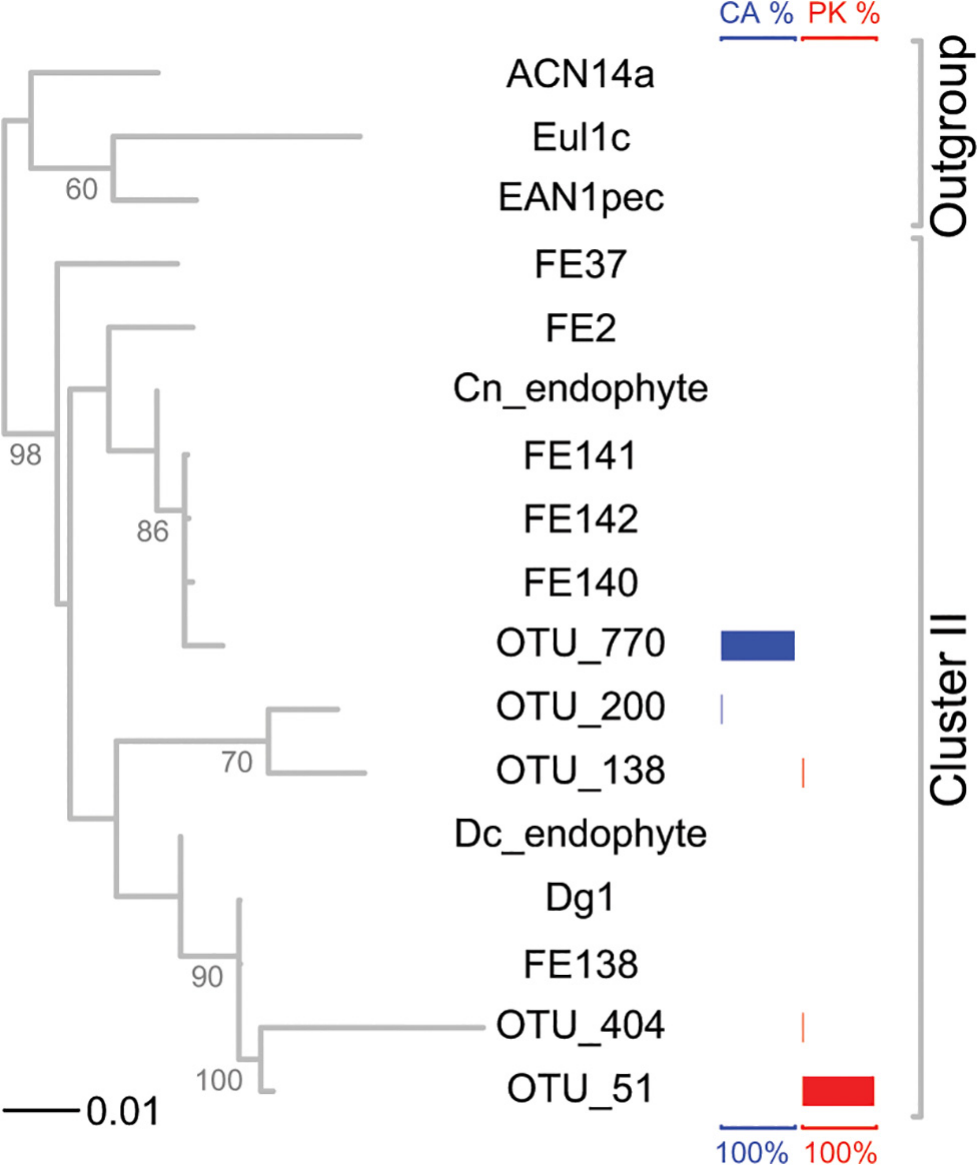
Neighbor-joining tree of *Frankia* OTUs observed in *Datisca glomerata* plants inoculated with material from either Pakistan or California combined with other Cluster II *Frankia* strains. Cluster I and III *Frankia* strains are used as outgroups. Branches with bootstrap values of 50 or higher are indicated in the figure. *: Cn endophyte sequence only contains v2 region. Relative abundances of each *Frankia* OTU are shown in colored bars to the right; blue indicates each OTU's percent of *Frankia* reads in nodules of plants inoculated with material from California (CA), red indicates percent in nodules of plants inoculated with material from Pakistan (PK).

Most reads were identified as one of five OTUs of *Frankia*, which represented the majority of bacterial reads (68% of the bacterial reads from SU nodules and 84% of the bacterial reads from UCD nodules; see Table 5; S7 Table). Two *Frankia* OTUs contributed most of the *Frankia* reads: OTU 51 from SU nodules (Pakistan inoculant) and OTU 770 from UCD nodules (California inoculant), representing 96–98% and 99–99.6% of *Frankia* reads within each sample, respectively. As shown in the neighbor-joining tree (Fig 6), all the *Frankia* OTUs formed a clade within Cluster II. OTU 51 and OTU 770 belonged to two subclades that were weakly-supported statistically; however these sequences differed by 12 base-pairs (>3.7%; Fig 6). The other *Frankia* OTUs belonged to a clade either with OTU 51 or with OTU 770.

**Table 5:** Processed bacterial sequences and taxonomy assignments by sample source.

Taxonomy					Dg_UCD^b^		Dg_SU^c^		#OTUs^e^
Phylum	Class	Order	Family	Genus	Mean	SD^d^	Mean	SD^d^	
Actinobacteria	Actinobacteria	Frankiales	Frankiaceae	*Frankia*	85.96%	3.99%	68.29%	1.27%	9
		Mycobacteriales	Mycobacteriaceae	*Mycobacterium*	0.01%	0.01%	1.34%	0.39%	2
		Streptomycetales	Streptomycetaceae	*Streptomyces*	0.26%	0.19%	0.03%	0.01%	1
Bacteroidetes	Sphingobacteria	Sphingobacteriales	Flexibacteraceae	*Cytophaga*	0.06%	0.06%	1.81%	0.18%	2
				*Dyadobacter*	0.32%	0.33%	0.15%	0.06%	1
Proteobacteria	Alpha-proteobacteria	Caulobacterales	Caulobacteraceae	*Caulobacter*	0.11%	0.06%	0.46%	0.05%	1
		Rhizobiales	Bradyrhizobiaceae	*Afipia*	0.32%	0.11%	0.05%	0.03%	1
		Sphingomonadales	Sphingomonadaceae	*Novosphingobium*	0.28%	0.31%	0.28%	0.14%	1
				*Sphingobium*	0.23%	0.17%	0.04%	0.01%	1
	Beta- proteobacteria	Burkholderiales			0.21%	0.20%	0.48%	0.01%	1
		Methylophilales	Methylophilaceae	*Methylophilus*	0.25%	0.21%	0.23%	0.18%	1
	Delta-proteobacteria	Myxococcales			0.04%	0.02%	0.65%	0.18%	1
TM7	TM7-3	EW055			0.0%	0.0%	0.39%	0.15%	1
**Unassigned** ^**a**^					5.69%	0.15%	6.87%	1.37%	5
**Low abundance** ^**a**^					6.27%	2.49%	18.94%	0.05%	789
**Total bacteria reads and OTUs**					3424	2006	3535	974	817

^a^Low abundance OTUs (less than 0.1% of bacterial sequences), and OTUs not assignable to any taxonomy are summed across OTUs.

^b^Dg_UCD are *D*. *glomerata* nodule samples from University of California Davis, inoculated with a Californian source.

^c^Dg_SU are *D*. *glomerata* nodule samples from Stockholm University, inoculated with a Pakistani source.

^d^Standard deviations are based on n = 2.

^e^# OTUs refers to the number of OTUs at 97% identity in each taxonomic category.

While *Frankia* dominated the nodule bacterial component, other taxa were minor components (less than 2% of bacterial reads from any individual sample; Table 5). Of the taxa besides *Frankia* that were detected in nodules, only *Mycobacterium*, *Cytophaga*, and an OTU in the Myxococcales exceeded 0.2% of the total reads in any sample (Table 5). *Mycobacterium* and *Cytophaga* are known plant endophytes [86,87]. Some low-abundance OTUs (<0.2% of total reads) were also assigned to genera that include known plant endophytes or rhizosphere bacteria, e.g. *Streptomyces*, *Dyadobacter*, *Caulobacter*, *Novosphingobium*, *Sphingobium*, and *Methylophilus* [88,89,90,91,92]. Identification to the order level only for the OTU in the Myxococcales is too broad to infer any ecological characters, however this clade does include species commonly found in soil. None of the OTUs in the nodule samples were assigned to genera reported to possess *nodABC* genes.

## Discussion

### Is *Candidatus* Frankia datiscae Dg1 an obligate symbiont?

The first genome sequence of a representative of the non-cultured Cluster II *Frankia* strains was expected to answer the question of whether these strains were obligate symbionts or just had (an) auxotrophy/auxotrophies that made attempts at isolation very difficult. The data obtained in this study do not point to any obvious auxotrophy, however the comparatively low capacity for the synthesis of secondary metabolites (S4 Table) and the loss of the gas vesicle protein (*gvp*) genes indicate a reduced ability to thrive in the highly competitive soil biotope.

The genome sizes of *Frankia* strains have been suggested to indicate the strains’ saprotrophic capacities [11]. The genome size of Dg1, which is smaller than, but close to that of the *Casuarina*-infective strain CcI3, suggests a low saprotrophic potential but does not make as definitive a case for genome reduction as might be expected in comparison with animal symbionts [71,93]. However, when comparing the reduction of the mitochondrial genomes (14–42 kB vs. 184–2400 kB; [94]), animal symbionts seem to be more impacted with regard to genome reduction than plant symbionts. Similarly, genome reduction in evolutionary younger plant symbionts seems to be much less dramatic than in animal symbionts; e.g., the obligate endophyte *Rhizophagus irregularis*, the symbiosis of which goes back at least 460 My [95] still has a genome size of more than 140 Mb [96]. The fact that Glomalean fungi, while obligate endophytes, are not transmitted vertically, but have a pre-symbiotic growth phase in the soil, might explain the comparable lack of genome reduction. In this context, it should be pointed out that Cluster II *Frankia* strains are not transmitted vertically either. The fact that soil from underneath plants containing nodules induced by Cluster II *Frankia* strains can be used to infect new plants indicates that Cluster II *Frankia* strains, like Glomalean fungi, have a pre-symbiotic growth phase in soil. Furthermore, the identification of a Cluster II *Frankia* strain in soil devoid of any compatible host plant species [97] points at some saprotrophic capability. Also the fact that the Dg1 genome contains several operons for the biosynthesis of siderophores and other secondary metabolites that are not expressed in nodules (S4 Table), one of them containing a gene encoding protein of 6077 amino acids (WP_013873316.1) indicates some selection pressure to maintain these operons, and thus, (a) non-symbiotic growth phase(s).

Host plants of Cluster II strains are distributed in the Americas, the Mediterranean, Asia, Oceania and New Zealand. The low genetic diversity of Cluster II strains, indicating the likelihood of an evolutionary bottleneck, was first suggested by Vanden Heuvel et al. [16]. Our findings add further support to this interpretation. The transcriptome of the Cluster II strain from California presented here (represented by OTU 770) mapped with relatively few polymorphisms to the genome of Dg1, which originated in Pakistan (represented by OTU 51), despite the disjunct geographic distribution of the host plant genera of Cluster II strains, most strikingly of *Coriaria*.

Yokoyama et al. [20] came to the conclusion that the disjunct distribution pattern of *Coriaria* spp. was the result of several geographical migrations and separations in the Cenozoic. Since *Frankia* propagules can be distributed by birds [98], by waterways [99], and potentially by other vectors, a compatible microsymbiont might have been distributed along with the host plant seeds. Thus, Cluster II strains might have been spread together with their host plants (Datiscaceae and Coriariaceae), while the stability of their ecological niche prevented further genome diversification.

### Why is the basal clade of symbiotic *Frankia* strains saprotrophically challenged?

The result that Cluster II is the basal clade of symbiotic *Frankia* strains is consistent with plant phylogenetic results implying that the oldest actinorhizal symbioses are those of Cucurbitales [4].

Non-symbiotic, non-nitrogen fixing so-called *Frankia*-like strains or atypical *Frankia* strains (Cluster IV) were considered to be basal to symbiotic *Frankia* strains [7,56,100]; however, this is contradicted by more recent analysis by Sen et al. [10] who find that cluster II, as represented by Dg1, is basal to all other *Frankia* strains including the atypical ones. So far, most atypical strains were isolated from nodules induced by Cluster II strains. Since these strains are unable to induce nodules on their own, they may have represented contaminations from the nodule periderm. The fact that like Cluster I and Cluster III *Frankia* strains, these Cluster IV *Frankia* strains could be cultured suggests that their saprotrophic capabilities are broader than those of Cluster II strains. The genome sequence of one of these strains, CN3 [101] comprises 10 MB and represents the largest *Frankia* genome known to date, while being still in the range of the genome sizes of cluster III strains.

Small genomes (5.27–5.6 MB) also are found among the *Casuarina-*infective subgroup of Cluster I [11,102] which have a narrow host range of plants with a narrow native distribution, and the small genome size of the *Casuarina*-infective strains are assumed to be due to genome shrinkage [11]. This phytogeographic context does not represent the situation of Cluster II strains which have a broad host range, and a broader geographic distribution. Dg1 has more than 800 genes not present in any other *Frankia* genome available in the JGI database. Most strikingly, only Dg1 among sequenced *Frankia* genomes contains a copy (AEH09479-AEH09484) of the mammalian cell entry (*mce*) gene cluster from *Mycobacterium tuberculosis*, assumed to be involved in the uptake of sterols, which in *Streptomyces coelicolor* is required for plant root colonization [103]. So we speculate that the ancestors of all *Frankia* strain were rhizosphere bacteria with large genomes and broad saprotrophic potential when one subgroup—the progenitors of Cluster II—acquired the gene set for nitrogen fixation and evolved symbiotic capabilities. Symbiosis led to genome reduction in this subgroup. In due time, two other subgroups (the progenitors of Clusters I and III, respectively) developed symbiotic capabilities; later, a subgroup of Cluster I—the *Casuarina*-infective strains—underwent genome shrinkage.

Interestingly, several of the Dg1 operons involved in the synthesis of secondary metabolites contain genes that are expressed in nodules of *D*. *glomerata* (S4 Table), so even though secondary metabolism loci are reduced overall, the corresponding functions do seem to play a role in symbiosis and are not only relevant during saprotrophic growth.

### Nodule occupancy

Cluster II *Frankia* were the predominant bacteria detected in root nodules of *D*. *glomerata* inoculated with sources from two geographically distinct locations, Pakistan and California, in separate experiments. Other bacterial taxa occurred at extremely low levels, indicating that there are no major co-symbionts in the root nodules.

The topology of the neighbor-joining tree for the nodule *Frankia* OTUs is broadly consistent with the findings of Vanden Heuvel et al. [16]. Two distinct strains of Cluster II *Frankia* (represented by OTU 51 (Dg1) and OTU 770) were detected in nodules induced by the Pakistan and the California source, respectively. Interestingly, OTU 51 (Pakistan) clusters strongly with FE138, which was detected in field nodules in California [16]; and OTU 770 groups phylogenetically with Cn endophyte, a strain detected in soil beneath *Coriaria nepalensis* from Pakistan [104], and with strains detected in western North America [16] (Fig 6). These findings indicate a phylogenetic overlap in the composition of the respective communities of Cluster II *Frankia* strains.

### Transcriptome analysis

Taken together, the analysis of the most abundantly-expressed *Frankia* biosynthetic pathways in young root nodules indicate that nitrogen fixation and associated processes comprise the predominant nodule activity of *Frankia*. At the same time, the data suggest that Nod factor biosynthesis occurs in these young nodules. It has been shown that rhizobia express their *nod* genes inside the nodule until bacteroid differentiation [105], and components of the common symbiotic signal transduction pathway are expressed in the apices of nitrogen-fixing nodules [106].

Genes for enzymes involved in nitrogen assimilation and amino acid biosynthesis, including GS-GOGAT, branched-chain amino acid biosynthesis, and the complete arginine biosynthesis pathway, were expressed in Dg1 in symbiosis. These findings provide further evidence for a model of novel partitioning of nitrogen assimilation in *D*. *glomerata* nodules in which the expression of plant glutamine synthetase in the uninfected cells surrounding the infected cells, not in the infected cells themselves, indicated that the microsymbiont likely exports amino acids, not ammonium [107,108]. Physiological studies had suggested that the nitrogen export form was arginine [107]. The high expression of the arginine biosynthetic pathway confirms an important role for arginine biosynthesis as a major intermediate nitrogen storage pathway in cucurbitoid nodule N assimilation [107]. The abundance of transcripts encoding enzymes from the branched chain amino acid pathway was surprising since it has been shown that at least in pea and bean nodules, synthesis of branched chain amino acids is downregulated in symbiosis and they are provided by the host plant [109], although this is not the case for all rhizobial symbioses [110]. The expression of these complex amino acid biosynthetic pathways is a likely further example of a degree of metabolic independence of *Frankia* Dg1 in this symbiosis, compared with legume-rhizobia symbioses or other *Frankia* symbioses [85].

Active expression of the isoprene biosynthetic pathway, with mRNAs in the 90% quantile, was expected as it is responsible for the synthesis of hopanoids, bacterial steroid lipids that are part of the envelope of nitrogen-fixing *Frankia* vesicles [111], and menaquinone, required for electron transport.

### Which bacterial group “invented” lipochitooligosaccharide (LCO) Nod factors?

The host receptors for microsymbiont signaling molecules that use the common symbiotic signaling pathway [112] evidently evolved from chitin receptors [113]. While arbuscular mycorrhizal fungi use LCOs and short-chain chitin oligomer (COs) for signaling to their plant hosts [23,31], rhizobia, with a few exceptions [33], seem to use only LCOs. It is unknown thus far which signal factors are used by *Frankia* strains in Clusters I and III.

Rhizobia, the microsymbionts of legumes and *Parasponia*, are polyphyletic. Most rhizobia belong to the α-proteobacteria, but several legumes are nodulated primarily by β-proteobacteria (*Burkholderia* spp., *Cupriavidus* spp.; [114]). It has been assumed that the nodulation genes originated in α-rhizobia and that β-rhizobia gained them through multiple lateral *nod* gene transfers [115], but our phylogenetic analyses now indicate that *nodA* and *nodB* are likely to have been transferred originally from actinobacteria to rhizobia; the relationships among the *nodC* genes are less certain.

The Dg1 protein sequences of NodA and NodC are basal to the rhizobial orthologs, and the two Dg1 NodB proteins also have basal positions in the NodB phylogeny (Fig 3). The functional role of the NodA protein in symbiosis is to transfer an acyl chain to the backbone of Nod factor precursor chito-oligosaccharides, assembled by NodC and deacetylated at the non-reducing end by NodB. This NodA-mediated acyl group transfer is key to Nod factor signaling function, rendering the LCO amphiphilic, and presumably permitting it to reach the host membrane-localized LysM receptor kinase [116]. The *nodB* and *nodC* genes are members of multigene families, coding for polysaccharide deacetylases and glycosyl transferases, respectively, with homologs occurring throughout the eubacteria. In contrast, *nodA* genes are not part of a ubiquitous gene family with duplications, a difference reflected in the phylogenetic trees (Figs 2, 3 and 4). Thus, the existence of a series of NodA-like acyl transferases across the phylum Actinobacteria but otherwise only in the nodulating members of the Proteobacteria supports an actinobacterial origin of *nodA* genes, and thus of LCO Nod factors. The detailed phylogeny suggests that the common ancestor of Dg1 and *S*. *bottropensis* specifically among the actinobacteria was the source of the rhizobial nodulation protein. The fact that α- and β-Proteobacteria lack NodA paralogs further supports the integrity of the overall topology of the tree.

Thus, the phylogenetic evidence suggests that NodA-type acyl-transferases first evolved in Actinobacteria and then laterally transferred to rhizobia. The positioning of *nodA* genes in the non-*Frankia* actinobacterial genomes tentatively suggests a link with cell wall biosynthesis, and it is plausible that an acyltransferase is involved in the biosynthesis of actinobacterial cell walls as many of them (though not those of *Frankia* sp.) contain mycolic acids which are linked to the peptidoglycan [117]. An origin of *nodA* (or *nodABC*) outside the Proteobacteria would be analogous to the situation of *nodIJ*, which evolved in β-Proteobacteria and were subsequently acquired by α-Proteobacteria *via* lateral gene transfer [80]. Interestingly, also in Dg1 the *nodB2C* operon was found to be linked to a *nodIJ*-like operon. However, phylogenetic analysis of NodI and NodJ homologs (S6 Fig) showed that the linkage between *nodBC* and *nodIJ* type genes is unlikely to be based on lateral gene transfer from Actinobacteria, but seems to have evolved independently in Actinobacteria and β-Proteobacteria (and later in α-Proteobacteria [80]).

The relatively low GC content of the *nod* genes in Dg1 relative to the whole genome, and similar patterns in the corresponding rhizobial genes, suggest a comparatively rapid evolution of these genes, related to the functional specialization of symbiosis. It is conceivable that multiple lateral gene transfer between the two phyla could explain the relatively low GC content of the *nod* genes relative to genomic GC content. However, this is not a scenario that is plausible based on any of the strongly-supported lines of phylogenetic evidence.

No close homologs of the canonical *nod* genes could be identified in the sequenced genomes of any *Frankia* strain of Cluster I or Cluster III, as can be seen in the phylogenetic trees. Thus in *Frankia*, the *nod* gene orthologs seem to be exclusive to Cluster II. *Nod* gene expression in nodules was observed not only in Dg1 in symbiosis with *D*. *glomerata* as described here; expression has also been detected in *Ceanothus velutinus* nodules collected in California (T. Persson, A.M. Berry and K. Pawlowski, unpublished observations). It is all the more striking that this strain—or group of strains—that has one of the smallest genomes and the largest number of pseudogenes of *Frankia* strains analyzed so far, and the most reduced number of gene clusters for the synthesis of secondary metabolites (S4 Table), nevertheless retains canonical *nod* genes. Assuming that the Dg1 *nod* genes expressed in symbiosis catalyze the production of LCOs for signaling to host plants, and given the well-demonstrated basal position of Cluster II genes relative to other *Frankia*, shown both here and in studies of a wide range of genes (e.g., [10]), it seems most likely that the common ancestor of all the symbiotic *Frankia* strains contained the canonical *nod* genes which were transmitted to the rhizobia, but subsequently lost in the progenitor of *Frankia* Clusters I and III. At any rate, the fact that Dg1 cannot be cultured so far has prevented any isolation of LCO Nod factors formed by this strain, as nodules proved to be too complex a source to isolate LCOs present at very low concentrations.

## Conclusions

In summary, the results of the analysis of the first genome of a Cluster II *Frankia* strain, Dg1, supports the hypothesis that symbioses between Cluster II *Frankia* strains and actinorhizal Cucurbitales are likely to represent the oldest actinorhizal symbioses. Comparative analysis of *Frankia* genomes revealed more than 800 unique genes in Dg1. Among those are rhizobial-type canonical *nod* genes, which are expressed in symbiosis and, based on detailed phylogenetic analysis, are likely to have originated in Actinobacteria. Transcriptome analysis supported the hypothesis that Cluster II strains in nodules export an assimilated form of nitrogen, rather than ammonium, most likely arginine. Analyses of *D*. *glomerata* nodule occupancy via 454 OTU sequencing showed that (a) more than one strain was found in a nodule lobe, and (b) seemingly the same, or at least very similar strains were present in a Californian inoculant and in an inoculant originating in Pakistan, though two different strains were dominating in both inoculants. Transcriptome analysis underlined the low genetic diversity between the genomes of these different strains.

## Supporting Information

S1 Fig Comparison of theoretical and empirical CDF function for rpkb normalized transcriptome fit to a negative binomial distribution. The cumulative distribution function (CDF) for the rpkb data is illustrated as black circular data points. The CDF generated from the fit of a negative binomial function to the empirical data is illustrated as a solid red line.
(DOCX)

S2 Fig Venn diagram showing the core genome of *Frankia* strains ACN14a, CcI3, EAN1pec and Dg1 as well as genes specific to individual strains or groups of strains. The core genome between the four sequenced *Frankia* strains (ACN14a, CcI3, EAN1pec, CcI3, and Dg1) was calculated using EDGAR (http://edgar.cebitec.uni-bielefeld.de; [59]).
(DOCX)

S3 Fig IS elements in the genomes of different *Frankia* strains. (A) Fraction of the genome consisting of IS elements in different *Frankia* strains. Detailed analysis of IS elements in two representatives of the basal non-symbiotic strains (CN3, EuI1c), in four strains of cluster I (two *Alnus*-infective strains, ACN14a and QA3, and two *Casuarina*-infective strains, CcI3 and BMG5.12), one representative of cluster II (Dg1) and four representatives of cluster III (two *Elaeagnus*-infective strains, EUN1f and EAN1pec, and one *Discaria*-infective strain, BCU110501) showed that three genomes among those analysed—those of CcI3, Dg1 and EAN1pec—show an increase in relative amounts of IS elements, and that among these three, the Dg1 genome contains the highest relative amount of IS elements. (B) Distribution of size of IS elements in *Frankia* strains.
(DOCX)

S4 Fig *Frankia* phylogeny using the four published genomes of strains ACN14a (Fa) and CcI3 (Fc, Cluster I), Dg1 (Fd, Cluster II) and EAN1pec (Fe, Cluster III). *Acidothermus cellulolyticus* 11B (Acido), *Stackebrandtia nassauensis* DSM 44728 (Stack), *Geodermatophilus obscurus* DSM 43160 (Go), *Nakamurella multipartita* DSM 44233 (Naka), and *Thermobifida fusca* YX (Thermo) were used as outgroups. Forty housekeeping genes were analyzed. Each gene sequence was identified in *Candidatus* Frankia datiscae Dg1. After identification, the gene was used in a Blast search as the query. The corresponding Blast was restricted to *Frankia alni* ACN14a, *Frankia* sp. Ccl3, *Frankia* sp. EaN1pec, *A*. *cellulolyticus* 11B, *G*. *obscurus* DSM 43160, *S*. *nassauensis* DSM 44728, *N*. *multipartita* DSM 44233 and *T*. *fusca* YX. All alignments were created using MUSCLE (multiple sequence comparison by log- expectation; Edgar 2004) at the EMBL-EBI website. Maximum parsimony analyses were performed using the software package PAUP* version 4.0b10 (Swofford 1999). All characters were weighted equally and gaps in the alignment were treated as missing. A heuristic search strategy with 10 random replicates, TBR branch-swapping and the MULTREES optimization was used. MAXTREES parameter was set to 10,000. Support for branches was evaluated using bootstrap analysis (Felsenstein 1985) and random sequence addition for 100 replicates, using the same parameters.
(DOCX)

S5 Fig Alignment of amino acid sequences used for phylogenies. Identical amino acids in highly conserved positions are highlighted in blue, identical amino acids in less conserved positions are highlighted in grey. Results are depicted in the order NodA, NodB, NodC, NodI, NodJ.
(DOCX)

S6 Fig Maximum Likelihood trees of (A) NodI and (B) NodJ proteins. All sequences from Dg1 are given in red. Sequences from β-proteobacteria where the rhizobial *nodIJ* genes evolved are given in green, sequences from α-proteobacteria are given in turquoise. Names of actinobacterial NltI/NltJ sequences the genes of which are part of a *nodBCnltIJ* operon are indicated in blue. The sequences from *Streptomyces bottropensis* are given in purple. All sequences used for the phylogenetic analysis are given in S6 Table.
(PDF)

S1 Table Primers used in quantitative real time-PCR. (XLSX)

S2 Table List of all IS elements found in different *Frankia* genomes. (ZIP)

S3 Table Nine Frankia OTUs identifed in *D*. *glomerata* nodules in this study are listed along with the number of reads that belong to each OTU in each sample. One inoculant goes back to a *D*. *glomerata* plant from California (UCD), the other one to a *Coriaria nepalensis* plant from Pakistan (SU).
(XLSX)

S4 Table Secondary metabolites pathways present in Frankia strains from Cluster I (ACN14a, CcI3), Cluster III (EAN1pec) and Cluster II (Dg1). The analysis of the genome sequences with regard to biochemical pathways in Dg1 was performed using Pathway tools [47], MAGE, IMG/ER and based on Udwary et al. [67].
(XLSX)

S5 Table Analysis of various genome characteristics in *Frankia* strains ACN14a CcI3, EaN1pec and Dg1. Palindromic Repeats were analyzed using the palindrome tool from EMBOSS (http://bips.u-strasbg.fr/EMBOSS/) with no mismatches and the following parameters: 1. Repeat units between 8 and 11 bases with up to a 3 base gap. 2. Repeat units between 12 and 19 bases with up to a 7 base gap. 3. Repeat units between 20 and 90 bases with up to a 20 base gap. 4. Repeat units less than 12 bases must occur at least 10 times in the genome. 5. Repeat units less than 20 bases must occur twice in the genome. Tandem repeats were analyzed with the MUMmer 3.13 package (http://www.tigr.org/software/mummer/) with the following parameters: Minimum match length = 20 bases. 2. It is assumed that one copy of a tandem repeat in a genome is not very significant unless it is long. Therefore, a genome-wide screen for the repeat used was added. The total number of bases incorporated into repeats for a particular repeat unit must total 50 or more bases.
(DOCX)

S6 Table Sequences used for phylogenies. (XLSX)

S7 Table List of *Frankia* strains used in the phylogenetic analysis and references. (DOCX)
