# Supplementary material for: Candidatus Frankia Datiscae Dg1, the Actinobacterial Microsymbiont of Datisca glomerata, Expresses the Canonical nod Genes nodABC in Symbiosis with Its Host Plant
Source: PLoS One. 2015 May 28;10(5):e0127630. doi: 10.1371/journal.pone.0127630 (PMC4447401; doi:10.1371/journal.pone.0127630)

**S1 Fig. Comparison of theoretical and empirical CDF function for rpkb normalized transcriptome fit to a negative binomial distribution.** The cumulative distribution function (CDF) for the rpkb data is illustrated as black circular data points. The CDF generated from the fit of a negative binomial function to the empirical data is illustrated as a solid red line.


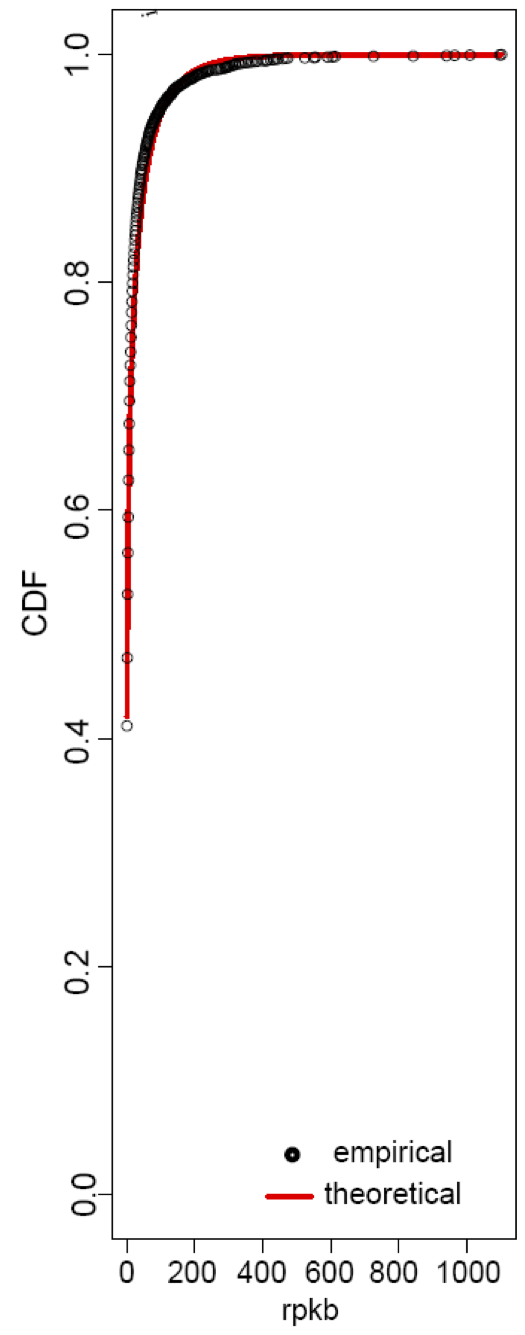

Supplement: S1 Fig — The cumulative distribution function (CDF) for the rpkb data is illustrated as black circular data points. The CDF generated from the fit of a negative binomial function to the empirical data is illustrated as a solid red line. (DOCX) [file pone.0127630.s001.docx]
