# Supplementary material for: Candidatus Frankia Datiscae Dg1, the Actinobacterial Microsymbiont of Datisca glomerata, Expresses the Canonical nod Genes nodABC in Symbiosis with Its Host Plant
Source: PLoS One. 2015 May 28;10(5):e0127630. doi: 10.1371/journal.pone.0127630 (PMC4447401; doi:10.1371/journal.pone.0127630)

**S2 Fig. Venn diagram showing the core genome of *Frankia* strains ACN14a, CcI3, EAN1pec and Dg1 as well as genes specific to individual strains or groups of strains.** The core genome between the four sequenced *Frankia* strains (ACN14a, CcI3, EAN1pec, CcI3, and Dg1) was calculated using EDGAR (<http://edgar.cebitec.uni-bielefeld.de>; [59]).


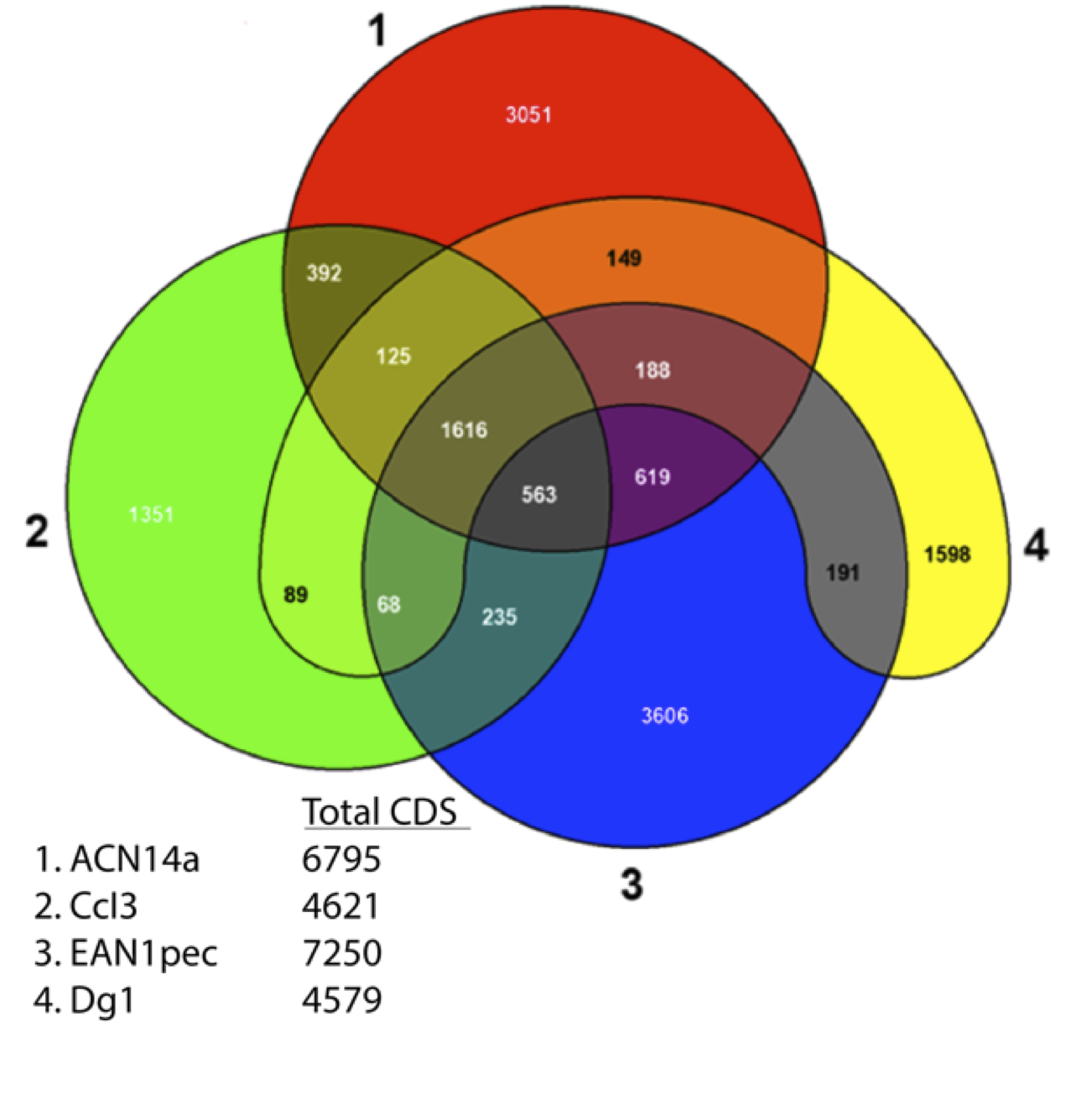

Supplement: S2 Fig — The core genome between the four sequenced Frankia strains (ACN14a, CcI3, EAN1pec, CcI3, and Dg1) was calculated using EDGAR (http://edgar.cebitec.uni-bielefeld.de; [59]). (DOCX) [file pone.0127630.s002.docx]
