# Supplementary material for: Candidatus Frankia Datiscae Dg1, the Actinobacterial Microsymbiont of Datisca glomerata, Expresses the Canonical nod Genes nodABC in Symbiosis with Its Host Plant
Source: PLoS One. 2015 May 28;10(5):e0127630. doi: 10.1371/journal.pone.0127630 (PMC4447401; doi:10.1371/journal.pone.0127630)
Supplement: S3 Fig — (A) Fraction of the genome consisting of IS elements in different Frankia strains. Detailed analysis of IS elements in two representatives of the basal non-symbiotic strains (CN3, EuI1c), in four strains of cluster I (two Alnus-infective strains, ACN14a and QA3, and two Casuarina-infective strains, CcI3 and BMG5.12), one representative of cluster II (Dg1) and four representatives of cluster III (two Elaeagnus-infective strains, EUN1f and EAN1pec, and one Discaria-infective strain, BCU110501) showed that three genomes among those analysed—those of CcI3, Dg1 and EAN1pec—show an increase in relative amounts of IS elements, and that among these three, the Dg1 genome contains the highest relative amount of IS elements. (B) Distribution of size of IS elements in Frankia strains. (DOCX) [file pone.0127630.s003.docx]

**S3 Fig. IS elements in the genomes of different *Frankia* strains.** (A) Fraction of the genome consisting of IS elements in different *Frankia* strains. Detailed analysis of IS elements in two representatives of the basal non-symbiotic strains (CN3, EuI1c), in four strains of cluster I (two *Alnus*-infective strains, ACN14a and QA3, and two *Casuarina*-infective strains, CcI3 and BMG5.12), one representative of cluster II (Dg1) and four representatives of cluster III (two *Elaeagnus*-infective strains, EuN1f and EAN1pec, and one *Discaria*-infective strain, BCU110501) showed that three genomes among those analysed – those of CcI3, Dg1 and EAN1pec - show an increase in relative amounts of IS elements, and that among these three, the Dg1 genome contains the highest relative amount of IS elements. (B) Distribution of size of IS elements in *Frankia* strains.

**
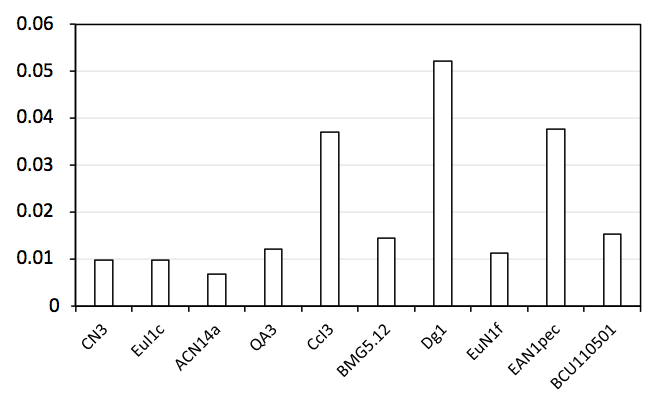
A**

**
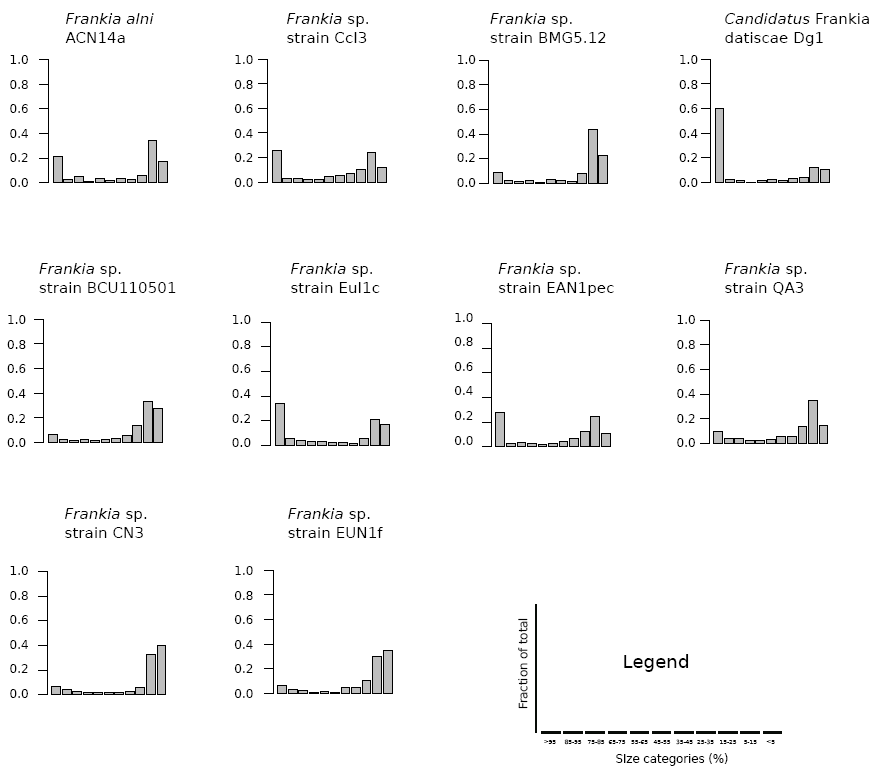
B**
