# Supplementary material for: Candidatus Frankia Datiscae Dg1, the Actinobacterial Microsymbiont of Datisca glomerata, Expresses the Canonical nod Genes nodABC in Symbiosis with Its Host Plant
Source: PLoS One. 2015 May 28;10(5):e0127630. doi: 10.1371/journal.pone.0127630 (PMC4447401; doi:10.1371/journal.pone.0127630)
Supplement: S4 Fig — Acidothermus cellulolyticus 11B (Acido), Stackebrandtia nassauensis DSM 44728 (Stack), Geodermatophilus obscurus DSM 43160 (Go), Nakamurella multipartita DSM 44233 (Naka), and Thermobifida fusca YX (Thermo) were used as outgroups. Forty housekeeping genes were analyzed. Each gene sequence was identified in Candidatus Frankia datiscae Dg1. After identification, the gene was used in a Blast search as the query. The corresponding Blast was restricted to Frankia alni ACN14a, Frankia sp. Ccl3, Frankia sp. EaN1pec, A. cellulolyticus 11B, G. obscurus DSM 43160, S. nassauensis DSM 44728, N. multipartita DSM 44233 and T. fusca YX. All alignments were created using MUSCLE (multiple sequence comparison by log- expectation; Edgar 2004) at the EMBL-EBI website. Maximum parsimony analyses were performed using the software package PAUP* version 4.0b10 (Swofford 1999). All characters were weighted equally and gaps in the alignment were treated as missing. A heuristic search strategy with 10 random replicates, TBR branch-swapping and the MULTREES optimization was used. MAXTREES parameter was set to 10,000. Support for branches was evaluated using bootstrap analysis (Felsenstein 1985) and random sequence addition for 100 replicates, using the same parameters. (DOCX) [file pone.0127630.s004.docx]

**S4 Fig. *Frankia* phylogeny using the four published genomes of strains ACN14a (Fa) and CcI3 (Fc, Cluster I), Dg1 (Fd, Cluster II) and EAN1pec (Fe, Cluster III).** *Acidothermus* *cellulolyticus* 11B (Acido), *Stackebrandtia nassauensis* DSM 44728 (Stack), *Geodermatophilus* *obscurus* DSM 43160 (Go), *Nakamurella* *multipartita* DSM 44233 (Naka), and *Thermobifida* *fusca* YX (Thermo) were used as outgroups. Forty housekeeping genes were analyzed.

 Each gene sequence was identified in *Candidatus* Frankia datiscae Dg1. After identification, the gene was used in a Blast search as the query. The corresponding Blast was restricted to *Frankia alni* ACN14a, *Frankia* sp. Ccl3, *Frankia* sp. EaN1pec, *A.* *cellulolyticus* 11B, *G. obscurus* DSM 43160, *S. nassauensis* DSM 44728, *N.* *multipartita* DSM 44233 and *T.* *fusca* YX. All alignments were created using MUSCLE (multiple sequence comparison by log- expectation; Edgar 2004) at the EMBL-EBI website. Maximum parsimony analyses were performed using the software package PAUP* version 4.0b10 (Swofford 1999). All characters were weighted equally and gaps in the alignment were treated as missing. A heuristic search strategy with 10 random replicates, TBR branch-swapping and the MULTREES optimization was used. MAXTREES parameter was set to 10,000. Support for branches was evaluated using bootstrap analysis (Felsenstein 1985) and random sequence addition for 100 replicates, using the same parameters.

Fc

Fa

Fe

Fd

Acido

Thermo

Stack

Geo

Naka

**All 50 gene**

**Analysis**

**NJ**

100

100

99

100

100

100

Fc

Fa

Fe

Fd

Acido

Thermo

Stack

Geo

Naka

**All 50 gene**

**Analysis**

**MP**

100

100

100

100

100

100

Fc

Fa

Fe

Fd

Go

Thermo

Stack

Naka

Acido

**81**

**77**

**aroK**

Fc

Fa

Fe

Fd

Go

Thermo

Stack

Naka

Acido

**16S 23S**

**100**

**100**

**100**

**63**

**88**

**97**

Fc

Fa

Fe

Fd

Go

Thermo

Stack

Naka

Acido

**atpA**

**100**

**97**

**97**

**76**

**79**

**55**

Fc

Fa

Fe

Fd

Go

Thermo

Stack

Naka

Acido

**bioA**

**100**

**80**

**62**

**100**

**73**

**100**

Fc

Fa

Fe

Fd

Thermo

Stack

Naka

Acido

**bioB**

**75**

**83**

**62**

**100**

**100**

Fc

Fa

Fe

Fd

Thermo

Stack

Naka

Acido

Go

**dapA**

**99**

**97**

**99**

**70**

**64**

Fc

Fa

Fe

Fd

Thermo

Stack

Naka

Acido

Go

**dnaA**

**100**

**99**

**81**

**57**

**53**

**97**

Fc

Fa

Fe

Fd

Thermo

Stack

Naka

Acido

Go

**folC**

**100**

**100**

Fc

Fa

Fe

Fd

Go

Thermo

Stack

Naka

Acido

**ftsZ**

**100**

**79**

**97**

**74**

**62**

**99**

Fc

Fa

Fe

Fd

Thermo

Stack

Naka

Acido

Go

**fusA**

**100**

**89**

**75**

**67**

**100**

Fc

Fa

Fe

Fd

Thermo

Stack

Naka

Acido

Go

**100**

**66**

**55**

**glpX**

Fc

Fa

Fe

Fd

Thermo

Stack

Naka

Acido

Go

**glmU**

**93**

**94**

**59**

**54**

**70**

Fc

Fa

Fe

Fd

Thermo

Stack

Naka

Acido

Go

**gltA**

**100**

**72**

**92**

**100**

**100**

**100**

Fc

Fa

Fe

Fd

Thermo

Stack

Naka

Acido

Go

**gyrA**

**100**

**96**

**95**

**56**

**82**

**97**

Fc

Fa

Fe

Fd

Thermo

Stack

Naka

Acido

Go

**ispA**

**100**

**95**

**72**

**69**

Fc

Fa

Fe

Fd

Stack

Naka

Acido

Go

**100**

**100**

**98**

**idi**

Fc

Fa

Fe

Fd

Go

Thermo

Stack

Naka

Acido

**murC**

**100**

**100**

**98**

**52**

**63**

Fc

Fa

Fe

Fd

Naka

Go

Stack

Acido

Thermo

**99**

**100**

**93**

**56**

**68**

**98**

**murA**

Fc

Fa

Fe

Fd

Naka

Go

Stack

Acido

Thermo

**92**

**100**

**80**

**mraY**

Fc

Fa

Fe

Fd

Stack

Naka

Acido

Go

Thermo

**69**

**78**

**100**

**93**

**lipA**

Fc

Fa

Fe

Fd

Stack

Naka

Acido

Go

**recA**

**61**

**99**

Fc

Fa

Fe

Fd

Go

Thermo

Stack

Naka

Acido

**murG**

**94**

**100**

**75**

**65**

Fc

Fa

Fe

Fd

Naka

Go

Stack

Acido

Thermo

**94**

**99**

**91**

**70**

**85**

**52**

**ribE**

Fc

Fa

Fe

Fd

Thermo

Stack

Naka

Acido

Go

**ribA**

**98**

**100**

**69**

**92**

**73**

Fc

Fa

Fe

Fd

Acido

Go

**100**

**61**

**shc**

Fc

Fa

Fe

Fd

Thermo

Stack

Naka

Acido

Go

**rpoB**

**100**

**93**

**72**

**62**

**76**

**96**

Fc

Fa

Fe

Fd

Go

Thermo

Stack

Naka

Acido

**trpE**

**76**

**99**

**100**

**74**

**68**

**92**

Fc

Fa

Fe

Fd

Thermo

Stack

Naka

Acido

Go

**ribF**

**100**

**61**

**87**

**76**

Fc

Fa

Fe

Fd

Go

Thermo

Stack

Naka

Acido

**ksgA**

**50**

**100**

**50**

Fc

Fa

Fe

Fd

Go

Stack

Acido

Thermo

**90**

**100**

**98**

**70**

**htpX**

Fc

Fa

Fe

Fd

Thermo

Stack

Naka

Acido

Go

**72**

**996**

**99**

**uppS**

Fc

Fa

Fe

Fd

Naka

Go

Stack

Acido

Thermo

**93**

**99**

**88**

**69**

**84**

**51**

**ribH**

Fc

Fa

Fe

Fd

Naka

Go

Stack

Acido

Thermo

**54**

**100**

**69**

**70**

**rplB**

Fc

Fa

Fe

Fd

Naka

Go

Stack

Acido

Thermo

**73**

**99**

**90**

**85**

**76**

**70**

**rplA**

Fc

Fa

Fe

Fd

Thermo

Stack

Naka

Acido

Go

**dnaB**

**100**

**99**

**99**

**53**

**83**

Fc

Fa

Fe

Fd

Go

Thermo

Stack

Naka

Acido

**lytB**

**99**

**62**

**76**

**54**

Fc

Fa

Fe

Fd

Thermo

Stack

Naka

Acido

Go

**rplC**

**100**

**77**

**74**

Fc

Fa

Fe

Fd

Go

Thermo

Stack

Naka

Acido

**rplD**

**94**

**95**

**85**

**56**

**60**

Thermo

Stack

Fc

Fa

Fe

Fd

Naka

Acido

Go

**rplF**

**100**

**100**

Fc

Fa

Fe

Fd

Thermo

Stack

Naka

Acido

Go

**rplE**

**89**

**53**

Fc

Fa

Fe

Fd

Go

Thermo

Stack

Naka

Acido

**dapB**

**82**

**80**

Fc

Fe

Fc

Fd

Acido

Thermo

Stack

Naka

Geo

**69**

**99**

**63**

**90**

**pgk**

**76**

Fa

Fc

Fe

Fd

Acido

Thermo

Stack

Naka

Geo

**97**

**100**

**79**

**tpiA**

Fc

Fe

Fa

Fd

Acido

Thermo

Geo

Naka

Stack

**eno**

**72**

**95**

**81**

**72**

**88**

Fa

Fc

Fe

Fd

Acido

Thermo

Naka

Stack

Geo

**94**

**97**

**82**

**metK**

**63**

**88**

Fc

Fe

Fa

Fd

Acido

Thermo

Naka

Stack

Geo

**87**

**83**

**100**

**clpP**

**88**

**58**

Fc

Fa

Fe

Fd

Thermo

Acid

Stack

Geo

Naka

**dxs**

**63**

**100**

**76**

**94**

**100**

Fc

Fe

Fa

Fd

Thermo

Geo

Acid

Stack

Naka

**98**

**100**

**92**

**dxr**

**59**

Fc

Fe

Fa

Fd

Thermo

Geo

Acid

Stack

Naka

**73**

**100**

**68**

**nth**

Fc

Fa

Fe

Fd

Acid

Thermo

Stack

Naka

Geo

**mfd**

**100**

**100**

**62**

**68**

**100**

Fa

Fc

Fe

Fd

Stack

Thermo

Geo

Acido

Naka

**94**

**100**

**100**

**aceE**

**59**
