# Supplementary material for: Candidatus Frankia Datiscae Dg1, the Actinobacterial Microsymbiont of Datisca glomerata, Expresses the Canonical nod Genes nodABC in Symbiosis with Its Host Plant
Source: PLoS One. 2015 May 28;10(5):e0127630. doi: 10.1371/journal.pone.0127630 (PMC4447401; doi:10.1371/journal.pone.0127630)
Supplement: S5 Fig — Identical amino acids in highly conserved positions are highlighted in blue, identical amino acids in less conserved positions are highlighted in grey. Results are depicted in the order NodA, NodB, NodC, NodI, NodJ. (DOCX) [file pone.0127630.s005.docx]

NodA alignment

WP_005476203.1 ------------------------------MkSQVqWktyWEhELKpSDHaELAEYFRtl

WP_012172341.1 mmvhnycapenvtapphvsrvtqynkrgirMiSKVtWrvaWEsdLtngDHaELsDFFKsv

CAC82897.1 ------------------------------MiSKVtWrvaWEsdLtngDHaELsDFFKsv

WP_013873450.1. ------------------------------MkTQIqWktyWENqLEpgDHaELAEFFRnt

AAK50870.1 ------------------------------MrSQlRWSvcWEsdLaVSDHlELsEFFqiA

WP_012555955.1 ------------------------------MsTgVRWkIsWENdLEpSDHaELsDFFRat

WP_028214790.1 ------------------------------MsSKVqWkLcWENELQLSDHlELAQFFRtt

ACC17805.1 ------------------------------MsSKVqWkLcWENELQLSDHlELAQFFRtt

CBM43078.1 ---------------------------------------------------ELAQFFRtt

CAC80544.1 ------------------------------MrSQVqWTLrWEsELQLdeHvELAsFFRnt

ACV52960.1 -----------------------------------RvkIsWENELQLgDHvELtEFFRkt

ZP_03519615.1 ------------------------------MrSEVRWkLcWEsELELADHvELAEFFRkt

ABO64758.1 ----------------------------------------WENELQLSDHvELcEFFRnt

CAC82838.1 ------------------------------MrcEVRWSLcWENELQLSDHiELtEFFRkA

ABO64759.1 ----------------------------------------WENELQLSDHiELtEFFRkA

CAC82889.1 -----------------MNIAVSrsAEepSarTQVqWSLrWENELQLADHaELADFFRns

CAJ41271.1 -----------------MNIAVSrsAEepyarTQVRWSLrWENELQLADHaELADFFRns

CAC82881.1 -----------------MNIvaSrtAEapSarTQVhWSvrWENELRLteHaELAEFFRks

ACA97556.1 -----------------MNIAVSptAEgaSarAQVqWSrrWEsELgLtDHaELsEFFRks

NP_768665.1 -----------------MNIAVSptAEgsSgrAQVqWSLrWEsELQLADHaELAEFFRks

WP_015930557.1 -------------mrfpsdLcpSsgtgelSarSQVRWrLcWENELELADHaELAEFlRkA

CAC42487.1 -----------------MNLAVStdrgplSvrSQVRWrLvWENELRLADHiELsEFFRkt

ABX80632.1 ----------------------------------------WENELRLvDHiELsEFFRkt

WP_013533527.1 ------------------------------MrSaVqWrLcWENnLQVADHvELsDFFRkt

CAC82870.1 ------------------------------MrpnVeWkLcWENELQLADHvELsDFFRkt

CAC82868.1 ------------------------------MrpnVdWkLcWENELQLADHvELsDFFRkt

ACT34095.1 ------------------------------MhSdVRWrLcWENELQLADHvELsDFFRkt

ACT34108.1 ------------------------------MrSdVRWrLcWENELQLADHvELsEFFRkt

YP_007685003.1 --------------------------------mgpRlSvvrEgaLdVADHeriAallalA

WP_012037425.1 ------------------------mtgryprpmgpRlTvvrEgaLdVADHegiAalltlA

JGI:640540037 ------------------------mtgryprpmgpRlTvvrEgaLdVADHegiAalltlA

YP_001710907.1 ------------------------mtgryprsmgpRlSvvrEgaLdVADHtsiAallalA

JGI:2553980777 ------------------------------vtddIRWevveEsaLtLADHeaitallaqA

JGI:2596277042 ------------------------------MtedIRWevveEstLsLpDHeaiAallgqA

JGI:2553420270 ----------------------------------------W-------------------

EYT63040.1 ------------------------------MtddIRWevveEstLsLpDHeaiAallgqA

WP_027465027.1 ------------------------------MtddIRWevveEstLsLpDHeaiAallgqA

WP_016698138.1 -----------------------------msveEpRWfhrWEgELtplDHlriAEllsaA

JGI:2585523411 -----------------------------msveEphWfhrWEgELaplDHlriAEllsaA

JGI:2599913978 -------------------------------mNElRWTLaWEsEtprerHegLAralals

YP_003135251.1 -------------------------------mNElRWTLaWEsEtprerHegLAralals

JGI:2574491345 ---------------------MpapAparpelqhIsWestpENdisplvHqQLntllRaA

WP_007498427.1 ----------------------------------------mENdvpadrHraLhllltes

JGI:2574392503 -------------------------------vdhltWAvsWENdLppegHdaLAgllarA

JGI:2515227178 --------------------mataartpaStdAglvWSrhWEtELgeteHdaiAlllaaA

WP_005476203.1 YGevnAw---aFEGnRSfAlgGARPEfRiIGYDdkGVA-AHtGvLRRFlrV--G------

WP_012172341.1 YGaTGAFNAlPFaGGRSWA--GARPELRgIaYDesGVA-AHMGvLRRFIKV--G------

CAC82897.1 YGaTGAFNAlPFaGGRSWA--GARPELRgIaYDesGVA-AHMGvLRRFIKV--G------

WP_013873450.1. YGavGAwNvKhFKGsRSWA--GARPELRiIGHDerGVA-AHfGvLRRFIrl--G------

AAK50870.1 YGlTGSsNAlPFmsnRSWA--GARPELRvIGYDkrGVA-AHLavLRRFIKV--e------

WP_012555955.1 YGPTGAFNAKPFEtGRSWA--GARPErRAIaYDSkGVA-sHMGLLRRFIKV--G------

WP_028214790.1 YGPTGSFNAKPFDGGRSWA--GARPELRAIaYDSqGVA-AHMGsLRRFIKV--G------

ACC17805.1 YGPTGSFNAKPFDGGRSWA--GARPELRAIaYDSqGVA-AHMGsLRRFIKV--G------

CBM43078.1 YGPTGSFNAKPFDGGRSWA--GARPELRAIaYDSqGVA-AHMGsLRRFIKV--G------

CAC80544.1 YGPTGAFNAKPFEGsRSWA--GARPELRAIGHDSNGVA-AHMGLLRRFIKV--G------

ACV52960.1 YGPTGvFNAqPFEGsRSWA--GARPELRAIaYDSNGVA-AHMGcLRRFIKV--d------

ZP_03519615.1 YGPTGvFNAKPFsGsqSWA--GARPELRAIGYDSsGVA-AHMGLLRRFIKV--d------

ABO64758.1 YGPTGeFNAKPFEGsRSWA--GARPELRAIaYDSrGVA-AHMGLLRRFIKV--G------

CAC82838.1 YGPTGAFNAKPFEGsRSWA--GARPELRAIaYDShGIA-AHMGLLRRFIKV--G------

ABO64759.1 YGPTGAFNAKPFEGsRSWA--GARPELRAIaYDShGIA-AHMGLLRRFIKV--G------

CAC82889.1 YGPTGAFNAqPFEGnRSWA--GARPELRAIGYDArGVA-AHiGLLRRFIKI--d------

CAJ41271.1 YGPTGAFNAqPFEGnRSWA--GARPELRAIGYDArGVA-AHiGLLRRFIKI--d------

CAC82881.1 YGPTGAFNAqPFEGnRSWA--GARPEVRvIGrDAhGVA-AHvGLLRRFIKI--G------

ACA97556.1 YGPTGAFNAqPFQGsRSWA--GARPEVRvIGHDArGVA-AHvGLLRRFIKV--G------

NP_768665.1 YGPTGAFNAqPFErsRSWA--GARPELRvIGYDArGVA-AHiGLLRRFIKV--G------

WP_015930557.1 YnPsGtFNARPFEGGRSWA--GARPEVRAIGYDSrGVA-AHiaaLRRFIKV--G------

CAC42487.1 YGPTGAFNAKPFEGnhSWA--GARPEfRAIGYDArGVA-AHiGaLRRFIKV--G------

ABX80632.1 YGPTGAFNAKPFEGnhSWA--GARPEVRAIGYDArGVA-AHiGILRRFIKV--G------

WP_013533527.1 YGrnGAFgAKPFEGsRSWA--GARPELRlIasDpEGVA-AHiGILRRFIKV--G------

CAC82870.1 YGrTGAFNAePFEGGRSWA--GARPEfRAIGYDAhGVA-AHiGMLRRFIKV--G------

CAC82868.1 YGrTGAFNAePFEGGRSWA--GARPEfRAIGYDAhGVA-AHiGMLRRFIKV--G------

ACT34095.1 YGrTnAsNAKPFEGGRSWA--GARPEVRAIGYDAhGIA-AHiGILRRFIKV--G------

ACT34108.1 YGranAsNAKPFEGGRSWA--GARPEVRAIGYDAhGIA-AHiGILRRFIKV--G------

YP_007685003.1 fpdfaAg----yvGaRSWA--GAqPELRvlvHDgDeIv-AHaGLrRvFael--GDGQGDP

WP_012037425.1 fpdfaAg----yaGaRSWA--GAqPELRilvHDgDelv-AHaGIrRmFvsa--GnGQGDP

JGI:640540037 fpdfaAg----yaGaRSWA--GAqPELRilvHDgDelv-AHaGIrRmFvsa--GnGQGDP

YP_001710907.1 fpdfreg----yaGaRSWA--GAqPELRilvHDgDelm-AHaGIrRlFvef--GDGQGDP

JGI:2553980777 fpdwshw----FlGsRSWs--GmqPErRvlarDADGVvLAHvGvrRmFIsV--G------

JGI:2596277042 fpdwshw----yvGGRSWs--GmqPErRvlarDdhGVvLAHvGIrRmFItV--G------

JGI:2553420270 -----------yvGGRSWs--GmqPErRvlarDpDGVvLAHvGIrRmFItV--G------

EYT63040.1 fpdwshw----yvGGRSWs--GmqPErRvlaHDADGVvLAHvGIrRmFvtV--G------

WP_027465027.1 fpdwshw----yvGsRSWs--GmqPErRvlaHDADGVvLAHvGIrRmyIsV--G------

WP_016698138.1 fvsTshq----FrdaRSWA--GARPELRlVacagDtVv-AHaavLRRFvrV--G------

JGI:2585523411 fasTshh----FrdaRSWA--GARPELRlgarvgEaVv-AHaavLRRFvrV--G------

JGI:2599913978 fpaysel----yrGaRSWA--fARPEVRiIGelgDhVv-AHLGvLRRFlrVlDt------

YP_003135251.1 fpaysel----yrGaRSWA--fARPEVRiIGelgDhVv-AHLGvLRRFlrVlDt------

JGI:2574491345 fpyTvdq----FrdGRSWA--GARPErRvIGWadgrpv-AHtGLLRRFlrV--G------

WP_007498427.1 fpyTaeq----FvhGRSWv--GARPErRvtaWrgDrpv-AHvGILRRFlrI--G------

JGI:2574392503 Yprhvel----FrdGctWs--GARPELRvVGsagDrpv-AHLGLvRRFlrVqqt------

JGI:2515227178 fprTGA-----FgqGRSWv--GARPErRlVarrdgrpv-AHaaLvRRFlrapDe------

WP_005476203.1 tvDqLVAdcGLlgVRPDLq--rsrVsYdLlvavrsavmELkVPFaFGamReeLrrlvlR-

WP_012172341.1 GeqIaVAELGLYgVRrDLE--GLGIGHStla-MlPVLKaLGVPFaFGcfRNeLriHFQR-

CAC82897.1 GeqIaVAELGLYgVRrDLE--GLGIGHStla-MlPVLKaLGVPFaFGcfRNeLriHFQR-

WP_013873450.1. aiDqLVAEvGLYAVRPDLqrsGIGyqsginv-MiPtLlDLkVPFGFGTVRHeLreHvEk-

AAK50870.1 niDvLVAELGLYAiRPDLE--GrwlaNSLRv-MHPaLKQLsVPFGFGTVRsALerHvgRL

WP_012555955.1 dtDLLVAELGLYgVRPDLE--GLGIaHSiRa-LaPaLQDLaVPFaFGTVRHAMrnHvER-

WP_028214790.1 ttDvLVAELGLYgVRPDLE--GLGIsqSiRa-MYPVLQELrVPFaFGTVRYALknHvtR-

ACC17805.1 ttDvLVAELGLYgVRPDLE--GLGIsqSiRa-MYPVLQELrVPFaFGTVRYALknHvtR-

CBM43078.1 ttDvLVAELGLYgVRPDLx--GLGIsqSiRa-MYPVLQELrVPFaFGTVRYALknHvtR-

CAC80544.1 evDLLVAELGLYgVRPDLE--GLGIsHSLRv-MYPVLQQLrVPFGFGaVRHALqrHvER-

ACV52960.1 GvDLLVAELGLYgVRPDLE--GLGIaHSiRf-MiPtLQELGVPFaFGTVRHALqkHiER-

ZP_03519615.1 GvDLLVAELGLYgVRPDLE--GLGIsHSiha-LsPtLQELqVPFaFGTVRpeLrkHvER-

ABO64758.1 evDqLVAELGLYgVRPDLE--GLGIsHSihv-MlPVLQELGVPFaFGTVRHALrkHiER-

CAC82838.1 evDqLVAELGLYgVRPDLE--GLGIsHSihv-MlPVLQELGVPFaFGTVRHALrkHvER-

ABO64759.1 evDqLVAELGLYgVRPDLE--GLGIsHSihv-MlPVLQELGVPFaFGTVRHALrkHvER-

CAC82889.1 GvDLLVAELGLYAVRPDLE--GLGIsHSmRv-MYPaLQQLGVPFGFGTVRpALekHltRL

CAJ41271.1 GvDLLVAELGLYAVRPDLE--GLGIsHSmRv-MYPaLRQLGVPFGFGTVRpALekHltRL

CAC82881.1 avDLLVAELGLYAVRPDLE--GLGIsHALRv-MYPILhELGVPFGFGTVRpALekHltRL

ACA97556.1 GvDLLVAELGLYAVRPDLE--GLGIsHAmRv-MYPILQDLGVPFGFGTVRsALekHltRL

NP_768665.1 evDLpVAELGLYAVRPDLE--GhGIGHAmRv-MYPaLQELGVPFGFGaVRsALekHltRL

WP_015930557.1 avDLLVAELGLYAVRPDLE--GLrIsHSmlv-MYPaLKELGVPFGFGTVRHALqkHltRL

CAC42487.1 avDLLVAELGLYAVRPDLE--GLGIpqlmRv-MYPVLQELGVPFGFGTVRHALrqHiaRL

ABX80632.1 avDLLVAELGLYAVRPDLE--GLGIpHlmRv-MYPVLQELGVPFGFGTVRHAMrqHiaRL

WP_013533527.1 dvDvLVAELGLYgVRqDLE--kLGIsfSmRa-vYPVLQQmGVPFGFGTVRHAMrnHvER-

CAC82870.1 evDLiVAELGLYAVRPDLE--GLGIGfSmRv-aYPVLhQLGVPFaFGTVRHALrnHvER-

CAC82868.1 evDLiVAELGLYAVRPDLE--GLGIGfSmRv-aYPVLhQLGVPFaFGTVRHALrnHvER-

ACT34095.1 evDLLVAELGLYgVRPDLE--GLGIsfSmRf-vYPVLQQLGVPFaFGTVRHALrnHvER-

ACT34108.1 evDLLVAELGLYgVRsDLE--GLGIsfSmQf-vYPVLQQLGVPFaFGTVRHALrnHvER-

YP_007685003.1 adDLLmgstGmvAVhPerq--GqGIGtlLadgirgaLarLaVPFGlletgaettgyYER-

WP_012037425.1 adDLLmgstGmvAVhPerq--GqGlGtlLadgirgaLarLaVPFGlletgeStsgyYaR-

JGI:640540037 adDLLmgstGmvAVhPerq--GqGlGtlLadgirgaLarLaVPFGlletgeStsgyYaR-

YP_001710907.1 adDLLmgstGmvAVhPerq--GqGlGtlLadgirgaLarLaVPFGlletgdettgyYaR-

JGI:2553980777 seDvLVgdtGLvAVsPrLq--GaGmGrdLleRarsVLEgLdVPFGFlgtgedrvpfYak-

JGI:2596277042 GrDvLVgdtGLvAVsPrLq--GtGrGreLmeRaHaVLEgLrVPyGFlgagenrvpfYak-

JGI:2553420270 GrDvLVgdtGLvAVsPrLq--GsGrGreLmeRtHaVLdgLaVPyGFlgagenripfYak-

EYT63040.1 GqDvLVgdtGLvAVsPrLq--GtGVGreLmtRtHaVLdgLqVPyGFlgagedripfYak-

WP_027465027.1 GqDLLVgdtGLvAVsPrLq--GtGVGreLmtRtaaVLEgLrVPFGFlgagedripfYak-

WP_016698138.1 raDqLVgELGLvAVhPDaq--GnGlGaqLlaRveerLRDLGVPFGllntdqrtaafYaR-

JGI:2585523411 etDqLVAEvGLvAVhPDaq--GnGlGaqLlaRvedrLRlLGVPFGllntdrttatfYaR-

JGI:2599913978 GasvLVgEvGLvmVhPDfr--GrGlGrrLldeatdVLtgfdlPFGFlTcRrgIvpfYER-

YP_003135251.1 GasvLVgEvGLvmVhPDfr--GrGlGrrLldeatdVLtgfdlPFGFlTcRrgIvpfYER-

JGI:2574491345 GqeqLVgEvGLvAVhPDLq--GIGaGrALadRtrqaLasLelPFGylncmdSvlgyYts-

WP_007498427.1 srDqLVgEvGLvAVargLq--GtGVGrlLaehvrerLtrLGVPFcylncqsSvlgyYQs-

JGI:2574392503 GasLLVgdvGLvAVdPDLq--GrGVGrALldhtlrtmtELalPFGFlTcRpevvpfYrs-

JGI:2515227178 GrsvLVgdvGLvAVdParq--GtGlGaALlaevaatLaDLdlPFGFlTcgpevaafYrR-

WP_005476203.1 anat----ILpdVqVRATs---qwDpaemvIEDhfVyIdPi-mQpveEWPpGkLIDRNGP

WP_012172341.1 fcRnGkgaIvdnVnIkSTqPDIypDlPPTKIEkkaavILPl-teTLdrWPeGvdIeRNGP

CAC82897.1 fcRnGkgaIvdnVnIkSTqPDIypDlPPTKIEkkaavILPl-teTLdrWPeGvdIeRNGP

WP_013873450.1. -asaaLm-IvSGVlVRSTn---lwDkskTcIEDllVlVIPl-eRAvdEWPAGeLIDRNGP

AAK50870.1 lGRpGLATILhGVkVRSTlsEVyqnlsaTRVdeplVlVfPi-vspLSkWPpGatIeRNGP

WP_012555955.1 fcRdGIAnILTGVrVRSTlPDaqlDmPaTRtEDvlLlVfPi-GRTMSEWPpGSLIeRNGa

WP_028214790.1 lcRhGIgTVvSGVrVRSTlPnVhlDlPaTRVEDvlavIMPi-dRAMSEWPhGTeIeRNGP

ACC17805.1 lcRhGIgTVvSGVrVRSTlPnVhlDlPaTRVEDvlavIMPi-dRAMSEWPhGTeIeRNGP

CBM43078.1 lcRhGIgTVvSGVrVRSTlPnVhlDlPaTRVEDvlavIMPi-dRAMSEWPhGTeIeRNGP

CAC80544.1 fGRhlpATVLSGIrVRSTlPDarlDlPPTRMEDvlVvVLPV-GsAMSDWPtaTLIDRNGP

ACV52960.1 fGRhsqlTVLSGIrVRSTlPDarlDkPPTRIEDalViVLtl-aRSMSDWPtGnfIDRNGP

ZP_03519615.1 fGRrGpvTVLSGIsVRSTlPharvDlPPTRVEDplViVLPV-GRSMSDWPtGatIDRNGP

ABO64758.1 faRyGLlTVMSGVqVRfTlPEariDlPPTRVEDplViVLPV-GRSiSDWPtGaIIDRNGP

CAC82838.1 faRyGLvTILSGIhVRfTlPEarlDkPPTRtEDalViVLPV-GQSMSDWPAGTtIDRNGP

ABO64759.1 faRyGLvTILSGIhVRfTlPEarlDkPPTRtEDalViVLPV-GQSMSDWPAGTtIDRNGP

CAC82889.1 vGRqGLATLMSGVrVRSTqPDVypnlsPiRIEDvlVvVfPl-eRpigEWPAGTIIDRNGP

CAJ41271.1 vGRqGLATLMSGVrVRSTqPDVypnlsPiRIEDvlVvVfPl-eRSLgEWPAGTIIDRNGP

CAC82881.1 vqRqGLATLMSGlrVRSThPDVypnlsPiRIEeviVvVfPV-qsAiSEWPAGaMIDRNGP

ACA97556.1 veRqGLATLMhGIrVRSTqPDVypnlsPTRIEDvvVvVfPV-GsSiSEWPAGTVIDRNGP

NP_768665.1 veRqGLATLMrGIrVRSTlPDVypnlsPTRIEDviVvVfPV-GRSiSEWPAGTVIDRNGP

WP_015930557.1 lGKaGLATIvSGVrVRSTlrDMrlDmPPTRVEDllIlVfPi-GRpMSDWPAGTIIDRNGP

CAC42487.1 lGRpGLATIvSGVrVRSTlrEVhlDtPPTRIEDvlIvVLPi-GRSMSDWPtGTIIDRNGP

ABX80632.1 lGRhGLATIvSGVrVRSTlrEVhlDtPPTRIEDvlVvVLPi-GRSMSEWPtGTIIDRNGP

WP_013533527.1 ycReGIATIvpGVrVRSsranVhhDlPsTRLdDvlVlVsPi-GRSMdEWPSGTLIDRNGP

CAC82870.1 fcRaGLAnIvSGVrVRSTrPDVhpDlPPTRLEDvlVlVsPi-GRSMdEWPSGTLIDRNGP

CAC82868.1 fcRaGLAnIvSGVrVRSTrPDVhpDlPPTRLEDvlVlVsPi-GRSMdEWPSGTLIDRNGP

ACT34095.1 fcRgGLATMLSGIpVRSThPEVypDlPPTRLEDvlVlVtPi-GRSMSEWPSGTLIDRNGP

ACT34108.1 fcRgGLATMLSGIpVRSThPEVypDlPPTRLEDvlVlVtPi-GRSMSEWPSGTLIDRNGP

YP_007685003.1 hGwi----pLpdrtghyngftLl-gaaevvhqDhgwMILPV-tspadafPAGa-lhvNGq

WP_012037425.1 hGwl----pLdGrtghyngftLl-gaagvvhqDhgwMILPV-tapadafPAGd-lhvNGq

JGI:640540037 hGwl----pLdGrtghyngftLl-gaagvvhqDhgwMILPV-tapadafPAGd-lhvNGq

YP_001710907.1 hGwi----pLpGrtghyngftLl-gaagvvhqDhgwMmLPV-taAadafPAGd-lhvNGq

JGI:2553980777 lGwh----hfdeVvtdyTaftae-gagmpmtEqggwMVLPV-aspLeDWPAGp-vrlNaq

JGI:2596277042 lGwh----VfddVvadyTaftae-gaglpmtdqggwMVLPV-adrLeDWPAGp-IalNGq

JGI:2553420270 lGwh----efpeVvgtfsaftae-gagiTntEqggwMVrPV-aaALeDWPAGp-IwlNGq

EYT63040.1 lGwh----efpeavgtfsaftae-gagvsntEqggwMaLPV-aaqLeDWPtGp-IwlNGq

WP_027465027.1 lGwh----efpeavgtfsaftae-gagvsntEqggwMaLPV-aaqLeDWPdGp-IwlNGq

WP_016698138.1 sGwc----aLTGVasRwadvDypwqnadlR---dpLlVLPV-aapLTrWPgGgVvDRNGg

JGI:2585523411 sGws----VLTGVasRwadPDypwrhadlR---dpLlVLPV-aapLTrWPgGdVvDRNGg

JGI:2599913978 cGwr-------attsRSTmiDgdqrpgP---dpavVMVLPV-taewpsWPdGhVvDRNGq

YP_003135251.1 cGwr-------attsRSTmiDgdqrpgP---dpavVMVLPV-taewpsWPdGhVvDRNGq

JGI:2574491345 aGwi----evSGadtRhyePD---DeltavVttarplILPV-aadavsWPAGprIeRdGt

WP_007498427.1 vGRl----rLeatatRhfeaD---DeltavttsahpMILPVtaRgrADWPAGpLIeRdGt

JGI:2574392503 gGwq----rLdGqitRmidnDhrpE-----VyDgpaMaLPV-rapMTDWPhGltvDRNGl

JGI:2515227178 gGwqpadqpLhaIdVhh----------hvetdrangMlLPV-rQArADWPrGrLv-RdGe

WP_005476203.1 pL-

WP_012172341.1 EL-

CAC82897.1 EL-

WP_013873450.1. EL-

AAK50870.1 EL-

WP_012555955.1 EL-

WP_028214790.1 EL-

ACC17805.1 EL-

CBM43078.1 d--

CAC80544.1 EL-

ACV52960.1 EL-

ZP_03519615.1 EL-

ABO64758.1 EL-

CAC82838.1 EL-

ABO64759.1 EL-

CAC82889.1 EL-

CAJ41271.1 EL-

CAC82881.1 EL-

ACA97556.1 EL-

NP_768665.1 EL-

WP_015930557.1 EL-

CAC42487.1 EL-

ABX80632.1 EL-

WP_013533527.1 EL-

CAC82870.1 EL-

CAC82868.1 EL-

ACT34095.1 EL-

ACT34108.1 EL-

YP_007685003.1 lv-

WP_012037425.1 lv-

JGI:640540037 lv-

YP_001710907.1 lv-

JGI:2553980777 qve

JGI:2596277042 qv-

JGI:2553420270 Ev-

EYT63040.1 qv-

WP_027465027.1 qv-

WP_016698138.1 vL-

JGI:2585523411 vL-

JGI:2599913978 kv-

YP_003135251.1 kv-

JGI:2574491345 EL-

WP_007498427.1 EL-

JGI:2574392503 Ev-

JGI:2515227178 EL-

NodB alignment

WP_013872874.1 --------------mtylpgveeveeaaswwptrpagrvgragnvvgragnagvAgfara

WP_020462976.1 ----------------mvrsgtgrwetgrwaaggavmalsalayglpsla----------

YP_711985.1 ---------------------malsatagltyglpslatlr-------------------

WP_013872989.1 -----------------------------mpggaatavahg-------------------

WP_028230172.1 ------msSpslpsscftpaavrrwkpspficgaaalhagaaaalavqpgawpwAvgsva

WP_028211220.1 ------msSpslpsscftpaavrrwkpspficgaaalhagaaaalavqpgawpwAvgsva

WP_013871639.1 ---------------mrhrrdahrtprahwlllalfigtlasmlgiqgfarhgtgpartp

YP_003638438.1 ----------------------mdpsapdviryvatapaap-------------------

YP_004404326.1 ----------------------mtarhsssmpgvpacpeat-------------------

YP_004242916.1 -----------------msraksvknkaagagfmmterkamklpihrpvslrkaAfpvaa

YP_290844.1 -------------------mttvprrsplrkrllvalcalglaftsaata----------

YP003833384 --------------------mrpralsalalglvllvtgcgeagkspekaaappSasasp

YP_004402816.1 ------------------mrprlllacltgltlllsgcgagsaepnptpadappSaalpd

YP_004082445.1 MRgstlraAglvtvvlagllgsafalgrslvpdphpsaagvattlngprygeqppstdfp

EWM67253.1 MRgptlratglvtvvlagllgsafalgrslvpehppsnagattalngpryadqppstdfp

YP_001157155.1 MqtkgggptrmgglrqftpatrslaiaavvviallgsafaigrgiaptgapvtsSattph

YP0_04408115.1 -------mlgiitlvvtavlgsayllgrslvpdpprhdsavtatgadhpeyadqSaeadp

YP_003115329.1 --mllggcAstgkpvavrpagattptaggsprsrpttggapfpgqspaspgtspAggdts

YP_715094.1 -------------------mlltaaepaqaigeasraghnavrsagqqtaarsgAargsg

YP_004014355.1 ---------------------msrlrrgrwvsdlapvearerdagrsrrrltawAlvgai

YP_001509470.1 --------------------miarpssgdgfgarggceagggaggpsrrrllgwSiagaa

YP_004332108.1 -----------------mtthaqirpvtpaapgrprlnaasavltalvfglvtfltapds

WP_012172340.1 ---------------------------msvlgqaaritqnq-------------------

AAB51163.1 ---------------------------msvlgqaaritqnq-------------------

ZP_07980118.1 ------------------ppraqrarapeepdakppepapgprttppap-----------

WP_013873451.1 -----------------------------mtgccpsstfsg-------------------

AAK50871.1 -----------------------msvgvkcigirdagnesv-------------------

CAD43932.1 ---------------maekvhsepserlswpteirerdakv-------------------

YP_001796209.1 ---------------maekvhsepserlswprkirerdakv-------------------

WP_013874294.1 -----------------mpdgqtvastttgtttcpqayddrrtearrtaaalrahagtli

WP_037627918.1 ------------------mdtvttagpqdgptdrpsssace-------------------

YP_003610199.1 ------------------------mnhpdyltevrsnsdca-------------------

ACT34139.1 ------------------------mihldcisevpsewaat-------------------

ACT34142.1 ------------------------mihldyisevpserast-------------------

ACT34099.1 ------------------------mkhldyisempsewast-------------------

ZP_03526066.1 ------------------------mthldcscevhgerddg-------------------

AAD11394.1 ------------------------------------------------------------

CAA68620.1 ------------------------mkrpayigevpvnhtsg-------------------

AAY26501.1 ------------------------------------------------------------

ABD67418.1 ------------------------mkhldyiyevpsnddyg-------------------

ABG91001.1 ------------------------------------------------------------

ABD67421.1 ------------------------mkhldyiyevprngdcg-------------------

AAL93157.1 ------------------------------------------------------------

YP_002499211.1 ------------------------mrrlnylselrsecadg-------------------

AAL93156.1 ------------------------------------------------------------

CAC42488.1 --------------------------mrhclsearsecadt-------------------

WP_013872874.1 Aagagaaallthavpsva----------------------------------------tl

WP_020462976.1 ----------------------------------------------------------tf

YP_711985.1 ------------------------------------------------------------

WP_013872989.1 ------------------------------------------------------------

WP_028230172.1 AshlaltaaglwP-----------------------------------------------

WP_028211220.1 AshlaltaaglwP-----------------------------------------------

WP_013871639.1 ApdgpveetavsPgDgl-----------------------------------------gg

YP_003638438.1 ------------------------------------------------------------

YP_004404326.1 ------------------------------------------------------------

YP_004242916.1 AvlalsasvpaglvgAT-----------------------------------------aa

YP_290844.1 ------------------------------------------------------------

YP003833384 SptpvtkPsptpttpAkP----------------------------------------kl

YP_004402816.1 ppappsptaspkP---------------------------------------------kl

YP_004082445.1 ggtasprPspdaapDA------------------------------------------ap

EWM67253.1 ggratagPspdaapDA------------------------------------------ap

YP_001157155.1 ptdqpaaPetpaPdDsSataeAhdggtvavpePtavdesSpAaAeptaP---------ep

YP0_04408115.1 ghpsaspsnppsPsDgT-------------------------------------------

YP_003115329.1 tapgdshsngsaPpDsSPppsAt-----------------------------------gq

YP_715094.1 vaastgvttttvaap---------------------------------------------

YP_004014355.1 glasagvaeaagPalATepppAaapaparagaPapttapAaAhApaavPaqgsagasgaa

YP_001509470.1 gltalgavdpalaapAa-----------------------------------------gr

YP_004332108.1 pfravtpPppppPapvv-------------------------------------------

WP_012172340.1 ------------------------------------------------------------

AAB51163.1 ------------------------------------------------------------

ZP_07980118.1 ------------------------------------------------------------

WP_013873451.1 ------------------------------------------------------------

AAK50871.1 ------------------------------------------------------------

CAD43932.1 ------------------------------------------------------------

YP_001796209.1 ------------------------------------------------------------

WP_013874294.1 AtatmtirsrapvsisTPlr--------------------------------------le

WP_037627918.1 ------------------------------------------------------------

YP_003610199.1 ------------------------------------------------------------

ACT34139.1 ------------------------------------------------------------

ACT34142.1 ------------------------------------------------------------

ACT34099.1 ------------------------------------------------------------

ZP_03526066.1 ------------------------------------------------------------

AAD11394.1 ------------------------------------------------------------

CAA68620.1 ------------------------------------------------------------

AAY26501.1 ------------------------------------------------------------

ABD67418.1 ------------------------------------------------------------

ABG91001.1 ------------------------------------------------------------

ABD67421.1 ------------------------------------------------------------

AAL93157.1 ------------------------------------------------------------

YP_002499211.1 ------------------------------------------------------------

AAL93156.1 ------------------------------------------------------------

CAC42488.1 ------------------------------------------------------------

WP_013872874.1 rrlrtqlmpalagigapdhVALTFDDGPDPa-----sTPlfLDVLAEldlrATFFVLGtm

WP_020462976.1 rrlrtrvtpglagvgrpdhVALTFDDGPDPa-----sTPrfLeVLdaleIrsTFFVLGgm

YP_711985.1 -rlrlrvtpalagvgagdhVALTFDDGPDPa-----sTPQfLDVLAEldVrsTFFVLGsl

WP_013872989.1 -------------prdrpqVALTFhlGPheadqdlslahrlLteaAEllVsiTvFaVGqW

WP_028230172.1 rsallgpnwtrlpepvgRriALTiDDGPDPe-----vTPrVLDlLdrfdarATFFcIGel

WP_028211220.1 rsallgpnwtrlpepvgRriALTiDDGPDPe-----vTPrVLDlLdrfdarATFFcIGel

WP_013871639.1 pvldlsgpapqgrglpaRtVALTFDDGPDPr-----wTPaILDVLrrHraqATFFVVGsr

YP_003638438.1 ------------------tVALTFDDGPnPp-----dTvalLDlLAregVrAvFclVGvq

YP_004404326.1 -------------igtdRvlcLTFDDGPhPv-----hTPrlLDVLAaHdVPAvFFlqGdq

YP_004242916.1 qadeatpaivestrhegKyasLTFDDGPDPv-----sTPklLaVLekHhVkATFclwGdH

YP_290844.1 -haqvtpdivtttgqpgRtVALTFDDGPnPn-----dTPalLsVLrkHqVkAvFclwGeH

YP003833384 rplpkklpaglhrasgsngVALTFDDGPDPr-----yTPQILaqLraahVkATFcVVGkq

YP_004402816.1 rplpaklpaglvrttggKkVALTFDDGPDPa-----wTPkVLDlLkaakVkATFcVVGtq

YP_004082445.1 vepggdgpygalvatgssqVALTFDDGPDPr-----wTPQVLalLAQYgVrATFcVVGen

EWM67253.1 vepggdgpygslvvtgssrVALTFDDGPDPr-----wTPQVLalLAQYgVkATFcVVGen

YP_001157155.1 tassdyydegtqrttgtstVALTFDDGPnPq-----yTPQILtsLrEYgVtATFcVtGkn

YP0_04408115.1 --dpnfgpmgtrlstgtneVALTFDDGPnPd-----yTPQILaILrEYhVtATFcVVGqn

YP_003115329.1 dppppaghpqfyvhegdKaiALTiDDGPssk-----yTPQVLalLAQYkIPATFcmIGqn

YP_715094.1 -kapayrihdlrpdaprdsVALTiDDGPhPv-----wTPrILDVLrvnrVsATFsVVGaq

YP_004014355.1 rqrpaytvhrvlpnapanaiALTFDDGPDPt-----wTPQVLalLrQYdVrATFcIVGrq

YP_001509470.1 rsgavsrvrgarpaapanaiALTiDDGPhPv-----wTPrILeVLrgngVrATFFVIGvq

YP_004332108.1 vqavpkvltevrgqpgaRtVALTFDDGPDPt-----wTPrILDVLrsHgavATFcqIGnv

WP_012172340.1 -----------------ssiyiTFDDGPhPs-----vTPaVceILrEHsalATFFqIGrF

AAB51163.1 -----------------ssiyiTFDDGPhPs-----vTPaVceILrEHsaltaFFqIGrF

ZP_07980118.1 -pppvrtrpfarlpraghalALTFDDGPDPr-----wTPEVLaVLAaHdVrATFFVcGrq

WP_013873451.1 ------------gqsgtRriALTFDDGPDPy-----yTPrILDlLAEHkVaATFcVLGtY

AAK50871.1 ---------------ddRcVyLTFDDGPDrr-----nTsEILsVLsEYnVPATFcVLGvY

CAD43932.1 ---------------kpRkVfLTFDDGPnPi-----wTPkILDILrQfhVPgTFFVLGaY

YP_001796209.1 ---------------kpRkVfLTFDDGPnPt-----wTPkILDILrQfhVPgTFFVLGaY

WP_013874294.1 sepgqidsrkirieegsRdifLTFDDePnPf-----cTPQVLDVLAEHrVvATFcVIGeY

WP_037627918.1 ---------vrgaddgdRciyLTFDDGPnlf-----cTPQILDVLAEHralATFcVIGeY

YP_003610199.1 --------------dlrpkVyLTFDDGPhPv-----wTPkILDILeyeqatATFcVIGaY

ACT34139.1 ---------------grRsVyLTFDDGPnPf-----fTPQILDVLAQnrVPATFFVIGtY

ACT34142.1 --------------pgrRtVyLTFDDGPnPf-----fTPQILDVLAQdrVPATFFVIGtY

ACT34099.1 ---------------grRsVyLTFDDGPnPf-----fTPQILDVLAQnrVPATFFVIGtY

ZP_03526066.1 --------------tgshsVyLTFDDGPhPf-----cTPEILDILAEHrVPATFFVIGeF

AAD11394.1 -----------------------------------------LhlLAEHrVPATFFVLGtY

CAA68620.1 --------------qeaRcVyLTFDDGPnPf-----cTPQILDVLAEHrVPATFFaIGsY

AAY26501.1 -------------------------------------TPEVLDVLAQHqVPATFFVIGtY

ABD67418.1 --------------tedRsiyLTFDDGPnPh-----cTPEILDVLAEYgVPATFFVIGtY

ABG91001.1 --------------------------------------------------PATFFVIGtY

ABD67421.1 --------------tedRsiyLTFDDGPnPh-----cTPEILDVLAEYgVPATFFVIGtY

AAL93157.1 -----------------------------Pl-----cTPdILDlLAErrIqATFFVIGtY

YP_002499211.1 --------------rghhsVyLTFDDGPnPr-----cTPdILDVLAEHrVPATFcVIGaY

AAL93156.1 -------------------------------------------VLAEHraPATFFVIGaY

CAC42488.1 --------------ngrRdVyLTFDDGPnPl-----cTPdVLDVLAQHrVPATFcVIGaY

WP_013872874.1 leraPhLaeRmVdaGHELAvHgwdHrp-mllrGPrstyrqlyRtrelIttltgrpp-RFv

WP_020462976.1 leraPgLaRemteaGHELAvHgwdHrp-mllrGPastydqlaRtrdlIaettgrAp-aYv

YP_711985.1 lersPsLaRemVAaGHELAvHgwdHrp-mllrGPrstRdqlvRtrdlvaevtgrtp-RFv

WP_013872989.1 ldEHrDLVpvIlAaGnELANHTysHPv-LTalpadlVaaEIigcrdvlarlaPaqg-RYF

WP_028230172.1 ArrHPQLVeaIVArGHaveNHsehHrltfSlfGPramKrEIaagqrtlteiagvAp-RFF

WP_028211220.1 ArryPQLVeaIVArGHaveNHsehHrltfSlfGPramKrEIaaAqrtlteiagvAp-RFF

WP_013871639.1 vlEHPELVRRIrqDGHQLgiHTfsHcD-LTavptwrrRiEVslsqsAlaaAtgats-sll

YP_003638438.1 veQHPDLVRRVVdDGHvLANHswqHdD-LAelpaaaVRadlqRtldAIhavvPgvpVpFF

YP_004404326.1 AAsHPDLVRRIVAaGHaLgNHsMhHdD-mSdwtPgrIasdlletNavIrrAvPqAplpYF

YP_004242916.1 vkQHPDiVRqIaAaGHlLcNHTMhHdN-mgawsaeaIKadlletsaAIreAvPdAkIdYF

YP_290844.1 vrQyPaiaRqIaeEGHiLcNHsMrHdD-mgnwsaaQIRadleatNqAIreAvPnAdIpYF

YP003833384 AkryPELVaRIVrEGHQLcNHswhHdvNLgrrsaaEIRsdleRtNeAIhaAaPkApItwF

YP_004402816.1 vrKHPELVRRIarEGHQLcNHswnHdlNLArrpvaEIRadltRtNreIrrAvedAkVpYY

YP_004082445.1 veaHPaLVRsIVAEGHtLcNHswnHdvNLgkraPatIRadllRtNdAIlaAaPdAqIaYY

EWM67253.1 AeaHPDLIRsIVAEGHtLcNHsweHdvDLgkrsPatIRadIlRtNdAIlaAaPgArIaYY

YP_001157155.1 ASayPELIqaIVADGHtLcNHTwnHdiaLgsrsPdEIRadliRtsdAIhaAvPdApIaYY

YP0_04408115.1 AqayPwLVqqIVdEGHtLcNHswnHdvSLggrsPdrIRddllRtNaAIhaAaPdApVtwY

YP_003115329.1 AAQHasLVaeVsAaGHlvANHTwTHPN-LAkmseaQVtaEIeRtNdAItkggarqpV-lF

YP_715094.1 AvanPELVRRIVAEGHsLcNHTMTHPqpfgartPeQIRaEmtRAqsAvvdAgaepp-RlF

YP_004014355.1 ArayPDLVRRIVAEGHaicNHsMTHPlpfShrsaaaIdaEIggAqsAItaAagtAp-RlF

YP_001509470.1 AkaHPELVRRVlAEGHtvgNHsLdHPtpfgagsaatVarEIsaAqaiItaAggiAp-RYF

YP_004332108.1 mAaHPEtVamVaAaGmrmcsHsrTHdESLStraestVvaEIvdvrdraehv-PgAdVeYF

WP_012172340.1 AkEyPsisRqcqlDGHaigNHTfdHPN-LqdrageEVeyqIssAqkclehiCgrgfVRhF

AAB51163.1 tkEyPsisRqcqlDGHaigNHTfdHPN-LqdrageEVeyqIssAqkclehiCgrgfVRhF

ZP_07980118.1 AAKHPaLlRRIteaGHlvgNHTwdHrr-LTglGraaVeeqIaRtsetvrrAtgrAp-awF

WP_013873451.1 AAaHPDiIsRIaAEsHlLAcHsMTHaD-LScCGeqQtRwEImaAsrtlqavaPtArVqYl

AAK50871.1 AlrerDLIRRmasEGHEivNHsMsHaD-LSkCdiaElQyqIdQAcfAIksvCPggvIRYF

CAD43932.1 AAEHPELIRRmIsDGHEvANHTMTHlD-LSqCnrdvlRrEIldtNviItkAsPglaVRFl

YP_001796209.1 AAEHPELIRRmIsDGHEvANHTMTHlD-LSqCnrdvlRrEIldtNviItkAsPglaVRFl

WP_013874294.1 AAKHPELIRRIatEGHgLANHTMTHrD-LSrCePgEVRrEIsdANkvIrtvCPqAcVhYl

WP_037627918.1 AAgHPELIRRIVAEGHgLAsHTMTHrD-LShCaPeEIQqEIsRAsdvItaisPqtplRhl

YP_003610199.1 AAEyPELIkRIaAEGHELANHTvTHPD-LSkCePdlVRrEIreAdalIketCPaAkVtYF

ACT34139.1 AvEHPDLIqRmIAEGHEvgNHTMTHPD-LckgsiaEVhrEVfeANgAImmACPqAsVRFl

ACT34142.1 AvEHPDLVqRmIAEGHEvgNHTMTHPD-LckCGfgEVRrEIfeANgAIsmACPqAsVRFl

ACT34099.1 AvEHPDLIqRmIAEGHEvgNHTMTHPD-LckCsfgEVRrEIfeANgAImmACPqAsVRFi

ZP_03526066.1 lADqskLIqRmIAEGHEvANHTMTHPD-LSdCePdEVQrqIletNrAIkmAsPqAvVRhi

AAD11394.1 vkDHPDLVRRaaAEGHlvANHTMTHPD-LTvCGsevIerElneANkAIvsACPqAtVqhm

CAA68620.1 vkDHPELIRRLVAEGHDvANHTMTHPD-LAtCdPkDVKrEIdeAhqAIvsACPqAlVRhl

AAY26501.1 vtEHPELIRRtIAEGHEiANHTMTHPD-LSkCGPsElhdEVltAseAIrlACPqAapRhm

ABD67418.1 AknqPELIRRIVAEGHEvANHTMTHPD-LStCGPhEVQrEIieAsetIisACPqAvVRhi

ABG91001.1 AksqPELIRRIVAEGHEvANHTMTHPD-LStCGPhEVerEIveAseAIiaACPqAaVRhi

ABD67421.1 AknrPELIRRIVAEGHEvANHTMTHPD-LStCGPhEVerEIveAseAIiaACPqAaVRhi

AAL93157.1 AAQqPELIRRmIADGHEvANHTMTHPD-LSrCklaEVQsEIlgAcdvIraACPqAslRhv

YP_002499211.1 AAnePELIqRmIAEGHEvANHTMTHPD-LSrsesgEIQrEIlwANrvIrmtCPqAllRhm

AAL93156.1 AADqPkLIRRmIAEGHEvANHTMTHPD-LSrCeltQVQyEIlaAsrvIktACPqAalRhm

CAC42488.1 AADqPkLIRRmIAEGHEvANHsMTHPD-LSrCePtEVQqEIltAsrvIrmAsPrvslRYm

WP_013872874.1 RpPh-GilsagfLlAA--------rrleLTP-vlWtawgRDWtsTatpltvlDAltpdlR

WP_020462976.1 RpPh-GvlsvgVLaAA--------rrldLTP-vlWtawgRDWtaTatpanvlatlapdlR

YP_711985.1 RpPh-GilsaglLvAA--------rdleLTP-vlWSawgRDWtaeatprSvlDtlapdlR

WP_013872989.1 RpsgtstaTplILaeA--------gaAGyrtvvdfdVDPlDyts-PGADAvVarVrAgVR

WP_028230172.1 RAPa-GlrnpflepAl--------cslGLql-asWtrrgfDtra-qeAgAItrrlLdgla

WP_028211220.1 RAPa-GlrnpflepAl--------cslGLql-asWtrrgfDtra-qeAgAItrrlLdgla

WP_013871639.1 RpPY-ss-TpaaLTApayttyrelAsqGylt-vladIDsRDWtR-PGADAIVrAatpAgt

YP_003638438.1 RAPY-GrWgrtV-dvA--------AslGmrs-LeWqlaveDWdpaPpADvlVEr-LgAVe

YP_004404326.1 RAPY-GaWgqsp-TvA--------talGmrP-mgWqlavtDWvp-PGtDelarrltegVt

YP_004242916.1 RAPY-GsWgqtp-evA--------AelGmqP-LgWqltigDWep-PGtselVrrIregIR

YP_290844.1 RAPY-GsWgqsp-qvA--------AdmGmqP-LgWamDiaDWep-PGtselVrrlnerVt

YP003833384 RqPg-GrWTaeeVTiA--------qqmGLrP-LHWSVDPqDWdh-PpAktIIkrVkgAtR

YP_004402816.1 RqPg-GrWTaeVVkvA--------kqlemrs-LHWtVDPqDWaR-PtAatIqkrVqrAaR

YP_004082445.1 RqPg-GaWTpsVMSAc--------AdlsLTP-LHWSVDPsDWka-PGAttIeamVrSqmg

EWM67253.1 RqPg-GaWTrpVISAc--------AdlGLTP-LHWSVDPsDWra-PGAltIesrIrtqmg

YP_001157155.1 RqPg-GaWTysVVSAA--------qelGLTP-LHWtIDPRDWet-PGADpIattVLdeVg

YP0_04408115.1 RqPg-GaWTypVISvS--------rdlGmTP-LHWtlDPsDWra-PGAtrIasvVLSeaR

YP_003115329.1 RAPg-GnWsptVfSvc--------AklGLra-LdWSVDPRDWSR-PGtDhIVqtVMghth

YP_715094.1 RAPg-GdWspaVLaAA--------AglGmTs-LgWdVDPRDWaR-PGtaSIrqS-LggaR

YP_004014355.1 RAPg-GdWsppVfSAe--------AsrGLTP-vaWnVDPRDWSR-PGtqkIVtSlLA-aK

YP_001509470.1 RsPg-GdWppaVLaAA--------AaqhLTP-vgWSVDPRDWtR-PGAaSIVrS-LtgaR

YP_004332108.1 RAPg-GyWsptmLTdA--------AtrnLqP-LgWSVDPRDWkR-PGvDAIVaAVqkqVh

WP_012172340.1 RAPY-GaWstqILnvv--------nkiGLrP-vsWSVDPRDWea-PriEnlINeILdNaR

AAB51163.1 RAPY-GaWstqILnvv--------nkiGLrP-vsWpVDPRDWea-PriEnlINeILdNaR

ZP_07980118.1 RAPY-GeWdrtVyTlg--------ArhGmeP-LaWSVDteDWSR-PGAaAItrrVLAgaR

WP_013873451.1 RtPY-GrWndgarrvA--------AelGLqP-LgWtIDsRDWSa-PGvteImDAlrqqlh

AAK50871.1 RAPY-GrWTdsVIycA--------AqrGmlP-LYWSIDPRDWSR-PGvDkIVsvVLeAaR

CAD43932.1 RAPY-GvWTpeVcveg--------mnAGLTP-LHWSVDPqDWaR-PGvDlIVDtVLATVe

YP_001796209.1 RAPY-GiWTpeVcveg--------mnAGLTP-LHWSVDPqDWaR-PGvDlIVDtVLATVe

WP_013874294.1 RAPY-GaWTgear-AA--------AlfGLeP-LnWSVDPRDWSR-PGANvIVEtVLtcIR

WP_037627918.1 RAPY-GvWTgearaAA--------AalGLaP-LdWtVDPRDWSR-PGvDAIVEtVLthIR

YP_003610199.1 RAPY-GiWTkdaIget--------gkAGLia-LHWSVDPRDWSQ-PGvNAIVDvVMASVR

ACT34139.1 RAPY-GaWseeVfTAS--------eiAGLaa-LHWSVDPRDWSR-PGSDAIVDAVvASVR

ACT34142.1 RAPY-GaWseeVfTAS--------eiAGLaa-LHWSVDPRDWSR-PGtDAIVDAVLASVR

ACT34099.1 RAPY-GaWseeVfTAS--------eiAGLaa-LHWSVDPRDWSR-PGtDAIVDAVLASVR

ZP_03526066.1 RAPY-GiWTeeVLkvS--------AnAeLTa-vHWSVDPRDWSl-PGADgIVNdVLqSVR

AAD11394.1 RAPY-GaWnedVLStS--------mhAGLrP-vHWSVDPKDWSR-PGvDAIVDAVLAdaR

CAA68620.1 RAPY-GvWTedVLSAS--------vrAGLga-vHWSaDPRDWSc-PGvDvIVDeVLAAaR

AAY26501.1 RAPY-GiWTqeVLamS--------AsAGLTa-vHWSIDPRDWSR-PGvDrIVNSVLANVR

ABD67418.1 RAPY-GaWseeaLTAS--------AsAGLTa-vHWSaDPRDWSR-PGADAIVDAVLASVR

ABG91001.1 RAPY-GvWseeaLTrS--------AsAGLTa-iHWSaDPRDWSR-PGANAIVDAVLASVR

ABD67421.1 RAPY-GvWseeaLarS--------AsAGLTa-vHWSaDPRDWSR-PGANAIVDAVLASVR

AAL93157.1 RAPY-GrWTeeVLatA--------AmAGLgm-vHWSVDPRDWSR-PGvDAIVNAVLASIR

YP_002499211.1 RAPY-GsWTeeVLTtA--------ArAGLaa-LHWSVDPRDWSR-PGvDAIVDAVLSSVR

AAL93156.1 RAPY-GmWseeVLTtS--------ArAGLaa-LHWSVDPRDWSR-PGvNAIVDAVLASVR

CAC42488.1 RAPY-GmWTeaVLTtS--------AsAGLaP-LHWSVDPRDWSR-PGvDAIVsAVLASVR

WP_013872874.1 gGgtVLLHDsdctsa------pqawhsalgALpefaarchdR-Glr-----L--------

WP_020462976.1 gGAtVLLHDsdctsa------pGawrsalgALpeLaarcdda-slr-----L--------

YP_711985.1 gGAtVLLHDcdctsa------pGawrsalgALpeLaarcdda-Glr-----L--------

WP_013872989.1 PGsIVsmHfGyP--------------gTVtAfaRiVtnLrta-Glv-----p--------

WP_028230172.1 PrdIlLvHDGhaard------argeplvlevLppLlrAaadn-dlq-----w--------

WP_028211220.1 PrdIlLvHDGhaard------argeplvlevLppLlrAaadn-dlq-----w--------

WP_013871639.1 dGAvVLfHDs-----------GGdRsQTVaAvdQLldvLdAR-GYr-----L--------

YP_003638438.1 PGgvVLLHDG-----------GGdRsaTVeAvrRvVPALrAa-Gwt--------------

YP_004404326.1 PGAvVLLHDG-----------GGdRsQTVdAvdlvIPpLlAd-Gwr--------------

YP_004242916.1 PGgvVLLHDG-----------GGdRtQTVeAvdQvIPvLkAe-Gwk-----f--------

YP_290844.1 PGAviLLHDG-----------GGdRsQTVqAvdQvIPqwkAq-Gwt-----ftfpagapt

YP003833384 hGsvVLmHDa-----------GGdRaQTmaAcrhLIPdLkrRyGia--------------

YP_004402816.1 PGsvVLLHDG-----------GGnRaaTlaAcpKvIaALk-R-dhg-----I--------

YP_004082445.1 PGAIVLmHDa-----------GGdRsgTVsALqQLlPeLlAR--Fe-----L--------

EWM67253.1 PGAIVLmHDa-----------GGdRsgTVaALqQLlPeilAR--Fe-----L--------

YP_001157155.1 PGsIVLLHDG-----------GGpRqdTVdALsQilPeLitR--Fp-----V--------

YP0_04408115.1 PGsIVLLHDa-----------GGdRqgTVdALyRilPdLtAR--Fd-----L--------

YP_003115329.1 tGsIiLeHDG-----------GGdRsQTVaALqRflPqLlea-GYk-----f--------

YP_715094.1 PGdIlLcHDG-----------GGnRaQTVaALqRvlPlLrAR-Gls-----f--------

YP_004014355.1 PGdIlLcHDGdgehpa-----GvdRsETVqALrtvlPqLkSR-Glt-----f--------

YP_001509470.1 aGdIlLcHDG-----------GGdRsQTVeALrQvlPALrAR-Glt-----f--------

YP_004332108.1 PGAIVLLHDG-----------GGnRsQTVaALeRLlPwLsAq-GYt--------------

WP_012172340.1 PGsIiLLHDGCPPDEaamwdvrGgRaQTlaALryvVPALqAR-GFa-----L--------

AAB51163.1 PssIiLLHDGCPPDEaamwdvrGgRaQTlaALryvVPALqAR-GFa-----L--------

ZP_07980118.1 PGAvVLcHDG-----------GGdRaQTVtALrRflPrLrAd-Gwd-----L--------

WP_013873451.1 PGgIVLLHDGClPErkrgcs-aGhqDQTlavvplLIreLrhq-GlvpgpplL--------

AAK50871.1 PGsviLLHDGCPPnEqGggsrncrRDETVlAvaRLIPALhgl-Glk-----I--------

CAD43932.1 PGAIVLLHDGsPPnElasrpytasREQTVrALpRLIsALkeR-nFv-----I--------

YP_001796209.1 PGAIVLLHDGsPPnElasrpytasREQTVrALpRLIsALkeR-nFv-----I--------

WP_013874294.1 PGgviLLrgGCPaDEwpsgsrtGlRErTViALrRLIPALhey-GFv-----f--------

WP_037627918.1 PGgviLLHDGCPPDElprgdrthlREQTVaALrRLIPALrdR-GFv-----f--------

YP_003610199.1 PGsIVLLHDGsPPgEsnsdvhttsRrQTaeALsRLIPALkAR-rFv-----I--------

ACT34139.1 PGAIVLLHDGCPPDElrpsTqaslRDQTakALseLIPALnAR-GYe-----I--------

ACT34142.1 PGAIVLLHDGCPPDElrpsTqaslRDQTakALsQLIPeLnAR-GYe-----I--------

ACT34099.1 PGAIVLLHDGCPPDElrpsTqarlRDQTakALsQLIPALnAc-GYe-----I--------

ZP_03526066.1 PGsIVLLHDGCPssE---------------------------------------------

AAD11394.1 PGAIVLLHDGCPPDEiGtcTltGlRDQTlsALpaiIPALhAR-GFs-----f--------

CAA68620.1 PGAIVLLHDGCPPDEveqcslaGlRDQTliALsRiIPALhSR-GFe-----I--------

AAY26501.1 PGAIVLLHDGyPPDEeGlcTdaslRDQTtmALayLIPALqrR-GFv-----I--------

ABD67418.1 PGAIVLLHDGCPPDEsGal--tslRDQTlmALsRiIPALheR-GFa-----I--------

ABG91001.1 PGAIVLLHDGCPPDEsGal--tGlRDQTlvAisRmIPALheR-GFa-----I--------

ABD67421.1 PGAIVLLHDGCPPDEsGal--tslRDQTlmAisRiIPALheR-GFa-----I--------

AAL93157.1 PGAIVLLHDGCPPsEqrqcThaGlRDQTltALshLIPALqeR-GFa-----I--------

YP_002499211.1 PGAIVLLHDGCPPsElapcThaGlRDQTVvALaRLIPeLhSR-GFt-----I--------

AAL93156.1 PGAIVLLHDGCPPEElGraTharvRDQTVlALshLIPALhrR-GFs-----I--------

CAC42488.1 PGAIVLLHDGCPPDElGrcThaGrREQTlmALslmIPALhdR-GFa-----I--------

WP_013872874.1 ----gPLaehglrpgplaehglrpPGpLathgprAseapAg-------------------

WP_020462976.1 ----gPLaehglrpvsr-------------------------------------------

YP_711985.1 ----gPLaehglrgr---------------------------------------------

WP_013872989.1 ----vsvrdllv------------------------------------------------

WP_028230172.1 ----atLraclddqtghp------------------------------------------

WP_028211220.1 ----atLraclddqtghp------------------------------------------

WP_013871639.1 ----vtLsdglrlpsdvtThpadtatrLrgqallAtyriSstLtwtLTaflvplTvLtll

YP_003638438.1 ----ftvPt---------------------------------------------------

YP_004404326.1 ----faLPaptgastgr-------------------------------------------

YP_004242916.1 ----drparrg-------------------------------------------------

YP_290844.1 npgptPtPtptptptptpTpgpgnPGatcdvdytvvndwghgMqgaITvsntgsSpInnw

YP003833384 ------LPr---------------------------------------------------

YP_004402816.1 ----trLr----------------------------------------------------

YP_004082445.1 ----eaLPvgsp------------------------------------------------

EWM67253.1 ----esLPvgpq------------------------------------------------

YP_001157155.1 ----aaLPpsit------------------------------------------------

YP0_04408115.1 ----qaLPkhgt------------------------------------------------

YP_003115329.1 ----vqp-----------------------------------------------------

YP_715094.1 ----vPL-----------------------------------------------------

YP_004014355.1 ----vtL-----------------------------------------------------

YP_001509470.1 ----mPL-----------------------------------------------------

YP_004332108.1 ----ftfP----------------------------------------------------

WP_012172340.1 ----qPLP----------------------------------------------------

AAB51163.1 ----qPLP----------------------------------------------------

ZP_07980118.1 ----vrpdrrltrgaar-------------------------------------------

WP_013873451.1 ----eaLPrlpaagtpmgygw---------------------------------------

AAK50871.1 ----aaLPnle-------------------------------------------------

CAD43932.1 ----ssLPklrgqinpvrTa----------------------------------------

YP_001796209.1 ----ssLPklrgqinpvrTa----------------------------------------

WP_013874294.1 ----qPLPapsvhspisrpv----------------------------------------

WP_037627918.1 ----rtLP----------------------------------------------------

YP_003610199.1 ----srLPdrrsqtlae-------------------------------------------

ACT34139.1 ----raLPqhh-------------------------------------------------

ACT34142.1 ----raLPqhh-------------------------------------------------

ACT34099.1 ----raLPqhh-------------------------------------------------

ZP_03526066.1 ------------------------------------------------------------

AAD11394.1 ----rsLPr---------------------------------------------------

CAA68620.1 ----rsLP----------------------------------------------------

AAY26501.1 ----rPLPqlh-------------------------------------------------

ABD67418.1 ----rPLPphh-------------------------------------------------

ABG91001.1 ----rPLPphh-------------------------------------------------

ABD67421.1 ----rPLPphh-------------------------------------------------

AAL93157.1 ----ssLPqlh-------------------------------------------------

YP_002499211.1 ----rsLPqhh-------------------------------------------------

AAL93156.1 ----rsLPqph-------------------------------------------------

CAC42488.1 ----rsLPqpdrtnqtpyelawrn------------------------------------

NodC alignment

YP_00141170.1 ------------------------------------------------------------

YP_00141184.1 ------------------------------------------------------------

ZP_07305288.1 ------------------------------------------------------------

YP_00401411.1 ------------------------------------------------------------

YP_00422371.1 ------------------------------------------------------------

YP_00458210.1 ------------------------------------------------------------

ZP_06415311.1 ------------------------------------------------------------

YP_00151150.1 ------------------------------------------------------------

YP_00331559.1 ------------------------------------------------------------

YP_00348965.1 ------------------------------------------------------------

ZP_07307227.1 ------------------------------------------------------------

ZP_08293056.1 ---------MLVCLLILLACgaVKtLFKRGmRfRsVrvrak---------grgrvttvrt

ZP_08232398.1 mtwsfgplvLLVCLVILVACfgVKlLFKRGtRlRvVrAvhReGygegwgagrggvvavrs

BAJ27055.1 -----------------------------------------------------------m

YP_003491630.1 ---------------------------------------------------------mtr

YP_00339317.1 ------------------------------------------------------------

YP_884889.1 ------------------------------------------------------------

YP_00107328.1 -------------------------------------------mstvdtlappSvreprs

ZP_06920913.1 ------------------------------------------------mpgqrgappvGr

ADW02611.1 -----------------------------------mnAlhRtpvrPplpvphgArtprGl

ZP_05523903.1 ------------------------------------mtvlRpGhgPaapirsaArhrlG-

ZP_08389379.1 ------------------------------------------------------------

YP_00126324.1 ------------------------------------------------------------

WP_01217233. ------------------------------------------------------------

AAB51164.1 ------------------------------------------------------------

EMF57041.1 ------------------------------------------------------------

YP_004582102.1 ------------------------------------------------------------

AEH05539.1 ------------------------------------------------------------

YP_003610198.1 ------------------------------------------------------------

YP001863657.1 ------------------------------------------------------------

CCA66310.1 ------------------------------------------------------------

ACB86875.1 ------------------------------------------------------------

YP_00131473.1 ------------------------------------------------------------

ZP_07595040.1 ------------------------------------------------------------

NP768667.1 ------------------------------------------------------------

YP_001984572.1 ------------------------------------------------------------

ACT34131.1 ------------------------------------------------------------

ACA23959.1 ------------------------------------------------------------

ACT34140.1 ------------------------------------------------------------

ACT34100.1 ------------------------------------------------------------

YP_00141170.1 ------------------------------------------------------------

YP_00141184.1 ------------------------------------------------------------

ZP_07305288.1 --------------msaararasrvrftVvtgvvvaalAaatvvVhlqSkspIltfywWl

YP_00401411.1 ----------------------------------mpgdigyylvyLpLgtiglvrwlcWl

YP_00422371.1 --------------------------------madasfgeqlmwVLpfgYlgIvswgfWl

YP_00458210.1 ----------------------------MdaLsTvidrvgsyWgLvpLgiagViswsvWl

ZP_06415311.1 ----------------------------MewLgdAvqfirdhrsLvpLgiagVvswvvWl

YP_00151150.1 ----------------------------MewfgvAfdfvrdhrsLvpLgiagVvswvvWl

YP_00331559.1 --------------mpsaipLaypasafsvgqldvsdlAdqavhLfpLAvagIivwslWi

YP_00348965.1 ----------mdkapaggsgfVsdffddVwswivmataetswreLMpLgiagafvwglWl

ZP_07307227.1 --------------------------------------------------mgllsl----

ZP_08293056.1 avTTtdeAqRsadatglpraLMrdgglmpavLvvlgvvgwlcWrVLvsrHhvldtwvvWs

ZP_08232398.1 pvTTpdeAqRsadatglpraLMraggplpavVvalgvvgwlcWrVLvsrHhvldawivWt

BAJ27055.1 arvTpasAlRrrtLlllgtaaLalaylaahhVlaAgrlpdgdappLatvYalafgwfllr

YP_003491630.1 phTltahphRsttVltgaisLtlaagwaahhgiTAadygadtssrLaavWsvtflllltq

YP_00339317.1 -------------ManwadhaMrvvaaaylaLllAlavAyhglwfdeVAgdalfaiyglv

YP_884889.1 ------mAtiaaadrtvtsvrpaelgvkVviVmalllivmliFaykvVSlhnmkdepfWa

YP_00107328.1 lafagwldsRpaeVrrglrrVLvliglmpliVllAvqapllsHgaLlLgYgmlvltatvs

ZP_06920913.1 arlTaltdrmdpgVrhaarrLLvvimllpllLilAheaprlvqapLvLgYgflvltvtvs

ADW02611.1 raverrvAradpaIrhgvrrLLvllllmpllLvlAhhtArlprlsLpVcYglVvlagtit

ZP_05523903.1 ----trldaldprVrrdvvrLLtllallpllLllArgavrlpHafdpLAlyglavlagtv

ZP_08389379.1 --------------------------------------------------avVmaglaWf

YP_00126324.1 ------------------------------------------------------------

WP_01217233. ----------------------------MsvVdvigllAtaaYvtLasAYkvVqfinvss

AAB51164.1 ----------------------------MsvVdvigllAtaaYvtLasAYkvVqfinvss

EMF57041.1 -----------------------mmaavtsiLsTAaltvsvaYaVLvVvYqaVqaryvWr

YP_004582102.1 ------------------------mstaVsiLsTAnmivpssYatLaIgYqgaktfyarr

AEH05539.1 ------------------------------------------------------------

YP_003610198.1 ------------------------------------------YaLLsavYktaqvlhtlp

YP001863657.1 ----------------------------MnipaTinttSvllYaLLstiYksaqvlhamp

CCA66310.1 ------------------------------------------------------------

ACB86875.1 ------------------------------------------------------------

YP_00131473.1 ------------------------------lLdTtstaAisiYaLLltAYrsmqalharp

ZP_07595040.1 ------------------------------lLdTtstaAisiYaLLltAYrsmqvlharp

NP768667.1 ----------------------------MdlLaTtsaaAvssYaLLstiYksVqalyaqp

YP_001984572.1 ----------------------------MdmLdTtstvAvslYaLLstAYksVqavyslp

ACT34131.1 ----------------------------MdlLaTtstvAvscYaLLsaAYksmqvayalp

ACA23959.1 -------------------------------------------------Fag--------

ACT34140.1 ----------------------------MdlLaTAstvAvscYaLLsaAYksmqmayalp

ACT34100.1 ----------------------------MdlLaTtstaAvscYaLLsaAYksmqmayalp

YP_00141170.1 ------------------------------------------------------------

YP_00141184.1 -----------------trhgSrhfKPfde--navsP--TamVsIhVPihNEpPEmvret

ZP_07305288.1 g---------------msvivlclvsflkgrsfthlPvapgrtvaIIPAFeEpsEnLhRt

YP_00401411.1 a----------------rripAalyRPvgg--dlrlP-----ltVVVPvyqEDPEvftRa

YP_00422371.1 v----------------rkilAataRPvk---nlyrt--T--ttVVVPSFhEDPDvLlRC

YP_00458210.1 f----------------rrvlSsryKPin---nkfra--S--tsVVVPSFrEDPDvieRC

ZP_06415311.1 t----------------rrilStryRPir---nnfrt--T--tsVVVPSFrEDPDvLmRC

YP_00151150.1 t----------------rrllStryRPvr---nnfra--S--tsVIVPSFrEDPDvLiRC

YP_00331559.1 y----------------rvvlSrraRPvv---sdhrt--T--tsVVVPSFhEDPDiLlRC

YP_00348965.1 y----------------ravlSrfaKPvv---nnyrt--T--VsVVVPAyrEDasiLldC

ZP_07307227.1 -----------------kmfgAlfyRPakagreeleylENawVtaVIPiyNEDPvmfeQg

ZP_08293056.1 f-------------dvlfvivAvqlvvagfEgrvvgrgETyrVaVlVPlyNEDPDvvvRm

ZP_08232398.1 f-------------dvlfvivAvqlvvagfEgrvigrgEAhrVaVlVPlyNEDPDvvvRm

BAJ27055.1 t---------------vlaylerpyRatpaq-qaald--rahlaalVPvyNEDPgwLrRC

YP_003491630.1 t------------------vmyhceRPrqatprarrqlDAlhVaVlmPvyNEDPgyLryg

YP_00339317.1 v----------vSyivsrfvlSipyRPprd--vgheP--h--VaIVmPAFNEe-avvaRs

YP_884889.1 aygliitaFIf-----trfglAalyRPprrDtgnywP--v--VaIIVPAyNEp--diaRt

YP_00107328.1 m-----mylgfaryedpsvhpStdagardrcdfpalP--plprvsllvAvrdevDgieeC

ZP_06920913.1 m------LFIAYSRyddpsrrTlrgRPrhlDrfpelP--ArarvsflvAvkdeeDgieaC

ADW02611.1 l------LYIAYSRyedpaegelrvRPgpeEtfpplP--AhpmvafllAvkdevDgieaC

ZP_05523903.1 c-----lLhLAYSRyddpavrplrsRPrhaEafpalP--ArprvsfllAvrnerahieaC

ZP_08389379.1 q--------------------SrrsKPvp---peytP--S--VsVIIPAFNEe-rviaQs

YP_00126324.1 ---------------------------------pvdP--drfVtVlIPAyNEe-rvieRs

WP_01217233. v---------------------tdvaglesDalpltP----rVDVIVPtFNEnsstLleC

AAB51164.1 v----------------tdvaglesda-----lpltP--r--VDVIVPtFNEnsstLleC

EMF57041.1 a---------------------drhsPtpvDpmgalP--S--VDVIIPcyNEDPllLaeC

YP_004582102.1 a-----------------yrpTtthhadp---cdslP--S--VDVIIPcyhEDPltLaaC

AEH05539.1 ------------------------------------------------------------

YP_003610198.1 a-------------------pArhsnqsa---tnflP--d--VDVIVPcFNEnPDtLraC

YP001863657.1 s-----------------sgpparsKstn---snslP--d--VDVIVPcFNEnPDtLraC

CCA66310.1 ------------------------------------------------------------

ACB86875.1 ------------------------------------------------------------

YP_00131473.1 i-----------------dgpAvsaePve---trplP--A--VDVIVPSFNEDPgiLsaC

ZP_07595040.1 i-----------------dgpAvsaePve---trplP--A--VDVIVPSFNEDPgiLsaC

NP768667.1 a-----------------insSldnlgqa---evvvP--A--VDVIVPcFNEnPNtLaeC

YP_001984572.1 t-----------------dvslasqslag---feelP--S--VDVIVPSFNEDPrtLseC

ACT34131.1 m-----------------drspasdnlvd---fdllP--S--VDVIVPcFNEDPsaLsaC

ACA23959.1 ------------------------------------------------------------

ACT34140.1 m-----------------drspmsdelvg---fdplP--S--VDVIVPSFNEDPsaLsaC

ACT34100.1 m-----------------drspvsdelvg---fdplP--S--VDVIVPSFNEDPsaLsaC

YP_00141170.1 -------------fEfIVVptsfpqt-----------------kPKacnfaLpfarG---

YP_00141184.1 LqaLanlDYd--nyEVLVlDnntvDpE----vwqpVRdycAqlgPRFRFfhLenwpGfKa

ZP_07305288.1 vrSLLAQthP--vdEihViDDGSrqh-----pvepf-------DhprvFwhwqeNkG-KR

YP_00401411.1 LtSwLAnD----vdEVIlViDatdta---------cRriaA--DfpvtvLvte-vpG-KR

YP_00422371.1 LqtwvrQ----GPsriVIVlDvadve-----aqsrIeal----glpsvdVvMfhhrG-KR

YP_00458210.1 LqtwLAQ----nPdEiIIVpDvedte-----lisrlRrhare-hrnlRvLpfv-heG-KR

ZP_06415311.1 LetwLAQ----ePdEiIIipDiedae-----liarlamra---DPtvRvIpfv-heG-KR

YP_00151150.1 LetwLSQ----qPdEiIIipDvedte-----liarlaqra---DPtvRvIpfv-heG-KR

YP_00331559.1 LdSwLSQ----dPtEVIVVlDvadle-----aysrIvarg---DeRvRpIlfh-haG-KR

YP_00348965.1 LdtwLeQ----dPtEVIIVpDvddte-----vlhrlsqvr---DPRvRvLafe-hrG-KR

ZP_07307227.1 mrSLLAQsrl--PnEihIiDDaSaNdsgik-aakklRrefeargvKYtvsvqpeNkG-KR

ZP_08293056.1 LSaLLhQssP--PaEihVVDDGStqgaYleerdwfIRraam--ariYatwqrtaNeG-KR

ZP_08232398.1 LSaLLhQssP--PaEihVVDDGStqgaYvaerdwfIRqaal--agiYaswqrtpNeG-KR

BAJ27055.1 LdSLLrQtrP--PdsihVVDDGStvdyta--erdrfRaacAaagiRat-wqrtpNrG-KR

YP_003491630.1 LeSLLAQtrc--PnsVhVVDDGSttgDYrllrawwIeaatA--agiattwvrqpNsG-KR

YP_00339317.1 LrSLLAlDYPvdklElVaiDDGStDe-----tlarmRevaAe-sPRvRvIeLgrNqG-KR

YP_884889.1 LlhcvgvDYPrellrVVVVDDkSsDd-----tlrrItdfaAe-hPnltvIphevNgG-KR

YP_00107328.1 vrtMvgsaYP--dlEVIViDDaStDg-----tpdvlRrlaA--EldvtvIfkeiNqG-Kk

ZP_06920913.1 vrSMagsDYP--nlQiVVVDDaStDg-----tpavlRrlar--ElplRvLyLehNvG-Kk

ADW02611.1 vrSMaAsDcP--gvriVVVDDhSqDg-----trdvlRrled--DlgitvVyLdaNvG-Kk

ZP_05523903.1 vrSMaAvDYP--dvQlVVVDDaSdDg-----tpdvlerlaA--ElpltlIrLeeNlG-Kk

ZP_08389379.1 vSrVLAsDYP--glQVIVVDDGSkDa-----tstvVRetfAg-EdRvRlLtLp-NgG-Ka

YP_00126324.1 vrrVLdsrdv--rvEVIViDDGSkDR-----tsavVaeafAd-EPRvRlLtVe-NgG-Ka

WP_01217233. vASIcAQDYr-GPitiVVVDDGStNKt----sfhaVcdkyAs-DeRFiFVeLdqNkG-KR

AAB51164.1 vASIcAQDYr-GPitiVVVDDGStNKt----sfhaVcdkyAs-DeRFiFVeLdqNkG--t

EMF57041.1 LrSVLAQDYe-GqlrVylVDDGSdNRs----rlepVhaahAd-DPRFtFVpLphNvG-KR

YP_004582102.1 LrSVaAQDYq-GelrVylVDDGStNRD----rlepVydtyAg-DaRFtFLlLphNvG-KR

AEH05539.1 -------------------------------------------------------vG-KR

YP_003610198.1 LASIatQEYs-GalkihVVDDGSaNRD----algpVyrdyeh-DPRFnFVlLpqNvG-KR

YP001863657.1 LASIatQEYa-GPfkVhVVDDGSaNRD----alrmVyheyeh-DPRFnFVlLprNvG-KR

CCA66310.1 ------------------------------------------------------------

ACB86875.1 ------------------------------------------------------------

YP_00131473.1 LASIadQDYP-GelrVyVVDDGSrNRE----aivraRafysr-DPRFsFIlLpeNvG-KR

ZP_07595040.1 LASIadQDYP-GelrVyVVDDGSrNRE----ailrVRafysr-DPRFsFIlLpeNvG-KR

NP768667.1 LeSIaSQDYa-GkmQVyVVDDGSaNRD----vvapVhriyAs-DPRFsFIlLanNvG-KR

YP_001984572.1 LASIagQEYg-GrlQVylVDDGSeNRE----alrpVheafAr-DPRFniLlLpqNvG-KR

ACT34131.1 LASIaSQqYa-GklrVyVVDDGSaNRD----vvvpVhgafAg-DsRFnFIlLdkNvG-KR

ACA23959.1 -------------------------------------------DPRFnFIlLdkNvG-KR

ACT34140.1 LASIaSQqYa-GklrVyVVDDGSaNRD----vvvpVhgafAg-DPRFsFIlLekNvG-KR

ACT34100.1 LASIaSQqYa-GklrVyVVDDGSaNRD----vvvpVhgafAg-DPRFnFIlLdkNvG-KR

YP_00141170.1 -----------------eflviyDaedapePqqlkKaVsafrlgDeklacVqaQLnyyNw

YP_00141184.1 gAlnfgLektAEea---eiIavIDSDyqVePswlkvLVpyf--dkqdVGfVqGpqdyrdR

ZP_07305288.1 gAQvtvLRhlqEqnkqfDyILtIDSDgepfPDaleqqLraM--snPrIqAttGmIyirNf

YP_00401411.1 dAlrrgwe-aAst----eLValVDSDTiwAdDVaAevckpf--aDPaVGgVatrqnvyNp

YP_00422371.1 sAlgvgIR-mvDt----eLILfVDSDTawedgMldavqmpf--iDPaVGAVstrqnvylp

YP_00458210.1 sAlgvgIK-aAtk----eiVvlcDSDTawePgLlAavqmpf--vDPkVGgVgtrqsvyNa

ZP_06415311.1 sAlgvgLs-aAtm----DvVvlcDSDTawePgLlAavqmpf--iDPqVGgVgtrqnvyep

YP_00151150.1 sAlgvgLs-aAtr----DiVvlcDSDTawePgLlAavqmpf--vDPqVGgVgtrqnvyep

YP_00331559.1 sAlgagIR-aSry----evlvltDSDTSwePgLlesvqmpf--vDPrVGgVgtQqnvyqR

YP_00348965.1 sAlgvgIR-aAts----eLVvlVDSDTrwePgLlAavqmpf--vDPeVGgVgtQqnvyqR

ZP_07307227.1 EAlalgfeaapys----DifLcVDSDTvLSrDtVreLLlpl--aDekImAstGmVlAlNh

ZP_08293056.1 hAQihgfRkirDa----DLfvtVDSDsmLdaealheIVqpf--sDPrVmsVaGvIlAiNn

ZP_08232398.1 hAQvhgfRkirDa----DLfvtVDSDsmLdaealheIVqpf--sDPrVmsVaGvIlAiNn

BAJ27055.1 aAQsaAseqapEa----DyfLtlDSDadLdPaalheLLqpf--aDartqsVaGvVlAaNa

YP_003491630.1 hAQaagVRacpqa----DvfvtVDSDscLdhraVeeILlpf--arrdVqsaaGiVlAtNh

YP_00339317.1 aAmaagmR-aTDa----eilafVDSDsSLdPDalhKLVqgf--aePgVGAVcGhadvaNv

YP_884889.1 RAmatgmaaadDa----DLfvfIDSDsqVtrDaIrvIasyf--aDssVGAVcGhtdvtNi

YP_00107328.1 hAltdgVR-vAsG----evlaftDSDcvLAPDalSRcVral-VenPrlGAVsGharAlNa

ZP_06920913.1 hAlvrAae-lADG----DilaftDSDcvLAPDalrRcVtal-alhrelGAVsGharAlNa

ADW02611.1 hAlvrAcefaADt----eiIaftDSDcvLAPDalrRcVral-VthPelGAVsGhcrAlNt

ZP_05523903.1 gAlvrAca-vADG----DvlLftDSDcvVAPDaVrhcVtal-VrhPelGAVgGhcrAlNt

ZP_08389379.1 aAlnrALR-eAtG----evVIalDaDTqfePetIAKLarwf--aDPklGAVaGdarvgNR

YP_00126324.1 RAlnrgLe-lvqG----eiVIalDaDTqfePatIARLarwf--dDPalGAVaGnakvgNR

WP_01217233. aAQmeAIR-rTDG----DLILnVDSDTvIdkDVVtKLassM--raPnVGgVMGQLvAkNR

AAB51164.1 aAQmeAIR-rTDG----DLILnVDSDTvIdkDVVtKLassM--raPnVGgVMGQLvAkNR

EMF57041.1 KAQveAIR-rSgG----eLVvnIDSDTTIePgVVrKLaakM--aDPaVGgaMGQMvArNR

YP_004582102.1 KAQvaAIR-rSaG----DLVvnVDSDTTIePDVVrKLaakM--tDPaVGAaMGQMvArNR

AEH05539.1 KAQiaAIR-sSyG----efILnVDSDTTLAqDVVknLVlkM--gDPsIGAaMGQLtAsNR

YP_003610198.1 KAQiaAIR-aSaa----eLILnVDSDTTLAPDVISqLIltM--sDaeVGAaMGQLtAsNR

YP001863657.1 KAQiaAIR-sSsa----eLILnVDSDTTLAPDVVkKLVltM--sDPaIGAaMGQLtAsNR

CCA66310.1 ------------------------SDTTLAPDaVkKLVltM--sDatVGAaMGQLiAsNR

ACB86875.1 -------------------------DTTLAPDaVkKLVltM--sDatVGAaMGQLiAsNR

YP_00131473.1 KAQiaAIg-qSsG----DLVLnVDSDsTIAfDVVSKLaskM--gDPeVGAVMGQLtAsNs

ZP_07595040.1 KAQiaAIg-qSsG----DLVLnVDSDsTIAfDVVSKLaskM--rDPeVGAVMGQLtAsNs

NP768667.1 KAQiaAIR-sSsG----DLVLnVDSDTiLAaDVVtKLVlkM--hDPgIGAaMGQLiAsNR

YP_001984572.1 KAQiaAIR-rSaG----DmVLnVDSDTiLAsDVIrKLVpkM--qDPaVGAaMGQLtArNR

ACT34131.1 KAQivAIR-rSsG----DLVLnVDSDTTLAsDVVAKLarkM--qDPaVGAaMGQLvAsNR

ACA23959.1 KAQivAIR-rSsG----DLVLnVDSDTTLAsDVVARLalkM--qDPgVGAaMGQLaAsNR

ACT34140.1 KAQivAIR-rSsG----DLVLnVDSDTTLAsDVVAKLalkM--qDPaVGAaMGQLaAsNR

ACT34100.1 KAQivAIR-rSsG----DLVLnVDSDTTLAsDVVAKLalkM--qDPaVGAaMGQLvAsNR

YP_00141170.1 rEnWLTRqfaLeYaaffDlmlptmARlrlpi-plgGtsthfRtelLrnagawdpnn----

YP_00141184.1 hEsafknMayweYagffHigmvqrnnFnAi--iqhGtmtqvRkSALkrVggwaEwc----

ZP_07305288.1 eETWvsRaaDidigtSCvmmRssrsmlGAle-ttsGalAlYRaelLydhLDaYEve----

YP_00401411.1 r-gfLqRItDLfldcryfdEnAAQsvmGraLsClsGrtAiYRRalLveVsheFmTekFLG

YP_00422371.1 kssvwrRvaDwiidlrytdyapAmgRFGgii-CasGrtAAYRtSviqprvEdlEheiFfG

YP_00458210.1 rtsvwrRvanwlvdiryldyvpAQgRaGAVa-ClsGrtAAYRRSAilpVLhnlEnefFLG

ZP_06415311.1 rssvwrRvanwlvdiryldyvpAQgRvGAVa-ClsGrtAAYRRSAilpVLhnlEhefFLG

YP_00151150.1 rssvwrRvanwlvdiryldyvpAQgRvGAVa-ClsGrtAAYRRSAilpVLhnlEhefFLG

YP_00331559.1 tssvwrRIaDwlvnlryydyvpAmgsaGAVp-ClsGrtAAYRRdAvvpVLDdlEnefFLG

YP_00348965.1 rtsvwrRIaDwlvnlryydyvpAmgRaGAVa-ClsGrtAAYRRaAiepVLEnlEnefFLG

ZP_07307227.1 dsnifTRLqDLrYgnSflfERAAysRlksVL-CCCGalsAYRgSlvrkyLpdFlnQqFLG

ZP_08293056.1 rEnlLaRvtDvifvgqqliDRsfmsQlGsVM-vnsGglAAYRcSiLaenIDtYmnesyLG

ZP_08232398.1 rEnlLaRvtDvifvgqqliDRsfmsQlGsVM-vnsGglAAYRcSiLaenIDtYlSesyLG

BAJ27055.1 rtntLTRctDLylttcqlnERscQsvlGsVL-vnsGalAAYRapvLrehrElYlTetFLG

YP_003491630.1 hrnlLTRItDLwflvgqltDRsAlsamGAVL-vnsGPlAAYRaavvrdnLDaYlSetFMG

YP_00339317.1 dDsWLTRMqvvrYFvAfqvvKAsEsiFacVt-CCsGcfAAYRRdAilphLDwwEhQtFLr

YP_884889.1 shniLTRMqaMqYYiAfriyKsAEAlFGsVt-CCsGcfsAYRadAvrpVaDkwlnQtFLG

YP_00107328.1 aDTvLTRaqDtwYdgqfrvaKAAEAtFGnVt-CvsGPlAvfRRdAivnyLpawandtFLG

ZP_06920913.1 dEsvLskaqDvwYegqfrvaKAAEAtFGsVs-CvsGPlAvfRRdAivnyLpawagdrFLG

ADW02611.1 dsgfLaRaqDvwYegqfrvsKAAEAsFGsVs-CvsGPlAvfRRdAifnyLpawaddrFMG

ZP_05523903.1 daglLaRvqDiwYegqfrisKAAEAaFGsVt-CvsGPlAAfRReAvwnyLpawaedrFLG

ZP_08389379.1 v-nlvTRwqaveYitAqNlERrAlAgFdAmt-vvpGavgAwRRaALdaVggypEdt----

YP_00126324.1 i-nlvTRwqaLeYitAqNlERrAlARlnAmt-vvpGavgAwRlaAiraVggyppdt----

WP_01217233. ersWLTRLiDMeYWlACNeERiAQsRFGsVM-CCCGPCAmYRRSAitpLLaeYEhQtFLG

AAB51164.1 ersWLTRLiDMeYWlACNeERiAQsRFGsVM-CCCGPCAmYRRSAitpLLaeYEhQtFLG

EMF57041.1 haTWLTRLvDMeYWmACNgERAAQseFGAVM-CCCGPCAvYRRSALlkVLDqYETQyFrG

YP_004582102.1 raTWLTRLiDMeYWiACNeERAAQAeFGAVM-CCCGPCtvYRRSvLlqVLDqYETQlFrG

AEH05539.1 kqsWLTRLiDMeYWlACNeERAAQgRFGAVM-CCCGPCAiYRRSALhpLLDkYETQfFrG

YP_003610198.1 sDTWLsRLiDMeYWlACNeERAAQARFGAVM-CCCGPCAiYRRSALllLLDqYETQmFrG

YP001863657.1 rDTWLTRLiDMeYWlACNeERAAQARFGAVM-CCCGPCAiYRRSALllLLnqYETQtFrG

CCA66310.1 sDTWLTRLiDMeYWlACNeERAAQARFGAVM-CCCGPCAiYRRtALllLLDqYETQmFrG

ACB86875.1 sDTWLTRLiDMeYWlACNeERAAQARFGAVM-CCCGPCAiYRRtALllLLDqYETQmFrG

YP_00131473.1 gDTWLTkLiDMeYWlACNeERAAQARFGAVM-CCCGPCAmYRRSALasLLDqYETQlFrG

ZP_07595040.1 gDTWLTkLiDMeYWlACNeERAAQsRFGAVM-CCCGPCAmYRRSALasLLDqYETQlFrG

NP768667.1 nqTWLTRLiDMeYWlACNeERAAQARFGAVM-CCCGPCAmYRRSALalLLDqYEaQfFrG

YP_001984572.1 nDsWLTRLiDMeYWlACNeERAAQARFGAVM-CCCGPCAiYRRSALasLLDqYESQyFrG

ACT34131.1 sDTWLTRLiDMeYWlACNeERAAQARFGAVM-CCCGPCAmYRRSALllLLDqYETQlFrG

ACA23959.1 sDTWLTRLiDMeYWlACNeERAAQARFGAVM-CCCGPCAmYRRSALllLLDqYETQlFrG

ACT34140.1 sDTWLTRLiDMeYWlACNeERAAQARFGAVM-CCCGPCAmYRRSALvlLLDqYETQlFrG

ACT34100.1 gDTWLTRLiDMeYWlACNeERAAQARFGAVM-CCCGPCAmYRRSALllLLDqYETQlFrG

YP_00141170.1 ----vtEDadLglrfalhGYR-------------------------csiIrStte-eean

YP_00141184.1 ----icEDaeLgikLyrAGYd-------------------------svYVnhsfgrgltP

ZP_07305288.1 --cgtGdDRwLalraLmrGe--------------------------vvaVneAgvvTdmP

YP_00401411.1 vqcmsGdDKrLTtLLLerGHa-------------------------TvlqrSArvwssfP

YP_00422371.1 KeciaGdDgrmTwLvLsqGFR-------------------------vahqdSArAlsmfP

YP_00458210.1 rrciaGdDgrLTwLtLaSGYk-------------------------TvhqdTArAmsmfP

ZP_06415311.1 rrciaGdDgrLTwLvLaSGYk-------------------------TmhqhTAhAmsmfP

YP_00151150.1 rrciaGdDgrLTwLvLaSGYk-------------------------TmhqhTAhAmsmfP

YP_00331559.1 rrciaGdDgrLTwLvLaSGYR-------------------------TvhqsSArAismfP

YP_00348965.1 rrcvaGdDgrLTwLvLaSGYk-------------------------TvhqsSArAismfP

ZP_07307227.1 KPAvFGdDRrmTnycLmeG-q-------------------------vvfqeTAvgyTaVP

ZP_08293056.1 rhveFsdDsmLTlfaLlhG-R-------------------------TvqqPSAfAfawmP

ZP_08232398.1 rhveFsdDsmLTlfaLlhG-R-------------------------TvqqPSAfAfawmP

BAJ27055.1 rqvvFsdDsmLTlfaklrG-R-------------------------TvqqPTAfAlsamP

YP_003491630.1 rPvmFsdDslLTlyaLlrG-R-------------------------avqqPSAvvfTalP

YP_00339317.1 sPAtFGdDRaLTnvvL-rdWk-------------------------vrYaaNAvshTvVP

YP_884889.1 rPStFGdDRsLTnyLL-rdWR-------------------------vlYaPdAqAyTnVP

YP_00107328.1 refrFatDRqLTgyvLgqvWkGqALKrRYADdPlVaDHDHPErRWlvgYVrSAhvwTtVP

ZP_06920913.1 gefrFatDRqLTgyvLgqkWkGRkLKaqYADSPFVternHPElpWrigYVqSAkvwTtVP

ADW02611.1 aPfrFatDRqLTgyvLgqaWRGKALKrRYAgSPFtae-DfaErRWrvgYVrSAkvwTdVP

ZP_05523903.1 aPfrFatDRqLTgyvLgqaWhGRALKdRhADSPFVrDHDyPElRWevgYtrAArvwTrVP

ZP_08389379.1 ----laEDqdLTiaiqrAGWR-------------------------vtYdPrAvAwTeaP

YP_00126324.1 ----laEDqdLTiaiqrAGWg-------------------------vhYdqyAvAwTeaP

WP_01217233. rPSnFGEDRHLTiLMLkAGFR-------------------------TgYVPgAvArTlVP

AAB51164.1 rPSnFGEDRHLTiLMLkAGFR-------------------------TgYVPSAvArTlVP

EMF57041.1 qPSdFGEDRHLTvLLLkeGlR-------------------------TeYVPdArAaTlVP

YP_004582102.1 rPSdFGEDRHLTiLMLkAGlR-------------------------TeYVPdAtAaTvVP

AEH05539.1 KqSdFGEDRHLTiLMLtAGYR-------------------------TeYVPSAlAaTvVP

YP_003610198.1 KrSdFGEDRHLTiLMLaAGYR-------------------------TeYVPNAiAsTvVP

YP001863657.1 KrSdFGEDRHLTiLMLaAGYR-------------------------TeYVPdAiAaTvVP

CCA66310.1 KrSdFGEDRHLTiLMLaAGYR-------------------------TeYVrdAvAaTvVP

ACB86875.1 KrSdFGEDRHLTiLMLaAGYR-------------------------TeYVrdAvAaTvVP

YP_00131473.1 KlSdFGEDRHLTiLMLkAGFR-------------------------TeYVPNAivaTvVP

ZP_07595040.1 KlSdFGEDRHLTiLMLkAGFR-------------------------TeYVPNAivaTvVP

NP768667.1 KPSdFGEDRHLTiLMLkAGFR-------------------------TeYVPdAiAaTvVP

YP_001984572.1 KPSdFGEDRHLTiLMLkAGFR-------------------------TeYVPSAiAaTvVP

ACT34131.1 KPSdFGEDRHLTiLMLkAGlR-------------------------TeYIPdAiAaTvVP

ACA23959.1 KPSdFGEDRHLTiLMLkAGlR-------------------------TeYVPeAiAaTiVP

ACT34140.1 KPSdFGEDRHLTiLMLkAGlR-------------------------TeYVPdAiAaTiVP

ACT34100.1 KPSdFGEDRHLTiLMLkAGlR-------------------------TeYVPdAiAaTiVP

YP_00141170.1 cKLpnwvRQrsRWikgwmqtyLVrMR----------------------------------

YP_00141184.1 DtLsgYItQrfRWA----------------------------------------------

ZP_07305288.1 ttLKktyRQrLRWARSww----wmLpfvyarlslkqLisptfgLlqLvitpvmLAwtvim

YP_00401411.1 DtwRlfcRQrLRWARnTwRsdLrALs----rrwvwrrpfLafSMvekavSgftLlvsptf

YP_00422371.1 gtfRafvkQrvRWSRnsFRcyLtAIR----hGwvfkVpLIsQitmmqILltPftmf-vAl

YP_00458210.1 DsLKafIkQrvRWSRnsYRcyLtAIy----kGwlwrqpLIcQisvlqILltPItmG-vAm

ZP_06415311.1 DnLRafIkQrvRWSRnsYRtyLtAIy----kGwlwrqpLVtQvsvlqIvltPVtmG-vAm

YP_00151150.1 DnLRafIkQrvRWSRnsYRtyLtAIy----kGwlwrqpLItQvsvlqIvltPltmG-vAm

YP_00331559.1 DtfsafvkQrvRWSRnsYRcyLtAVs----kGwlwrtpfVtkvtvlqILltPltmG-ltl

YP_00348965.1 ssfRafvkQrvRWSRnsYRcyLtALy----kGwlwrVpLVtkitvlqILltPVtmG-mAl

ZP_07307227.1 EKLphfLRQQvRWnkSfFRESLwAfRhqkKyrpafw--LtcmeLalwLvfgtaMf--ySm

ZP_08293056.1 DRwshhyRQQeRWfRgsFirgLwrIRfLPvLswgwwrqatGwiqiwLviSVfV----ylv

ZP_08232398.1 DRwshhyRQQeRWfRgsFirgLwrIRfLPvLswgwwrqatGwmqiwLviSVfV----yll

BAJ27055.1 EtLghhLRQQLRWmRgsairTcwrLRhLPlnGyafwLqaahlfLalagaAafl----wlc

YP_003491630.1 ERpshfLRmyLRWmRgstirSLwrMRyLPltGwaywaqLtrwfq--VaLStaVLAw-lli

YP_00339317.1 htMRqfLRQQLRWkRSwtRESLIlaRfvwRkypvaaLsayvgia--IaLvaPIvAv-rAi

YP_884889.1 EhLKqfLRQQLRWkkSwlREaprAMcavrRknpvMvVmfa------LsivLPlIApqvvm

YP_00107328.1 aRfRPfLkQQvRWkkSfiRNlcftgsfmwRrGfgaaalfyGhvL--fvavaPlMAv-rhl

ZP_06920913.1 aRfRPfMRQQvRWkkSfiRNlfftgtfmwRrGlgpaVlfyGhvL--wvLcaPlMAv-rhl

ADW02611.1 aRLgPlLRQQvRWkkSfvRNlfftgsfmwRrGpgaaalyyGhvL--wvLAaPVMAf-shl

ZP_05523903.1 sRpgsfLRQQIRWkkSfiRNlfftgRfmwRrGpaaaalyyGhaL--wviAaPVLvv-rhl

ZP_08389379.1 EsfKalakQryRWAfgT-------------------------------------------

YP_00126324.1 EsvRalakQrfRWAygT-------------------------------------------

WP_01217233. DgLaPYLRQQLRWARSTYRDTaLALRIkknLskYITfEIcaQNLGtaLLlVmtMis-lSl

AAB51164.1 Dg-sPYLRQQLRWARSTYRDTaLALRIkknLskYITfEIcaQNLGtaLLlVmtMis-lSl

EMF57041.1 ERLRPYaRQQLRWARSTFRDTaLAgRLLPRLGrYLlLDVaGQNLvpLLLvLtVLtG-cAQ

YP_004582102.1 ERLRPYLRQQLRWARSTYRDTLLAIRLLPRLGrYLmLDVVGQNLapLLLALtVLtG-fAQ

AEH05539.1 DKLlPYLRQQLRWARSTYRDTLLALRLLPRLngFLTLDtLaQNVGsLLLAVsVLgG-vvQ

YP_003610198.1 DRLgPYLRQQLRWARSTYRDTLLALRLLPRLdrYLTLDVIaQNaGsLLLAIamLSG-flQ

YP001863657.1 DKLwPYLRQQLRWARSTYRDTLLALRLLPRLdrYLTLDVIGQNVGsLLLAVsmLAG-llQ

CCA66310.1 DtLRPYLRQQLRWARSTYRDTLLALRLLPRLdrYLTLDVIaQNIGsLLLAIsmISG-flQ

ACB86875.1 DtLRPYLRQQLRWARSTYRDTLLALRLLPRLdrYLTLDVIaQNIGsLLLAIsmISG-flQ

YP_00131473.1 DtLKPYLRQQLRWARSTFRDTfLvLpLLrgLnpFLTLDVVGQNIGpLLLALsVvtG-lAh

ZP_07595040.1 DtLKsYLRQQLRWARSTFRDTfLALpLLrgLnpFLTfDVVGQNIGpLLLALsVvtG-lAh

NP768667.1 hsLRPYLRQQLRWARSTFRDTfLAwRLLPeLdgYLTLDVIGQNLGpLLLAIssLAa-lAQ

YP_001984572.1 nKLgPYLRQQLRWARSTYRDTLLgLRLLPnLhrFLTLDVVGQNLGpLLLALsVLtG-lAQ

ACT34131.1 DRLgPYLRQQLRWARSTFRDTLLALhLLPsLdrYLTLDVVGQNLGpLLLALsVLAG-lAQ

ACA23959.1 DRLgPYLRQQLRWARSTFRDTLLALhLLPsLdrYLTLDVVGQNLGpLLLALsVLAG-lAQ

ACT34140.1 DRLgPYLRQQLRWARSTFRDTLLALRLLPsLdrYLTLDVVGQNLGpLLLALsVLAG-lAQ

ACT34100.1 DRLgPYLRQQLRWARSTFRDTLLALRLLPsLdrYLTLDVVGQNLGpLLLALsVLAG-lAQ

YP_00141170.1 ------------------------------------------------------------

YP_00141184.1 ------------------------------------------------------------

ZP_07305288.1 tfLTlggryhnPgvalvylaVylvVrfgQsgLyvLmrpdmtARQrwhsw----lVgtPaa

YP_00401411.1 MtLal---lrhhWff---cgcLAcwwwLsraakALpHLrrQpss---------FfliPgw

YP_00422371.1 ayVvlatraehPllg---lglaivwlfVgrgIrgMsHLwrRped---------iVllPLv

YP_00458210.1 tyLgf-wmvrpqana---pLIavAwlLggraVrgLsHLreQpRd---------ifilPvv

ZP_06415311.1 tyfvl-wmfrpeana---pIIaicwlLLgrlIrgLsHLrehpRd---------ifilPLt

YP_00151150.1 tyfvl-wmfrpeana---pIIaiAwlLLgrfIrgLsHLkehpRd---------ifilPLt

YP_00331559.1 wyLaf-srlelttlg---iaaavgwlLLgrgIrgfsHLrrhpqE---------iVllPLl

YP_00348965.1 gyLlf-srleltgrg---vflVlvwlLVgrgIrgysHLrkhpqE---------lLllPLl

ZP_07307227.1 ViLpi----mkPaqfvnhigdylifmVLmgyLrnVrYLdfprRgmgfvKrfgmFLlaPLY

ZP_08293056.1 LwrplvaghgiPptv---vLVplAIgLaQgsryitvwrsdttgserLts----lIlsPMa

ZP_08232398.1 LwrplvlgggvPptv---vLVplAIgLaQgsryisvwrsdttgpqryas----lLlsPva

BAJ27055.1 LarpltlgpasPWhl---laVpvALSyVeClryltvrrhdhtprqqLat----FalaPLt

YP_003491630.1 Vepal-ygrTpPasf---lIVpflIgwaQalrylsiirsderirtrLit----wLlmPaa

YP_00339317.1 VwLpl----vegrga---pLVylAgiymlavaygLyYVacRrRyd--pR----wVygiaf

YP_884889.1 rafvv-----qPhfisqlpfwyfggvaaiavIygLfYrlhRpvkr--------wyqgiff

YP_00107328.1 Vwapa----nglYfl---tLlylcgvVtKgfawALafkisnpgnp---l----wryrlLm

ZP_06920913.1 Vwapy----hglWfl---tglylggvLLKgvVwgLafkadnpRst---R----wryrPLm

ADW02611.1 Vwapa----hgalfl---tLlylcgvVLKgcVwgLaYridhpgds---R----wryrPLm

ZP_05523903.1 Lwapl----hlagpl---tLlylggvVLKgcVwgLaYrldhpgdr---a----wrcrPLm

ZP_08389379.1 ------------------------------------------------------------

YP_00126324.1 ------------------------------------------------------------

WP_01217233. ttsgs----qtPvii---lgVVvgMSIIRCcsvAL-----iAKdf---R----FLyfivH

AAB51164.1 ttsgs----qtPvii---lgVVvgMSIIRCcsvAL-----iAKdf---R----FLyfivH

EMF57041.1 Ia-Ta----TvPWap---iLaItAaTtsnCafalw-----RSREa---R----FfgyaLH

YP_004582102.1 VaaTa----TiPWwp---iLVItAVTLIsCccaAw-----htRQa---R----FfafaLH

AEH05539.1 LvtTa----TvPWqa---cLtIASMTLVRstVaAI-----RARQl---R----FLgfsaH

YP_003610198.1 IaLTe----TaPWka---cfVIASMSVLRCsVaAV-----RSREv---R----FLgfsaH

YP001863657.1 IvLTa----SaPWqa---cflIASMTMIRCsVaAV-----RSREl---R----FLgfsaH

CCA66310.1 IvLTa----TaPWqa---cfVIASMTMVRCsVaAI-----RAREl---R-----------

ACB86875.1 IvLTa----TaPWqa---cfVIASMTMVRCsVaAI-----RARE----------------

YP_00131473.1 fiMTa----TvPWwt---iLIIASMTIIRCsVvAL-----hARQl---R----FLgfvLH

ZP_07595040.1 fitTa----TvPWwt---iLIIASMTIIRCsVvAL-----hARQl---R----FLgfvLH

NP768667.1 LlIdg----SiPWwt---gLtIAAMTtVRCcVaAL-----RAREl---R----FIgfsLH

YP_001984572.1 LaLTg----TvPWla---sLmIvAMTMIhCsVvAL-----RARQr---R----FLgfsLH

ACT34131.1 LaLTa----TvPWsa---vLmItSMTIIRCsVaAf-----RARQl---R----FLgfsLH

ACA23959.1 LaLTa----TvPWsa---vLmIASMTIIRCsVvAf-----RARQl---R----FLgfsLH

ACT34140.1 faLkg----TvPWsa---vLmIASMTIIRCsVaAf-----RARQl---R----FLgfsLH

ACT34100.1 fvLTa----TvPWsa---vVmIASMTIIRCsVaAf-----RARQl---R----FLgfsLH

YP_00141170.1 --------hPvrlYralgL-----------------------------------------

YP_00141184.1 ------------------------------------------------------------

ZP_07305288.1 vfmNLvLLcPtrywALfkLRdNaWqsRgltaktalpkgrhrapsEkllnv----------

YP_00401411.1 iLVS-FamsvIKigALvTvRRqrWlTRqvavengvvvrtAgaavEais------------

YP_00422371.1 TLViIFIsLPvKvYALfTMnKqgWlTRsadsqggegqteAslggEgqsaeslglv-----

YP_00458210.1 vVmiVivaLPIKtwAfisMnKqgWlTRradliggegqsdAsvrvsq--------------

ZP_06415311.1 vLmiIivaLPIKawAfvsMnKqgWlTRrsdliggegqtdAstrtgsaarpaavsa-----

YP_00151150.1 vLmiIvvaLPIKtwAfvsMnKqgWlTRrsdliggegqtdAstrtspaasprpata-----

YP_00331559.1 aLVvIFvaLPvKlYAfvTMnKqgWlTRhadqvggdgqsaktlapaaeqpvraera-----

YP_00348965.1 aLVvImIsLPIKlYAflTMnRqgWlTRtsdriggegqdsAslgsstrvtastwpe-----

ZP_07307227.1 gVIqLtLLtPLrfYALfTLhKgsWgTRqggvevsvagdhendvtEifeeedpysd-----

ZP_08293056.1 TLwSalILrPLrvwgMvTsgKmgWnTRqqvevtsq-------------------------

ZP_08232398.1 TLwSalILrPLrvwgMvTsaKmgWnTRqrvevtsq-------------------------

BAJ27055.1 mLwvLvvLraLrwYgaaTcaRTgWgTRgrvelhtapsppAgerhspehplpg--------

YP_003491630.1 vVgSwtvLrfLrwYgMaTcaRTgWgTRqngaevtltgpadeatladlpdedtvri-----

YP_00339317.1 cffyLcfmLwqtywAIlTaRsTsWgTRpatagqpalevgS--------------------

YP_884889.1 TMfytiILVlqmpYAMvTIRdSkWgTR---------------------------------

YP_00107328.1 aVLgsmLLswLlpYsLaTIRKgtWargaq-------------------------------

ZP_06920913.1 sLLSslvLswLlpYsLlTIRRgvWsrRls-------------------------------

ADW02611.1 sLfSsvLLawLlpYsLlTvRRgvWsrgaa-------------------------------

ZP_05523903.1 sLLSccvLawLlpYALlTLRRgvWsrsaa-------------------------------

ZP_08389379.1 ------------------------------------------------------------

YP_00126324.1 ------------------------------------------------------------

WP_01217233. saLNVlILtPLKlYALlTIRdSrWlsRess------------------------------

AAB51164.1 saLNVlILtPLKlYALlTIRdSrWlsRess------------------------------

EMF57041.1 TaLNIFLLLPLKaYALCTLsdAsWgsRilpaiaprghgndlpgtppparavtgpfpavrp

YP_004582102.1 TfINIFLLLPLKaYAICTLsnAsWesRviptaa---------------------------

AEH05539.1 TLINLFfLLPvKaYAL--------------------------------------------

YP_003610198.1 TLVNLlfLLPvKaYALCTLsnSnWlsRgvpaggc--------------------------

YP001863657.1 TfINLlLLLPvKaYALCTLsnSnWlsRgsaadcfehvdppghavttdaavstgtd-----

CCA66310.1 ------------------------------------------------------------

ACB86875.1 ------------------------------------------------------------

YP_00131473.1 TpINLFLLLPLKaYALCTLsnSdWlsRysapev---------------------------

ZP_07595040.1 TpINLFLLLPLKaYALCTLsnSdWlsRyaapev---------------------------

NP768667.1 TpINIcLLLPLKaYALCTLsnSdWlsRkv-------------------------------

YP_001984572.1 afINIFLLLPLKaYALCTLsnSdWlsR---------------------------------

ACT34131.1 TfINV-------------------------------------------------------

ACA23959.1 TfINIFLLLPLKaYALCTLsiA--------------------------------------

ACT34140.1 TfINVFLLLPLKaYALCTLsnS--------------------------------------

ACT34100.1 Tf----------------------------------------------------------

NodI alignment

YP_288522.1 ----MtsssqpArpepSpdpsadsppAIslrGvvKRfGDItAvnGLDLDVPqGvVLGLLG

YP_004581971.1 MvhgMplprteTlaTtTgpgdqpgesAIsmrnvvKRYGEVaAvDGLDLDVPvGvcLGLLG

YP_003396437.1 --------------------msapgsAIsirGvvKRfGaVtAvDGLDLDVPmGicLGLLG

ABK02511.1 ----MSnEElsSkqTrSqtrgdqmqyvItAEnLtKtYGDVtAvDGisfsVPaGEafGLLG

YP_830611.2 MpelvSnEElsSkqTrSqtrgdqmqyvItAEnLtKtYGDVtAvDGisfsVPaGEafGLLG

ZP_02379660.1 ----------------------msvapIdfQnveKRYGgklvvnGLsfrVhaGEcyGLLG

YP_620996.1 ----------------------msvapIdfrnveKRYGDklvvnGLsfsVkvGEcyGLLG

YP_001764868.1 ----------------------msvapIdlrnveKRYGDklvvnGLsfNVkaGEcyGLLG

ZP_03570919.1 ----------------------msvapIdfrnveKRYGDklvvndLsfhVhaGEcyGLLG

EGD05154.1 ----------------------msvapIdfrnveKRYGDklvvnGLsfhVqaGEcyGLLG

WP_037067103.1 -------------mTpSrqyegramvAIdlsGvsKaYGDkvvvDGLsfsVarGEcfGLLG

WP_032981191.1 ----------------------mssiAIelvGvtKsYrgkavvDGLsfNiasGEcfGLLG

YP_004584406.1 ----MSiDhsvSptasTpapapddppAldvrGLtyRfGEktAvDsLDLtlapGEIVGLLG

WP_027153181.1 ---------mndtspmgvpatglaqsAIsArGLvKtfGkLrAvDGiDLDVPrGiIfaiLG

YP_001134653.1 --------------------mpstdkAvvvEGikKsfGaVtALrdvsfDVgrGEVLGLLG

ZP_04748267.1 --------------------mrnndmAvvvrGihKafGtVvALDdvsfEVgrGEVIGLLG

YP_639994.1 ---------------mqdsrtansdksvvvkGisKsfGDVhAvrdvsfDVgrGEVVaLLG

WP_005476199.1 -----------------------mdsgIrmEnLtKsYGsVhALEGvsLDVPtGsVLGLLG

YP_004584319.1 -----------------------mdngIhtEnLtKKYGaVygLhGLnLqVsaGsVLGLLG

YP_004584642.1 -----------------------mtfAIhAEaLtKRfGDtqALtGiDLaareGsVLGvLG

YP_872099.1t --------------------mdgdelAIlvEGvrKsfGtVqALrGvDLaVPrGrVLGLLG

YP_003335843.1 -----------------------mppAvvAEGLvKKYGDVmALDGmDLsVPeGtVfGLLG

YP_004804588.1 -----------------------mpgAIyAEGLvKtfGDVrALDGvDLDVPeGtVLGLLG

ZP_06919434.1 -------------------methmpgAIyAEGLvKtfGDVkALDGvDLDVPeGtVLGLLG

NP_629115.1 -----------------------mpgAIyAEGLvKtfGDVrALDGvDLDVPeGtVLGLLG

YP_004583512.1 ---------------------msdkpAvlAhdLhKRfGtsvALaGLDLsVatGtVhavLG

YP_001509263.1 ---------------------mtdhpAvlvEGLrKsYGrhtALaGLDLlVgeGtVhGvLG

YP_003340614.1 ----------------mshseamnsysvvAEGLvKRfGgahALhGfDLaVreGtVcGLLG

YP_004818496.1 --------------------madsdpAIvvEGLrKtYrDkqALaGLDLtVPqGtVhavLG

NP_825830.1| -----------------------mtdAIvvEGLrKRYGDkaALDGLDLtVarGtVhGLLG

ADW04873.1 --------------------mstvsdAIvvEGvhKRfGEkrALDGLgfaVrgGtVhGvLG

ZP_08237769.1 -----------------------mtdAIvmDGvhKRYGEkrALDGLDLaVggGtVhGvLG

YP_288522.1 PNGAGKsTTmRILTaqstvDeGhvsVlGhpIpQKskwaRslmGVVpQhdnLDEeLTveEN

YP_004581971.1 PNGAGKsTTmRmLTaqsiaDtGRinVlGyDVpRaskqaRaamGVVpQednLDteLTaRqN

YP_003396437.1 PNGAGKsTTmRmLTsqaiPDeGtigVleyrlpddskqaRaemGVVpQldnLDveLscRqi

ABK02511.1 PNGAGKsTTmkmiggvsQrtSGslsimGlDpeshgpeVRahlGVVpQqdnLDEeLkvRdN

YP_830611.2 PNGAGKsTTmkmiggvsQrtSGslsimGlDpeshgpeVRahlGVVpQqdnLDEeLkvRdN

ZP_02379660.1 PNGAGKTTTlkmLlgityPDAGaislcGepVpaRArraRQRVGVVpQFdnLDpdfTvREN

YP_620996.1 PNGAGKTTTlkmLlgLahPDAGtislcGepVpsRArhaRQRVGVVpQFdnLDpdfTvREN

YP_001764868.1 PNGAGKTTTlkmLlgLahPDAGtislcGepVpsRArhaRQRVGVVpQFdnLDpdfTvREN

ZP_03570919.1 PNGAGKTTTlkmLlgLayPDAGaislcGepVpsRArhaRRRVGVVpQFdnLDpdfTvREN

EGD05154.1 PNGAGKTTTlkmLlgLahPDAGaislcGepVpsRArhaRQRVGVVpQFdnLDpdfTvREN

WP_037067103.1 PNGAGKsTiaRlvlgmMlPDAGKitVlGlpVpaRArlaRRgIGVVpQidnLDlefTvREN

WP_032981191.1 PNGAGKsTisRmilgmtsPDAGnisVlGvqVpRQArsaRaRIGVVsQFdnLDmefTvREN

YP_004584406.1 PNGAGKTTaIRVivTLLRPaAGsvlVfGvDaaRQpirtRRlmGyVpQmlsaDsgLTGREN

WP_027153181.1 PNGAGKTTlmRmLaTLsRPDAGsAtVmGhDlvQapheVRaaIamtGQFAsLDEdLTGREN

YP_001134653.1 PNGAGKTTTVnILSTLIKPDSGRAmiAGhDVvtdpagVRRalmltGQhAALDDlLTGREN

ZP_04748267.1 PNGAGKTTmVdILSTLtRPDgGsAtVAGhDVvsdpagVRRsImVtGQqvAvDDaLTGeqN

YP_639994.1 PNGAGKTTTVdILSTLtKPDSGKAtiAGfDVvseAssVRRsImltGQqvALDDmLTGREN

WP_005476199.1 PNGAGKTTaVkILTTLaRPDdGmAwVgGfDVaRepwqVRRRIGVsGQetAvDplLTGtqN

YP_004584319.1 PNGAGKTTTVnILTTLLKPDgGsAwVgGfnVaRhplqVRRRIGVsGQetAveplLTGaEN

YP_004584642.1 PNGAGKTTaVRILaTLLRaDSGRAtVAGfDVaRspqqVRQhIGltGQYAsvDEdLTGlEN

YP_872099.1t PNGAGKTTlVRILTTLLaPDgGRAlVeGyDVvtQAgaVReRIGlaGQatAiqaeLTGREN

YP_003335843.1 PNGAGKTTTVRILTTLLKPDAGhAtVAGfDVvgdAqrlRshIGasGQYAAvDDhLTGaEN

YP_004804588.1 PNGAGKTTaVRVLTTLLRPDSGQAvVAGvDVlKypneVRRsIGlsGQFAAvDEyLTGREN

ZP_06919434.1 PNGAGKTTaVRcLTTLLKPDrGsAvVAGiDVlKdpdaVRRsIGlsGQFAAvDEyLTGREN

NP_629115.1 PNGAGKTTaVRcLTTLLRPDSGKAvVAGiDVlRQpneVRRsIGlsGQFAAvDEyLTGREN

YP_004583512.1 PNGAGKTTaVkILTTLLRaDSGhAeVlGlDVsRRAgelRsRIGltGQYAAvDErLTGqEN

YP_001509263.1 PNGAGKTTaVRILaTLLaaDgGRAeVlGvDVlRQpqlVRpRIGltGQYAAvDErLTGlEN

YP_003340614.1 PNGAGKTTaVRILaTLLRaDgGRAtVAGlDVaaQAakVRQvIGlasQepAvDEiLTGREN

YP_004818496.1 PNGAGKTTcVRILaTLLRhDgGRAeVAGyDVlRdpdqVRyRIGlVGQhAAvDEeLsGRqN

NP_825830.1| PNGAGKTTaVRVLTTLLRPDeGRveVAGrDVlRRAydVRlRIGlLGQhAALDEeLgGRqN

ADW04873.1 PNGAGKTTaVRVLTTLLRyDgGRAeVAGfDVrsQAaeVRRRIGlLGQhAAvDEkLgGRqN

ZP_08237769.1 PNGAGKTTaVRImSTLLRqDAGRvtVAGlDVreRAgeVRRRIGlLGQhAAvDEqLgGRqN

YP_288522.1 LrsFtyLYrvprRerdAavEraLriahLEhrrheftEklSGGMRRRLliArgLVhrPRVL

YP_004581971.1 LavFaRLYrvpARrrRAavEQaLalarLnDrADtvtEklSGGMRRRLliArALVhrPRlv

YP_003396437.1 LtvFaRLYrvpkhdraAavEraLsiaNLvDrADtrVdllSGGMRRRLliArgLVhrPRlv

ABK02511.1 LlvYGRyFGLpmsylRpKADELLEfaqLtDkAkskVdalSGGMkRRLtiArsLinEPRIL

YP_830611.2 LlvYGRyFGLpmsylRpKADELLEfaqLtDkAkskVdalSGGMkRRLtiArsLinEPRIL

ZP_02379660.1 LlvFGRyFGvSAhdARAlvpsLLafakLEnkADakVselSGGMkRRLtLArALVnDPdVL

YP_620996.1 LlvFsRyFGmSAqaARAlvapLLEfakLEnkADakVgelSGGMkRRLtLArALVnDPdVL

YP_001764868.1 LlvFsRyFGmSAqaARAlvppLLEfakLEnkADakVgelSGGMkRRLtLArALVnDPdVL

ZP_03570919.1 LlvFsRyFGmSAhaAsAlvqpLLEfakLEnkADakVgelSGGMkRRLtLArALVnDPdVL

EGD05154.1 LlvFsRyFGmSAqaARAlvqpLLEfakLEnkADakVgelSGGMkRRLtLArALVnDPdVL

WP_037067103.1 LlvYGRyFGmStReiKAvmpsLLEfarLEskvDarVsqlSGGMkRRLtLArALinDPQlL

WP_032981191.1 LfvYGRyFrmkAReieAivpsLLEfarLEnkADtrVadlSGGMkRRLsLArALinDPQIL

YP_004584406.1 vaLFagLFdvprRhrRARvDQaLaamgLtDvADRlaRTYSGGMvRRLELAqALVnaPRlL

WP_027153181.1 LvLlaRLwGfrgRaAKARADDLLaaFgLsEAAtKqVRdYSGGMRRRLDiAAsLivtPgVL

YP_001134653.1 LlMFGRLqGLkkKvAKqRAqELLEqFDLvgAgDRaVgnYSGGMkRRiDiAcgLVvrPeVv

ZP_04748267.1 LvLFGRLYGLSksaARrRsqELLEqFgLmhAAkRrVsTYSGGMRRRiDiAcgLVvqPQVa

YP_639994.1 LvLFGRLqGLAkKaAeARADELLtaFDLQhAANRrlsTYSGGMRRRiDiAcgLVvrPeVv

WP_005476199.1 LewFGRigrLSrRqARdRArQLLEiFDLtEvAgRlaRTYSGGtRRRLDLAvsLVsrPeIL

YP_004584319.1 LeLFGRLhrLSrRhARgRAqELLEmFDLtaAAgRlaRTYSGGMRRRLDLAisLikrPsIL

YP_004584642.1 LvMiGqLldmrAaeARrRAvELiEwFDLvEAAgRvaKTYSGGMRRRLDLAAsLVgrPsVi

YP_872099.1t LeiiGRLYhLpramARdRAaELLaaFgLEEAADRpaKTYSGGMlRRLDLAAsLigsPsVL

YP_003335843.1 LeMvGRLYhLgtKrsKeRArELLEcFDLtqAADRpVQgYSGGMRRRLDLAgALVaNPpVL

YP_004804588.1 LrMvGqLYqmSgRdAKkRADQLLErFNLaDAADRtaKTYSGGMRRRLDLAAALVvsPpVm

ZP_06919434.1 LqMvGRLYqmkAKpAKARAaELLEqFDLaDAADRptKTYSGGMRRRLDLAAALVvsPpVm

NP_629115.1 LhMvGqLYqmkgKaAKARAaELLdqFhLsDAADRptKTYSGGMRRRLDLAAALVvsPpVm

YP_004583512.1 LeMFGRLYrLSvvsARARAEtLLErFDLtDAArRqagTYSGGMRRRLDLAAsLilaPpmL

YP_001509263.1 LeMFGRLYrLSARtARARAaELLErFDLaEAAgRqaKTYSGGMRRRLDLAAsLimaPaVL

YP_003340614.1 LvMwGRLYhLdAKrAarRADELLEqFgLtEAAgKrIKhYSGGMRRRLDLAAtfiqaPRVL

YP_004818496.1 LeMFGRLYhLggRqAaARADELLErFgLagtgtKaVsqYSGGMRRRLDLAAsLVmrPRIL

NP_825830.1| LeMFaRLhhLrtRhARvRAgELLdrFgLaDtgpKaVKhYSGGMRRRLDLAAsLiaEPeVL

ADW04873.1 LeMFGRLYhLgARrAgqRADELLErFgLagtgrKaVErYSGGMRRRLDLAAsLitDPeVL

ZP_08237769.1 LeMFGRLYhLgARrAgSRADELLErFgLaDtgrKaVKrYSGGMRRRLDLAAsLitDPdVL

YP_288522.1 lLDEPTvGLDPqvRQeLWglIsALrde-GvTVLmsThYiEEAeRLsDeVAlmakGRIVer

YP_004581971.1 lLDEPTvGLDPqvRQeLWslIagLgAd-GvTVLmsThYiEEAeRLAqdVAimsrGRVIAr

YP_003396437.1 lLDEPTvGLDPqvRQeLWslIdALrAe-GTTVLmsThYiEEAeRLADtVAVmarGRIIAQ

ABK02511.1 lLDEPTTGLDPqARhiLWDrlfrLkeq-GvTliLTThYmdEAeqLcDRliVVDkGRImAE

YP_830611.2 lLDEPTTGLDPqARhiLWDrlfrLkeq-GvTliLTThYmdEAeqLcDRliVVDkGRImAE

ZP_02379660.1 vLDEPTTGLDPqARhlMWErlRSLLAh-GkTILiTThfmEEAeRLcDRlcVIeeGRkIAE

YP_620996.1 vLDEPTTGLDPqARhlMWErlRSLLAr-GkTILiTThfmEEAeRLcDRlcVIeeGRkIAE

YP_001764868.1 vLDEPTTGLDPqARhlMWErlRSLLAr-GkTILiTThfmEEAeRLcDRlcVIeeGRkIAE

ZP_03570919.1 vLDEPTTGLDPqARhlMWErlRSLLAr-GkTILiTThfmEEAeRLcDRlcVIeeGRkIAE

EGD05154.1 vLDEPTTGLDPqARhlMWErlRSLLAr-GkTILiTThfmEEAeRLcDRlcVIeeGRkIAE

WP_037067103.1 vmDEPTTGLDPhSRhlIWErlRSLLAr-GkTIiLTThfmEEAeRLcDRlcVlerGRnIAE

WP_032981191.1 iLDEPTTGLDPhARhlIWErlRSLLAq-GkTILLTThimEEAeRLcDRlcVlegGvkIAE

YP_004584406.1 iLDEPTvGLDPiARdsvWEhVlrLreehGmaVLLTThYmEEADvLcDRVAlmhaGRVrvt

WP_027153181.1 FLDEPTTGLDPnARKdvWgmIRgLaea-GvTILLTTQYLEEADqLAaRIAVIDHGRkIAE

YP_001134653.1 FLDEPTTGLDPRSRQaIWElVtdfkea-GiatLLTTQYLEEADlLsDRIiVIDkGtVIAE

ZP_04748267.1 FLDEPTTGLDPRSRQaIWDlVaSfkkl-GvatLLTTQYLEEADaLsDRIilIDHGtIIAE

YP_639994.1 FLDEPTTGLDPRSRQtIWDlVsgfkdv-GiatLLTTQYLEEADaLsDRIiVIDHGgIVAE

WP_005476199.1 FLDEPTTGLDPRSRtatWDiVReLVdg-GTTlLLTTQYLEEADRLADRVAVIDrGRLlAE

YP_004584319.1 FLDEPTTGLDPRSRtatWDlIReLVgs-GvTlLLTTQYLEEADqLADlIAVVDqGcLIAE

YP_004584642.1 FLDEPTTGLDPvrREqMWDvVRSLVhd-GSTVLLTTQYLEEADaLADeItVIDrGRVIAh

YP_872099.1t FLDEPTTGLDPRSRiaMWQiIRrLVAt-GTTlfLTTQYLEEADeLADeIvVIDHGRVIAa

YP_003335843.1 FLDEPTTGLDPRARagLWDvIseLVAg-GTTlLLTTQYmEEADRLADRIAVVDHGRVIAl

YP_004804588.1 FmDEPTTGLDPRnRQqLWEvIQdLVAg-GTTlLLTTQYLEEADhLAhdIcVIDHGkVIAr

ZP_06919434.1 FmDEPTTGLDPRnRQlLWEvIKqLVSg-GTTlLLTTQYLEEADhLAhdIAVVDHGRVIAt

NP_629115.1 FmDEPTTGLDPRnRQeLWEvIKqLVAg-GTTlLLTTQYLEEADhLAhdIAVVDHGRVIAr

YP_004583512.1 FLDEPTTGLDPRSRiqMWkvIeeLVrd-GTTVLLTTQYLEEADqLArRISVIDgGRVIAE

YP_001509263.1 FLDEPTTGLDPRSRQaMWrvIadLVrd-GTTVLLTTQYLEEADqLADRVSVVDtGRVIAE

YP_003340614.1 FLDEPTTGLDPRnRnevWQaVRTLVtn-GSTVLLTTQYLdEADqLADqIAVlreGRVIAD

YP_004818496.1 FLDEPTTGLDPRgRtevWnaVRSLVdg-GTTVLLTTQYLEEADqLADRVcVIDgGRaIAD

NP_825830.1| FLDEPTTGLDPRgRaevWaaVRSLVgg-GTTVLLTTQYLEEADqLADRISVVDHGRVVAD

ADW04873.1 FLDEPTTGLDPRgRaevWnaVRSLtAg-GTTVLLTTQYLEEADqLADRISVVDgGRVVAE

ZP_08237769.1 FLDEPTTGLDPRgRtevWDaVRSLVgg-GTTVLLTTQYLEEADRLADRISVIDgGRtVAE

YP_288522.1 GePaDL-iAKyaGktVEeyqigaegveelekVI-----hhagfttrrTgstLsVlr----

YP_004581971.1 dTParLIdAhaGrtaaEyygpppklaeiAarae------aagfttrrTgpsvsVlr----

YP_003396437.1 GrPaEL-vvehaGseahmVygsparLaelrAea-----daagwvtrrSgpaLaIlh----

ABK02511.1 GsPssLIreystrEvLElrfgsernatigaeLe------gigerLEtlpdRvlIya----

YP_830611.2 GsPssLIreystrEvLElrfgsernatigaeLe------gigerLEtlpdRvlIya----

ZP_02379660.1 GaPhaLIeSeIGcDvIEIygaDpaaLrdelAaf--------aertEiSgetLfcyV----

YP_620996.1 GaPhaLIeSeIGcDvIEIygpDpaaLrdelSaf--------akhtEiSgetLfcyV----

YP_001764868.1 GaPhaLIeSeIGcDvIEIygpDptaLrdelSaf--------akhtEiSgetLfcyV----

ZP_03570919.1 GaPhaLIaSeIGcDvIEIygpDpiaLrdevApL--------aqrtEiSgetLfcyV----

EGD05154.1 GaPhaLIeSeIGcDvIEIygpDpaaLrdelApL--------aqrtEiSgetLfcyV----

WP_037067103.1 GrPqaLIdelIGcEvIEIyggnpheLqslirpy--------anrIEvSgetLfcya----

WP_032981191.1 GrPfDLIKeQIGcpvIEIyggDpqeLsllikpn--------arrIEiSgetLfcyt----

YP_004584406.1 GvPgEL-KAeLGa---------rasLedvfrhh-------tgdaLagNgneagdfr----

WP_027153181.1 GTsrEL-KAatGsgfLhValaDvarLdqAaSIL--errlehpvqrsaegaeLsVla--gt

YP_001134653.1 GTaDQL-KeRtGGtycEIVprqlsqLrevarILGpllpaahrdaLsdTsdRisMpa-PDG

ZP_04748267.1 GTaNEL-KhRaGdtfcEIVprDlkdLdaivAaLGsllpeqsraiLtpesdRitMpa-PgG

YP_639994.1 GTaDEL-KeRtGGsycEIVprDlndLpaivdaLGslipeqnraaLtaAsdRiaMpa-PDG

WP_005476199.1 GTvEEL-KtRtsdDrIEIVasslelvpkArtVV--srfstsppvVvwSeRRiwama-Phe

YP_004584319.1 GTvEEL-KtRtsdDrIEIVlrDhelLplAatIL--drfatseavVtrSeRRiaVma-Phk

YP_004584642.1 dTPDgL-KSvVGGqrLsVrpaDpgqLdtvrqLL----aeitasrpEpAgRdhltva-vDG

YP_872099.1t GTaaEL-KdRVGGDvLEfIvpDvehLdaAvAaV--aglsdspphVDretQQisLaVgsrG

YP_003335843.1 GTaDEL-KdQVGGDrIaltvtDpadLetArrLL--aplavgemqaDtTalQvtVpV-nNG

YP_004804588.1 GTsDQL-KARtGGErVEVVvhqsdqiepArSVL--aaygkgeiaVsphtRKLtVpV-tgG

ZP_06919434.1 GTsDQL-KARtGGErVEVVvherdhiqaAseIL--rgfgkgdttVEdhmRKLtVpV-tgG

NP_629115.1 GTsDQL-KARtGGErVEVVvhDrgrmatAsdVL--agfgkgsttVEehtRKLtVpV-tgG

YP_004583512.1 GTaDDL-KSQVGGDrLdIVitrggdvdaArgVL--arfasgevsVDvdrRRLsapM-nrG

YP_001509263.1 GTtDDL-KrQIGGDrLdItvaegcdLggAaeVL--arsgtgaatVDadeRRvsVpV--sG

YP_003340614.1 dTPErL-KSsIGGDrLdlVlrDqrdLreAgdVL----aafgppvaDpdaRRLtapV-tDr

YP_004818496.1 GTaDEL-KARLGGDrIEVVlrDagqLsaAaSVV-graaragevetDpdrRRLgarV-tDr

NP_825830.1| GTaDQL-KARtGGDrIdVVlrDagqLgaAvALL---pvgtsgvrVDrdrRlLsapV-tDr

ADW04873.1 GTaDrL-KAmVGGDrIdVVirDtdrLgeAaALL------gegvtVDrdrRlLgapa-PDr

ZP_08237769.1 GaPDEL-KAlVGGDrVdIVlrDasrLaeAgALL-----ggadlvLDadrRRigapa-PDr

YP_288522.1 aeeLpdSLRdrlgtGmr------RasnLeDVFvtLTGetvE-------------------

YP_004581971.1 aenMpaeLddrlgiGVr------RaaTLeDVFvaLTGervE-------------------

YP_003396437.1 aerhngtLpe----Ger------RaaTLeDaFvmLTGeeiE-------------------

ABK02511.1 -hdgeaALeqVsarGlrpltslvRRssLeDVFLRLTGRslv-------------------

YP_830611.2 -hdgeaALeqVsarGlrpltslvRRssLeDVFLRLTGRslv-------------------

ZP_02379660.1 sdpdplnaRlkgrAGlrylh---RpanLeDVFLRLTGRemQ-------------------

YP_620996.1 idpepltaRlkgrpGlrylh---RpanLeDVFLRLTGRemQ-------------------

YP_001764868.1 idpeplttRlkgrpGlrylh---RpanLeDVFLRLTGRemQ-------------------

ZP_03570919.1 sdpeplcaRikgrAGlrylh---RpanLeDVFLRLTGRdmQ-------------------

EGD05154.1 tdpeplSaRikgrAGlrylh---RpanLeDVFLRLTGRdmQ-------------------

WP_037067103.1 sdpqqvrvqlqerAGlrVlq---RpPnLeDVFLRLTGRemE-------------------

WP_032981191.1 pdpeqvraqlrghwGlrLlE---RpPnLeDVFLRLTGRemg-------------------

YP_004584406.1 --------------GVQhtrrAaRRvg---------------------------------

WP_027153181.1 akaaneALadLvaAGIELsDfSmgqPsLeeVFfaLTGqpSl-------------------

YP_001134653.1 pntLmqALhlLseAdIELsDIALRRPsLDeVFLaLTGdhpK-------------------

ZP_04748267.1 trtLieAaRrIdeAnIELaDIALRRPsLDDVFLsmTtdpSE-------------------

YP_639994.1 pntLveALsrLnsAsIQLtDIALRRPsLDDVFLaLTadsesaaaddsdtaddGkskelvg

WP_005476199.1 rglLtrlMRdMdaAGIELdDVeLRRPTLDDVFLQLTGRpAE-------------------

YP_004584319.1 pglLtqvLReMdvAaIELeDVeLRRPTLDDVFftLTGRpmD-------------------

YP_004584642.1 devLaevIarLrsAGVrVtELSLhlPsLDeVFftLTGgrtg-------------------

YP_872099.1t saaLveAvRaLdsAGVpIegLvmRRPsLDDVFLavTGhaAv---------sesPpdrrrr

YP_003335843.1 aasLtnALarLaaeGVtVrDagLRRPTLDDVFLtLTGheAa-------------------

YP_004804588.1 aklLaevIRdLdtrGVEIdDIgLRRPTLDDVFisLTGhaAE-------------------

ZP_06919434.1 aklLaevIReLdtrGIEIdDIgLRRPTLDDVFLsLTGhvAE-------------------

NP_629115.1 aklLaevIReLdvrGIEIdDIgLRRPTLDDVFLsLTGhvAE-------------------

YP_004583512.1 agvLpdlvRgVdevGVaLeDIALhRPTLDDVFLaLTGhhAr-------------------

YP_001509263.1 agiLaevvRgLdeiGVtIvDVALhRPsLDDVFmtLTGhgAa--------parGPagatgp

YP_003340614.1 mhaLgqvMReLeakGIEVeDVALRRPTLDeVFLKvTa-----------------------

YP_004818496.1 vaaLtetvRaLaaAGIEaeDIvLRRPTLDeVFLhLTGepAt-------------------

NP_825830.1| maaLsgvvRaLeaAGIEaeDVALRRPTLDeVFLRLTGRa---------------------

ADW04873.1 mraLtrtvRaLeeAGIEaeDIAvRRPTLDeVFLsLTGsghE-------------------

ZP_08237769.1 maaLtrAvRvLaeAGIEaeDIAvRRPTLDeVFLsLTGRpAa-------------------

YP_288522.1 ------------------------

YP_004581971.1 ------------------------

YP_003396437.1 ------------------------

ABK02511.1 -----------------------d

YP_830611.2 -----------------------d

ZP_02379660.1 -----------------------d

YP_620996.1 -----------------------d

YP_001764868.1 -----------------------d

ZP_03570919.1 -----------------------d

EGD05154.1 -----------------------d

WP_037067103.1 -----------------------k

WP_032981191.1 ---------------------kyq

YP_004584406.1 ------------------------

WP_027153181.1 ---------------gesrKDdkp

YP_001134653.1 --------ardesedplvaaDaya

ZP_04748267.1 -------------slshlasagmr

YP_639994.1 SsagGsgrhrrngrgslttsDlas

WP_005476199.1 --------------aprhgEDvvr

YP_004584319.1 -------------vkaqhematve

YP_004584642.1 -------------sraeieEEpaa

YP_872099.1t SrgrGrggseperdevvseQggqr

YP_003335843.1 ----------------envKEhsr

YP_004804588.1 ---qaKadeagpteadrgrKEser

ZP_06919434.1 -----------akdeesdkKEttk

NP_629115.1 --aadEtngsaddadheqeKEtak

YP_004583512.1 ----dEgtatsphestttedlvtt

YP_001509263.1 ggpgGDdgppgprtggderdlvka

YP_003340614.1 ------------------------

YP_004818496.1 ---------aerskadtaeEEtpv

NP_825830.1| ------------------------

ADW04873.1 -----------sgdlasagtEvav

ZP_08237769.1 ------errtaeqatratktEaaa

NodJ alignment

AGZ53017.1 --------------mmpvTtqevglaParsqdpenS-------------vrllieqtllq

YP872505.1 -----------msfAlrilpygafgrftgrR------------------svalverSlmv

YP872504.1 -----------------mSgmAaAaePlgtargn---------------lpaamrrtWrA

NP768671.1 ----------------mddgyAs--------------------------vmpanayNWtA

YP001770638.1 -----------------mTgdfaA-------------------------alpanawNWlA

NP659764.1 ----------------mdegcAaA--------------------------lpanafNWIA

YP002499215.1 ---------------migegyAaA--------------------------lpsnawNWlA

ZP09087353.1 ----------------mgegcAtA--------------------------lptnawNWIA

YP004686285.1 --msEqDpraDtttApaaagghaAaaPrqrypqp---------------llprnlrNWmm

YP558549.1 --------------mdarTyetpgdtPsrqdtfa---------------afpanatNWIA

YP001895399.1 --------------mdarTyetpgdtPskqeafa---------------aspanatNWIA

YP001808214.1 ------------MdArnySatApSapPresRfai---------------alpanatNWIA

YP001764867.1 ------------MdvrnySattpSaplresRfai---------------ampanasNWIA

YP_004581970.1 ------------MSttddagantAganttggtgetrr------------lrwvepaalag

YP003396438.1 -------------------msAtAarPkprRrrm---------------rrieptavfgv

YP_004584405.1 masstPavppagLASapgerdprtagnaadRawpgglvdddfdtsrlgaarlllrrlatl

AAZ55486.1 ------------Mti----------------------------------pttpltDtitv

WP_037627920.1 -----meTpsgdqAvivrTdrAspatPrrkRkrpkSp------------pvefliDvhml

YP_004584320 ---MKPNTiiDgaAAsngekqvvAgrhrstRshhvTrgWaGpGKPEgiIpvtfliDvyll

YP_004583032.1 ---MKPNTiiDgaAAsngekqvvAgrhrstRshhvTrgWaGpGKPE--IpvtfliDvyll

BAC70090.1 --------------------------------------------------mnllaDgWtl

ACZ90809.1 --------------------mtvltsPlgr-------------------lrwtltDgltl

YP001133205 -----------mtAvdsqThtpvqprPvpiR------------------ptnlaqqSWIm

ABG10364.1 ------------MtAvesrqsdqArrPlthRtn-------------------lvqqSWIm

WP_011692931.1 ------------MSA----------------------------------itaaarDSsVi

YP004583513 ------------MStisgnaaAvAvrPvegRsr----------------lvwsllDaWVv

YP480579 -------------------mstieltPagrgr-----------------lswavaDaWVm

YP001509262 -------------------mttldltPvarRsr----------------ltwellDaWVm

ZP06412839 -------------------mstldlsPvpgRgr----------------laweaaDaWVm

NP_629116.1 ------------------msaAtdaaPvtasanp---------------igqsvrDSlVi

AEM81020.1 ------------MAvveeTgatvAaaPrprgg-----------------plrsvrDSlVv

EDY58361.1 -----------msAvtdtarvApAtnP----------------------isqsfrDSmVv

YP004804589 ----------------mtTtptsefaPrarRga----------------iaqsvvDSlVv

ADW03735.1 ---------------msavtdApdiaPlksRga----------------vgrsvaDSlVv

AGZ53017.1 tkRlLI-rWar-dyvTvIgaIVlPILFMvvlniVlGnlayavt---hdS-----glysiv

YP872505.1 yrh----tWii-----IVsgffEPLfyLLsiGvgvGrlVphlsya-Ghp---iAYtAFvA

YP872504.1 y-RywVtsYRrtwrgSvVsSIVnPVLyLaalGvglGtlVhagpaThGlS-----YlqFiA

NP768671.1 VwRrnylaWRkvAlaSLlgNLadPItnLfglGFglGliVgrve---GtS-----YiAFLA

YP001770638.1 VwlrnyqaWkkIAPaSIlgNLadPtIyLvglGFgvGlmIdqve---Gsp-----YiAFLA

NP659764.1 IwRrnylaWkkvAlaSIVgSLadPmIyL--FGlglGliVgrvd---GtT-----YvAFLA

YP002499215.1 VwRrncqaWkkvAlaSvlgNLadPmIyL--FGlgFGvmlgqve---Gtp-----YiAFLA

ZP09087353.1 VwRrnymaWkkvAlaSLlgNLadPLIyL--FGlgFGlvVgrvd---GtS-----YvAFLA

YP004686285.1 VwyrnymVWkkLAipSMIgNLadPmIyLfglGlglGlmVgqvn---GvS-----YiAFLA

YP558549.1 VwRrnylVWRkLAiaSMfgNLadPmIyLfglGFglGlmVghvd---GvS-----YiAFLA

YP001895399.1 VwRrnylVWkkLAiaSMfgNLadPmIyLfglGFglGlmVghvd---GvS-----YiAFLA

YP001808214.1 VwRrnylVWRkLAlaSMfgNLadPmIyLfglGFglGlmlghvd---GvS-----YiAFLA

YP001764867.1 VwRrnylVWRkLAlaSMfgNLadPmIyLfglGFglGlmlghvd---GvS-----YiAFLA

YP_004581970.1 VitHdItLfRrywPsTsfsavVEPtIyLLaFGwgFGslVsvva---Gyr-----YveFLg

YP003396438.1 MsRDat-IftrywktTtfsSvVdPtIyLLaFGFgFGalVssvn---Gld-----Ykeyi-

YP_004584405.1 slveLqkLrhd--rveLytraVQPaLwLLvFGvtFnrvhaipt---Gsv----pYldyLA

AAZ55486.1 fvReLrpVlRd--PfTvVfSLVQPLVFLalFapLlpegls------GeS-----tlqwfv

WP_037627920.1 tvRlLMrMrRe--tdqLVygIVQPaVFtLmltYVlGnAVklpp---Gtr-----Yadyai

YP_004584320 trRlLMrMphq--PdlIVySIVQPaIFtLamiYLFGkAItlpG---ead-----YtdFvi

YP_004583032.1 trRlLMrMphq--PdlIVySIVQPaIFtLamiYLFGkAItlpG---ead-----YtdFvi

BAC70090.1 tqRrLfrLrhe--PgTLltmLmmPsgFvvlFGFVFGgAIgvpG---tsN-----YreFmm

ACZ90809.1 VgReLgrLrqe--PgqIVaaLVfPVImvvlFGYVFGsAIqvpG---Ggd-----YreyLm

YP001133205 VkRNMIhtkRm--PemLsdvtaQPImFvLlFaFVFGaSItntG---GaS-----YreFLl

ABG10364.1 VkRNMIhtkRm--PemLsdvtaQPImFvLlFaFVFGaSItntG---GaS-----YreFLl

WP_011692931.1 arRNLInVlRt--PgALVtgIVQPVmFvLllGFVFGgAlg------Gdq-----YrSFLi

YP004583513 crRNLIqtWRv--PelLVfStIQPVmFvLlFvYVFGgAInvgp---Gvd-----YvdFLm

YP480579 crRNLIqtWRi--PelvIfatIQPVmFvLlFtYVFGgAInvgd---Gvk-----YvdyLm

YP001509262 crRNLMktWRv--PelLVfatIQPVmFvLlFsYVFGgAIdvgp---dld-----YvdFLm

ZP06412839 crRNLVqtWRv--PelLVfatIQPVLFvLlFtYVFGgAInvgp---Gve-----YvdyLm

NP_629116.1 arRNLIrMtRi--PemvlfgIIQPVmFvIlFtYVFGgSIkvgs---ttd--SdvYkdFLm

AEM81020.1 akRNLLrMmRi--PemvVfgLVQPImFvvlFsYVFGgAImvpG---qaSadAtvYreFLm

EDY58361.1 akRNLIrMsRi--PemIIygLIQPImFvvlFtYVFGgSmqigssTsatd-----YknFLm

YP004804589 arRNLIrMiRi--PemvIfgLIQPImFvvlFtYVFGgSIkvgG---tiS--pqAYreFLm

ADW03735.1 arRNLIrMaRi--PemvIfgLVQPImFvvlFtYVFGgSIkvgs---slS--StAYkeFLm

AGZ53017.1 plIsigaaitgstFvaId-LmrErs-mGlLsRlwvvPvhRasgLIaRilAnaIRtlfttl

YP872505.1 pamlAssaMngAiYDstFnIfhkLkyarvyegvlAtPmTaldVaaGeilwALgRgfIyav

YP872504.1 pGLlAaTaMqvASiEatYpVlasVkwvpmyDamlAtPLTvGdVfaGhllwmLtRigLtss

NP768671.1 AGmvAisaMtSAtFEtLYAafarMdVkrTwelFt--qLTlGdIvLGelvwAasKSvLag-

YP001770638.1 AGmvAtaaMtSAtFEiIHAafarMHaQrTweailytqLTlGdVILGelawAatRAfLag-

NP659764.1 gGmvAtsaMtSAtFEtIYAaftrMHaQrTweamlytqmTlGdIILGelawAasKAfLag-

YP002499215.1 AGmvAtsaMtSAtFEtIYAafarMHaQrTweailctqLTlGdIvLGelawAatRAfLag-

ZP09087353.1 gGmvAtsaMtSAtFEtIYAafarMHaQrTweailytqLTlGdIvLGelawAatKAfLag-

YP004686285.1 AGttAssVMmSASFEsMYSafsrMHVQrTweaimhaPLTlGdVvLGeiawAasKAvLsgl

YP558549.1 AGtvAssVMmSASFEsMYSgfsrMHVQrTweaimhtPLTlGdIvLGeviwAasKSiLsga

YP001895399.1 AGtvAssVMmSASFEsMYSgfsrMHVQrTweaimhtPLTlGdIvLGeviwAasKSvLsga

YP001808214.1 AGtvgssVMmSASFEsMYSgfsrMHVQrTweaimhtPLSlGdIvLGeivwgasKAlLsgv

YP001764867.1 AGtvgssVMmSASFEsMYSgfsrMHVQrTweaimhtPLAlGdIvLGeivwAasKAlLsgg

YP_004581970.1 tGVvAsaVLFasAFasMFqtfvrrtyQklygavlAaPvdvpeIvLGealwiaaKAgvygc

YP003396438.1 -GtvAtaVLFSsAFpaMYgtfikyrfQrTyDaFlAaPvdvdeIvtsevlwigLRAgvygn

YP_004584405.1 pGIlAQsaLFisiFyGIq-IiwDrd-aGvLaKlmvtPtpRtAlvtGKafAAgVRAlIqvf

AAZ55486.1 pGIivmscLFatmstGan-LlfEIq-lGsheRmlvsPLrRsslIVGRalkeIVpvlaqtv

WP_037627920.1 sGLlAQTVvttAivtata-VAyELs-EkmIDRlRTLPvSRlsILascTnASLVRSlItvv

YP_004584320 AGLlAQTVvmgAAsatsggIAfELs-QkTIDRlRTLPLSRlAILaGcTnAgVIRAfItvf

YP_004583032.1 AGLlAQTVvmgAAsatsg--AfELs-QkTIDRlRTLPLSRlAILaGcTnAgVIRAfItvf

BAC70090.1 pGLFvmTtgtalStamVe-VAtDqa-rGvMeRlRSLPthRvsVpLGqSgAegIlgaygla

ACZ90809.1 pGLFAm-VtFSAwlgvMtrMAtDas-rGvMDRFRSMPmARlAVpfGqTgAdLLtglLmla

YP001133205 p--qAQTIvFSAfvvas---taDVe-k--IDRFRSLPiSRssVLIGRSiASVIhSsLgal

ABG10364.1 pGIqAQTIvFSAfvvasg-ItaDVe-kGiIDRFRSLPiSRssVLIGRSiASVIhSsLgvf

WP_011692931.1 gGIlAQTltFnASFtaVY-LAkDLq-lGlIDRFRSLPmSRvAVILGRTtsdLstSvLsvv

YP004583513 p--FiQTaiFgsmvtGVg-LAeDrq-rGlIDRFRSLPmSRGAlLaGRTlsdLfRnvfvva

YP480579 p--FlQTViFgsmltGVg-LAeDrq-rGlIDRFRSLPmSRsAlLtGRTlAdLaRnvfivv

YP001509262 pGVFvQTViFgAmmtG---LAnDke-EGlIDRFRSLPmSRsAlLtGRTlAdLVRnafiil

ZP06412839 pGVFvQTViFgsmmtG---LAtDke-aGlIDRFRALPmSRsAlLtGRTlAdLVRnafiil

NP_629116.1 AGIFAQTVtFatAgsaag-IAdDMq-kGlVDRFRSLPmARGAVLtGRTvAdLVqtsItll

AEM81020.1 AGIFAQTVtFatAgaGag-IAdDMn-kGlIDRFRSLPmARGAVLtGRTlAdLVqtaLtlv

EDY58361.1 AGIFAQTVtFatAssGag-IAdDMH-kGlIDRFRSLPmARGAVLtGRTfAdLVqtgLtlv

YP004804589 A--FAQTVtFatAgaGa---AdDMH-kGlIDRFRSLPmARGAVLtGRTlAdLVqtaLtlv

ADW03735.1 AGIFAQTVtFatAgaGag-IAdDMH-kGlIDRFRSLPmARGAVLtGRTlAdLVqtaLtlv

AGZ53017.1 VMLgtgvvLGfRf----rqGmiaSLmwvsVpvilGIAi-AaIvttValytaqtv-vvegv

YP872505.1 afLIVcaAMGvvh-------awtAalAlpfavLeGLAF-AacGmaattfmRSwq-dfewv

YP872504.1 tyLaViaAfGgvr-------sglAvcAvpIcLLlGLAc-AapiAafavrqetdt-sfsal

NP768671.1 -taIVAatLGyas-------wtsvLcAiptIaLtGLvF-AsLamVViSLaptyd-yfvfy

YP001770638.1 -sIIIAgvLGyaa-------wPsAfyAlpVIaLtGLAF-AsLamlVaALaRSyd-hfvfy

NP659764.1 -tIIVAtvLGyat-------wPsvvyvlpIIMLtGfAF-AsLamVVtALapSyE-yfify

YP002499215.1 -ttIVAamLGyaa-------wPsfLyAlpaIaLtGLAF-AsLamVVtALapSyd-yfvfy

ZP09087353.1 -ttIVAgtLGyaa-------wPsiLyAlpVIaLtGLAF-AsLamVVtALapSyd-yfvfy

YP004686285.1 aIMLVAgALGyaq-------mPgALlAlpVIvLa--AF-AaLamIVtALapSyd-ffmfy

YP558549.1 aIMLVAgALGyan-------fPsmLlAlpVIvLtGLAF-AsIamVVtALapSyd-ffmfy

YP001895399.1 aIMLVAgALGyan-------fPsmLlAlpVIvLtGLAF-AsIamVVtALapSyd-ffmfy

YP001808214.1 aIMLVAglLGyah-------fPsmLaAlpVIaLaGLAF-AstamIVtALapSyd-ffmfy

YP001764867.1 aIMLVAggLGyar-------fPsmLaAlpVIaLaGLAF-AsIamIVtALapSyd-ffmfy

YP_004581970.1 apLLVAiAfGldp-------tPgmLvipfVgfLtGLgF-glfGiwtsALVpSid-sfsyi

YP003396438.1 apLLVAiAfGlsp-------ewtALlvpiVcfvtGagF-AafGvaIaAIagtid-nfnyv

YP_004584405.1 VVVLlAavLGvgl-----TanPlkLvgaaVavviGaAFfAcLsiVIagLVlSrd-rlmgi

AAZ55486.1 IILaatipfGfRp------hvvgALggllIIsvFsvgv-gaLsyalalVsKdrhwlfwgv

WP_037627920.1 VtavcgfAaGWRt----HnGagglfaAFfVLLLFGLAm-gWLGAlIGvsVsSpq-aAaga

YP_004584320 VttvcglvaGWRv----qaGlgnALlAFlILLLFGLAm-sWvGAlIGvsVsnpE-vAaga

YP_004583032.1 VttvcglvaGWRv----qaGlgnALlAFlILLLFGLAm-sWvGAlIGvsVsnpE-vAaga

BAC70090.1 VLMvcglvvGWRp----HdGvPrALagiglLLLFrytl-AWvGtylGlVVRSar-tAarl

ACZ90809.1 IMVgagllvGWQp----HrGliptaqAFllMIvlryAl-sWvGvyVGlaVKndq-vAdam

YP001133205 VMaLtglAIGWRi----rnGvgeAvlAFalLMLFGfgm-iWf--lIGSLmRSvE-avngv

ABG10364.1 VMaLtglAIGWRi----rgsvgeAvlAFalLLLFGfgm-iWfGilIGSLmRtvE-avngv

WP_011692931.1 VtLLcglAIGWRi----tTGpgpALaAlaILLLFafAv-sWIGAVIaltaRSvE-vAqsl

YP004583513 IMLLVA--vGfRfG---dTtvPavLvgFalMLLFsyAF-AWvsAVIGlsVRnaE-aAqsa

YP480579 VMLLVglAvGfRfG---eTtvPaALagFglMLLFsyAF-sWLsAVIGlsVsSaE-aAqsa

YP001509262 IMLLVglAvGfRfG---dTtvPaiLagFgVMLiFsyAF-sWvsAVIGmsVRStE-aAqsg

ZP06412839 IMLLVglAvGfRfG---NTtvlaALagFglMLLFayAF-sWLsAVIGmsVgSaE-aAqsg

NP_629116.1 VLaIVAllvGWRtGSaepTnigrvLagFglLLLlGyAF-tWIGAlIGlsVRtpE-aAtsg

AEM81020.1 VLaIVAvivGWRi----HdGalkALgAFglLLLlGyAF-sWIGAlIGlsVRtpE-aAtsg

EDY58361.1 VLaLVAllvGWRvGSdgNTnagkvLaAFglLLLlGyAF-tWIGAlIGmsVRtpE-aAtss

YP004804589 VLagVAlivGWRt----HenigkvLlgFllLLLlGyAF-sWIGAlIGlIVRtpE-aAtsg

ADW03735.1 VLagVAliIGWRt----HenlgkvLlgFllLLLlGyAF-sWIGAlIGlVVRtpE-aAtsg

AGZ53017.1 elvqaiaifFstG-lvPLNsyPsWIQpVvahqPvSyAIaAMRgyamGg------------

YP872505.1 nlvlL-PMflfSatFYPLTayPtavahVvrWtPLyqAVDvqRa-Ll----------lGgW

YP872504.1 qrfvivPMflfSGtFFsIdQlPsWIrpVAyvtPLwhgVtltRd-vt----------sGvv

NP768671.1 qsLvLtPMvFLcGAvFPtSQMPdsfQhfAgllPLAhSVDlIRP-vm----------ler-

YP001770638.1 qtLFitPMlFLcGAvFPVNQlPseLknltsvlPLvhSIElIRP-vm----------lGr-

NP659764.1 qtLvLtPMlFLcGAvFPITQlPqtfQqVAqFlPLAhAIDlIRP-am----------lGr-

YP002499215.1 qtLFLtPMlFLcGAvFPaAQlPiafQhIAtvlPLAhSVDlVRa-am----------lGr-

ZP09087353.1 qtLvvtPMlFLcGAvFPVAQlPegfQqaAhFlPLAqAIDlIRP-am----------lG-r

YP004686285.1 qtLvmtPMllLSGvFFPLeQlPegvQaatkvlPLAhAValIRP-Lm----------lGr-

YP558549.1 qtLvLtPMllLSGvFFPVSQlPaiargVtevlPLAhSVDlMRP-am----------lGr-

YP001895399.1 qtLvLtPMllLSGvFFPVSQlPavargatqllPLAhAVDlMRP-am----------lGr-

YP001808214.1 qtLvLtPMllLSGvFFPLTQlPpiaQraAhvlPLAnAVElIRP-am----------lGr-

YP001764867.1 qtLvLtPMllLSGvFFPITQlPplaQraAqalPLAnAVElIRP-am----------lGr-

YP_004581970.1 isgvLtPlflvaGtFFPVSElPgWaralswaNPLyhcVElVRd-aa----------fGfa

YP003396438.1 qsglLtPlflIaGtFFPIdglPdaIQaIAqvNPLyhcVqlVRdlvv----------mGvd

YP_004584405.1 GqaimmPMfFaSnAlYPVdiMPsWvrvVsylNPLSylVDALRg-Ll-------igthshl

AAZ55486.1 qqtvLFPlvlLaGimlPVeggPgWLrtmsllNPLTyvVNAeRa-LF----------nGvW

WP_037627920.1 atvWLlPimYvSnAlvPVdsMPgWLQpVAeWNPMSavttAsRq-LFGN--PaApgaeGiW

YP_004584320 GtvWLFPvmYLSnAltPMSaMPgWLQpVAgWNPLSavttAcRq-LFGN--PtAsgaeniW

YP_004583032.1 GtvWLFPvmYLSnAltPMSaMPgWLQpVAgWNPLSavttAcRq-LFGN--PtAsgaeniW

BAC70090.1 apLtL-slTmvSnAlvPtdRMPvgLralceWNPLSsAaaAaRe-LFGN--PgApgpdaaW

ACZ90809.1 vpLgL-PfTmLSnAFvPtdgMPgWLrflAdWNPvStltaAsRe-LFGN--PgApsgdvaW

YP001133205 mftaLFPvTFLantFvPtepMPhWLrvIAeWNPvSslaqAMRe-LwGNGgP--apasaql

ABG10364.1 mftaLFPMTFLantFvPtepMPhWLrvIAeWNPvSslaqAMRe-LwGNGgP--aapdaql

WP_011692931.1 GlvWLFPvTFvSGAFvsVTslPgpLrlIAeWNPvTavatAaRe-LFGNtaPagfgvatgW

YP004583513 GfIWvFPlTFaSsAFvPVStMPgWLQafAehqPITvtIDAtRa-LF----------lGg-

YP480579 GfIWiFPlvFaSsAFarVetMPgWLQafArhqPvTvtVDAVRa-LF----------lGg-

YP001509262 GmIWvFPlvFaSsAFarVdtMPgWLQafAdhqPvTvtVDAVRa-LF----------lGg-

ZP06412839 GmIWiFPfvFaSssFaPVesMPgWLQafAkhqPvTctVDAVRa-LF----------lGg-

NP_629116.1 GiIWLFPvTFvSnAFvdpTQMtpWLrhVAeWNPfSavVqAcRe-LFGN--P-gvvqsdaW

AEM81020.1 GlIWLFPlTFISnAFvPsSKMPavLghIAdWNPfSatVqAsRq-LFGN--P-egvvsdaW

EDY58361.1 GlIWLFPvTFISnAFvdtShMtpWLrhIAeWNPfSatVqAcRv-LFaN--P-gqstsaaW

YP004804589 GlIWLFPlTFISnAFvdaNQMPafLrhIAeWNPfSatVqAcRe-LFGN-lPpgfvtpdaW

ADW03735.1 GlIWLFPlTFISnAFvdaNQMPtiLrhIAeWNPfSatVaAcRe-LFGN-vPpgfvtsdaW

AGZ53017.1 PvlsPMIgmLlWtigIcVacavpmaigYRRasth

YP872505.1 hAg--LLANcaYLavmSlag--vAVrrlaRvLlh

YP872504.1 epggvaLigvhlVfltAlagtrIAarnYRRRLeR

NP768671.1 gAdNaaL-HvgaLcvyAVLpffasIalFRRRLlR

YP001770638.1 stsgvgv-HifvLcAyAILpfffAtamlRRcLlR

NP659764.1 PAssmgL-HigaLciyAlLpffLsaallRRRLms

YP002499215.1 PAdsvgL-Hi--LcvyAVLpffLstallgRRLlR

ZP09087353.1 PAdsigL-HigaLsiyAVLpffvstalFRRRLmp

YP004686285.1 PSedvgL-HLavLgAyAVValvvclvllRRRLlR

YP558549.1 PveHaLv-HvavLaAyAlggfivsailFRRRMmK

YP001895399.1 PvdNaLL-HvavLaAyAlggfivsailFRRRMmK

YP001808214.1 PAtdvgL-HLavLagyAIggflvcawlFRRRMmR

YP001764867.1 PAtdiaL-HvavLagyAVggflLsawlFRRRMmR

YP_004581970.1 Pltd--LgHLgaLgvfAgImwlLAVrsmRRRLid

YP003396438.1 Plad--LgHLafLlvfglamwrLAIwrlRvRLid

YP_004584405.1 PldfvVLig-------SagvtiLAasallgRLaR

AAZ55486.1 dtsvvawgaLsaVvtavlglw-vgIvtmRRah--

WP_037627920.1 PteHPVMvsLsWslvIilVtapIAVkkFlRhtap

YP_004584320 PAdHPILAsvsWslAImlatvpLtVwkFvRRtsR

YP_004583032.1 PAdHPILAsvsWslAImlatvpLtVwkFvRRtsR

BAC70090.1 PltHPVtAsLlWaglLllIfvpLsVrrFaRagl-

ACZ90809.1 PlaHPVttvLlWsaALlVIfvpLslrsYmRR-gR

YP001133205 PlhYPVaStilWslALtaVfapfAlyaYKRRtgg

ABG10364.1 PlhHPVLAtvlWslvLtaVfapfAlraYaRRtgg

WP_011692931.1 PAeNaVLysvlcslAIigVfapLAIgqYRRiskR

YP004583513 PvasaLLqsLaWcvgmlVVfvpwcIrvYRRstgg

YP480579 PvvsvLLqsLaWclgLlVVfgpfAarvYRRsasR

YP001509262 PvaddLLlsLaWcg--llLfgpLAVraFRRdaag

ZP06412839 PvagdLLAaLaWca--lVVagpLAVraYRRgtah

NP_629116.1 PmqHPVwAtLtYsivIvlIfrtLAVrkYRRaag-

AEM81020.1 PmqHPaLAsvlWsllIiVLfrtLAVrkYRsata-

EDY58361.1 PmqHPVwAsLiYsvlIvVVfrtLAVrkYRsata-

YP004804589 PmrHPILAsviWsvvIlVVfrtLAVrkYRsata-

ADW03735.1 PmqHPILAsviWsvlIvVVfrtLAVrkYRsata-
