# Supplementary material for: Candidatus Frankia Datiscae Dg1, the Actinobacterial Microsymbiont of Datisca glomerata, Expresses the Canonical nod Genes nodABC in Symbiosis with Its Host Plant
Source: PLoS One. 2015 May 28;10(5):e0127630. doi: 10.1371/journal.pone.0127630 (PMC4447401; doi:10.1371/journal.pone.0127630)
Supplement: S6 Fig — All sequences from Dg1 are given in red. Sequences from β-proteobacteria where the rhizobial nodIJ genes evolved are given in green, sequences from α-proteobacteria are given in turquoise. Names of actinobacterial NltI/NltJ sequences the genes of which are part of a nodBCnltIJ operon are indicated in blue. The sequences from Streptomyces bottropensis are given in purple. All sequences used for the phylogenetic analysis are given in S6 Table. (PDF) [file pone.0127630.s006.pdf]

S6 Fig. Maximum Likelihood trees of (A) NodI and (B) NodJ proteins. All sequences from Dg1 are given in red. Sequences from β-proteobacteria where the rhizobial *nodIJ* genes evolved are given in green, sequences from α-proteobacteria are given in turquoise. Names of actinobacterial NltI/NltJ sequences the genes of which are part of a *nodBCnltIJ* operon are indicated in blue. The sequences from *Streptomyces bottropensis* are given in purple. All sequences used for the phylogenetic analysis are given in S6 Table.

(A) NodI

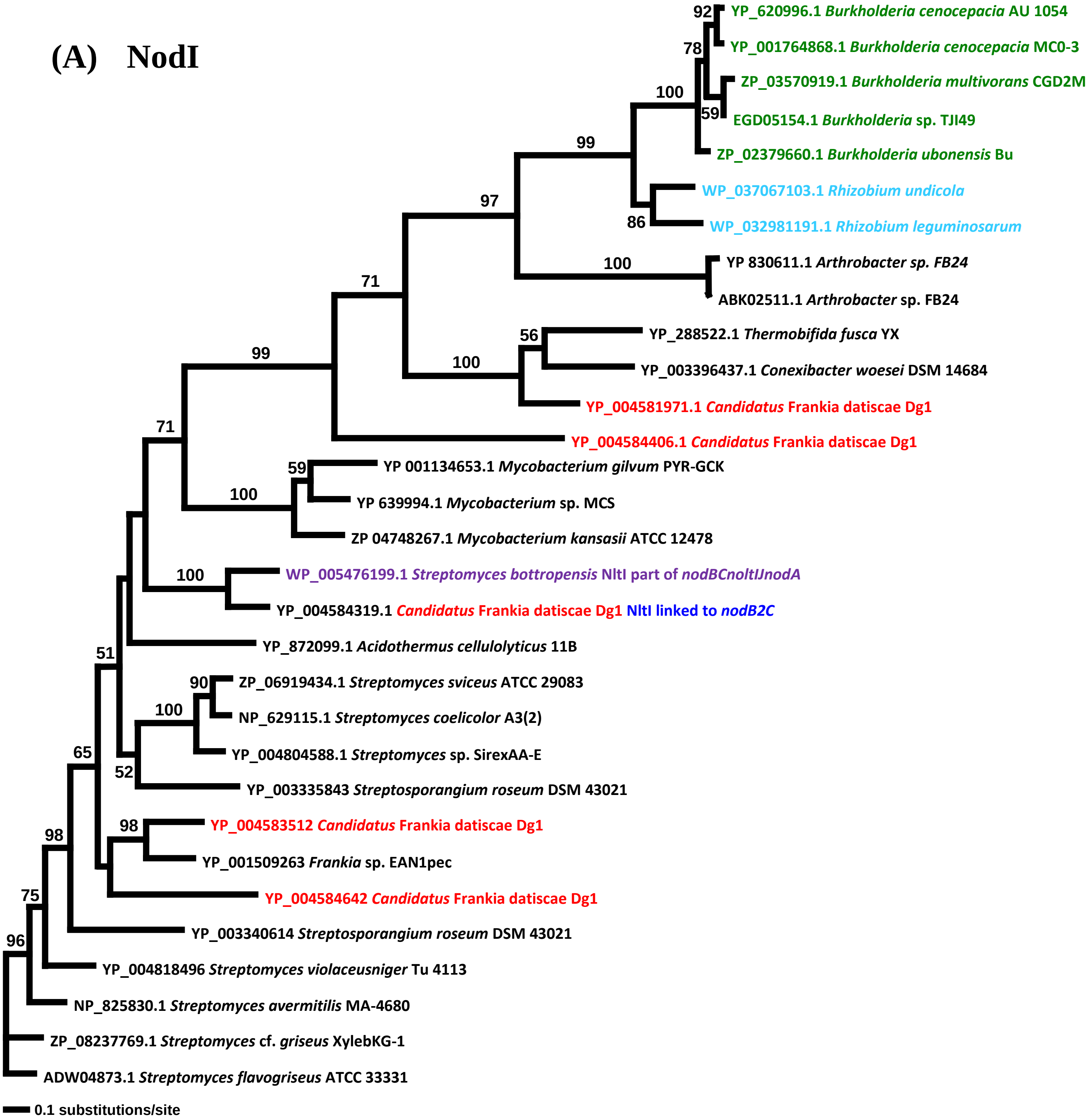

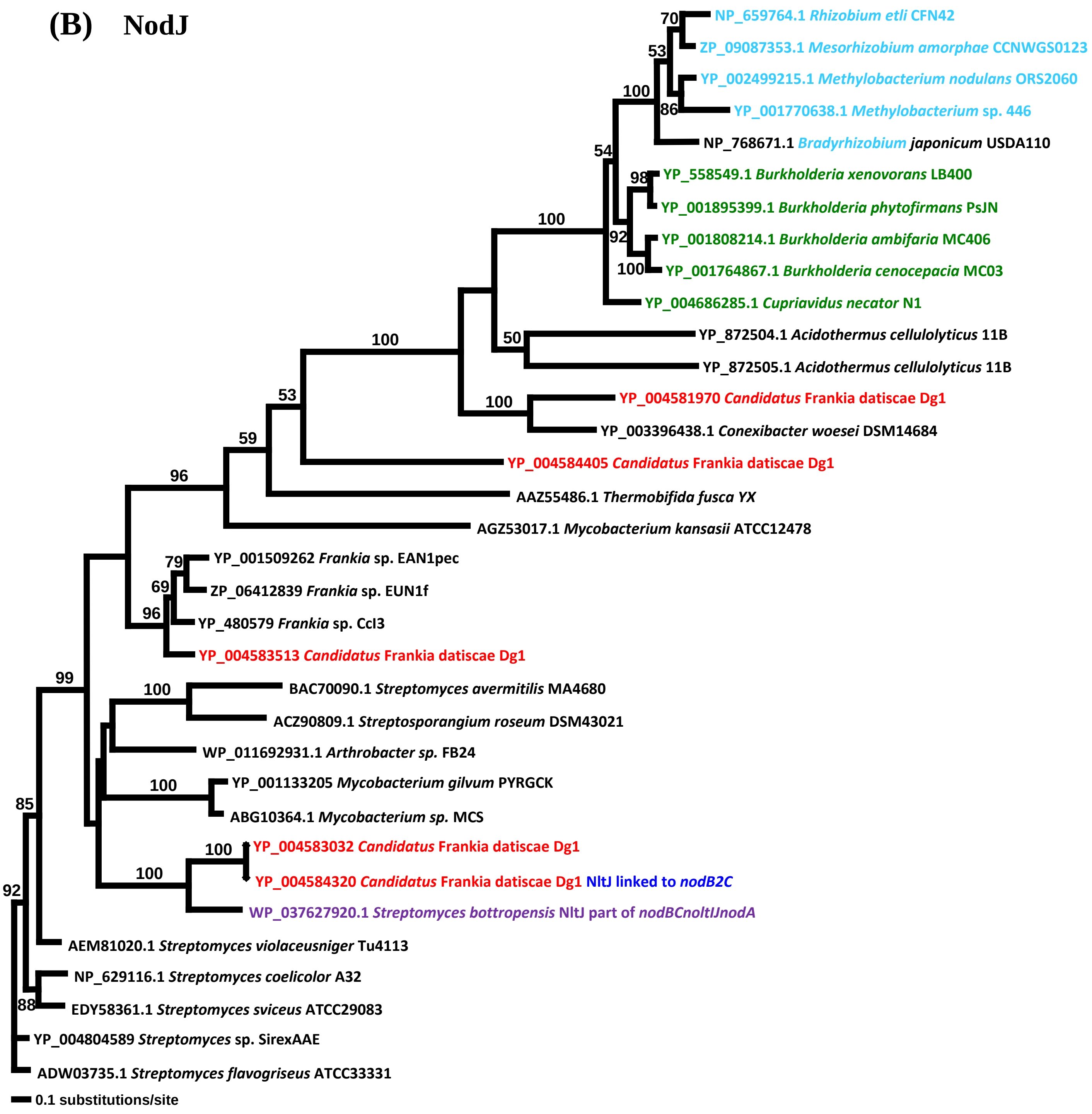

(B) NodJ
NP_659764.1 Rhizobium etli CFN42
ZP_09087353.1 Mesorhizobium amorphae CCNWGS0123
YP_002499215.1 Methylobacterium nodulans ORS2060
YP_001770638.1 Methylobacterium sp. 446
NP_768671.1 Bradyrhizobium japonicum USDA110
YP_558549.1 Burkholderia xenovorans LB400
YP_001895399.1 Burkholderia phytofirmans PsJN
YP_001808214.1 Burkholderia ambifaria MC406
YP_001764867.1 Burkholderia cenocepacia MC03
YP_004686285.1 Cupriavidus necator N1
YP_872504.1 Acidothermus cellulolyticus 11B
YP_872505.1 Acidothermus cellulolyticus 11B
YP_004581970 Candidatus Frankia datiscae Dg1
YP_003396438.1 Conexibacter woesei DSM14684
YP_004584405 Candidatus Frankia datiscae Dg1
AAZ55486.1 Thermobifida fusca YX
AGZ53017.1 Mycobacterium kansasii ATCC12478
YP_001509262 Frankia sp. EAN1pec
ZP_06412839 Frankia sp. EUN1f
YP_480579 Frankia sp. CcI3
YP_004583513 Candidatus Frankia datiscae Dg1
BAC70090.1 Streptomyces avermitilis MA4680
ACZ90809.1 Streptosporangium roseum DSM43021
WP_011692931.1 Arthrobacter sp. FB24
YP_001133205 Mycobacterium gilvum PYRGCK
ABG10364.1 Mycobacterium sp. MCS
YP_004583032 Candidatus Frankia datiscae Dg1
YP_004584320 Candidatus Frankia datiscae Dg1 NltJ linked to nodB2C
WP_037627920.1 Streptomyces bottropensis NltJ part of nodBCnoltIJnodA
AEM81020.1 Streptomyces violaceusniger Tu4113
NP_629116.1 Streptomyces coelicolor A32
EDY58361.1 Streptomyces sviceus ATCC29083
YP_004804589 Streptomyces sp. SirexAAE
ADW03735.1 Streptomyces flavogriseus ATCC33331
0.1 substitutions/site
70
53
100
86
54
98
100
92
100
100
50
100
100
53
59
96
79
69
96
99
100
100
100
100
85
92
88
