# Supplementary material for: Candidatus Frankia Datiscae Dg1, the Actinobacterial Microsymbiont of Datisca glomerata, Expresses the Canonical nod Genes nodABC in Symbiosis with Its Host Plant
Source: PLoS One. 2015 May 28;10(5):e0127630. doi: 10.1371/journal.pone.0127630 (PMC4447401; doi:10.1371/journal.pone.0127630)
Supplement: S5 Table — Palindromic Repeats were analyzed using the palindrome tool from EMBOSS (http://bips.u-strasbg.fr/EMBOSS/) with no mismatches and the following parameters: 1. Repeat units between 8 and 11 bases with up to a 3 base gap. 2. Repeat units between 12 and 19 bases with up to a 7 base gap. 3. Repeat units between 20 and 90 bases with up to a 20 base gap. 4. Repeat units less than 12 bases must occur at least 10 times in the genome. 5. Repeat units less than 20 bases must occur twice in the genome. Tandem repeats were analyzed with the MUMmer 3.13 package (http://www.tigr.org/software/mummer/) with the following parameters: Minimum match length = 20 bases. 2. It is assumed that one copy of a tandem repeat in a genome is not very significant unless it is long. Therefore, a genome-wide screen for the repeat used was added. The total number of bases incorporated into repeats for a particular repeat unit must total 50 or more bases. (DOCX) [file pone.0127630.s011.docx]

**S5 Table. Analysis of various genome characteristics in *Frankia* strains ACN14a CcI3, EaN1pec and Dg1.** Palindromic Repeats were analyzed using the palindrome tool from EMBOSS (http://bips.u-strasbg.fr/EMBOSS/) with no mismatches and the following parameters: 1. Repeat units between 8 and 11 bases with up to a 3 base gap. 2. Repeat units between 12 and 19 bases with up to a 7 base gap. 3. Repeat units between 20 and 90 bases with up to a 20 base gap. 4. Repeat units less than 12 bases must occur at least 10 times in the genome. 5. Repeat units less than 20 bases must occur twice in the genome. Tandem repeats were analyzed with the MUMmer 3.13 package (http://www.tigr.org/software/mummer/) with the following parameters: Minimum match length = 20 bases. 2. It is assumed that one copy of a tandem repeat in a genome is not very significant unless it is long. Therefore, a genome-wide screen for the repeat used was added. The total number of bases incorporated into repeats for a particular repeat unit must total 50 or more bases.

|  | **ACN14a** | **CcI3** | **EaN1pec** | **Dg1** |
| --- | --- | --- | --- | --- |
| Pseudogenes | 12 (0.18 %) | 50 (1.06 %) | 128 (1.78 %) | 325 (7.01 %) |
| Transposases | 33 (0.49 %) | 159 (2.82 %) | 198 (2.21 %) | 339 (7.40 %) |
| Tandem repeats | NA | 130 | 426 | 120 |
| Inverted repeats | NA | 53 | 3 | 43 |
| Total number of CDS | 6718 | 5559 | 8958 | 4579 |
| Genome size | 7497934 | 5433628 | 8982042 | 5323186 |
| G+C% | 72.83 % | 70.08 % | 71.15 % | 70.04 % |
