# Supplementary material for: Candidatus Frankia Datiscae Dg1, the Actinobacterial Microsymbiont of Datisca glomerata, Expresses the Canonical nod Genes nodABC in Symbiosis with Its Host Plant
Source: PLoS One. 2015 May 28;10(5):e0127630. doi: 10.1371/journal.pone.0127630 (PMC4447401; doi:10.1371/journal.pone.0127630)
Supplement: S7 Table — (DOCX) [file pone.0127630.s013.docx]

**S7 Table. List of *Frankia* strains used in the phylogenetic analysis and references.**

| ***Frankia* strain** | **Reference^a^** |
| --- | --- |
| OTU 51 | This study |
| OTU 138 | This study |
| OTU 200 | This study |
| OTU 366 | This study |
| OTU 404 | This study |
| OTU 488 | This study |
| OTU 734 | This study |
| OTU 770 | This study |
| OTU 797 | This study |
| Dg1 | gi334857119 |
| Cn endophyte | gi305257 |
| FE2 | gi3201705 |
| FE37 | gi3201707 |
| FE138 | gi5020208 |
| FE140 | gi5020204 |
| FE141 | gi5020205 |
| FE142 | gi5020206 |
| ACN14a | gi444304090 |
| EAN1pec | gi158107272 |
| Eul1c | gi311225233 |

^a^References are GenBank IDs.
